# P2X7 in Cancer: From Molecular Mechanisms to Therapeutics

**DOI:** 10.3389/fphar.2020.00793

**Published:** 2020-06-04

**Authors:** Romain Lara, Elena Adinolfi, Catherine A. Harwood, Mike Philpott, Julian A. Barden, Francesco Di Virgilio, Shaun McNulty

**Affiliations:** ^1^ Biosceptre (UK) Limited, Cambridge, United Kingdom; ^2^ Department of Medical Sciences, University of Ferrara, Ferrara, Italy; ^3^ Centre for Cell Biology and Cutaneous Research, Barts and the London School of Medicine and Dentistry, Queen Mary University of London, London, United Kingdom; ^4^ Centre for Cutaneous Research, Blizard Institute, Bart’s & The London School of Medicine and Dentistry, Queen Mary University of London, London, United Kingdom; ^5^ Biosceptre (Australia) Pty Ltd., Sydney, NSW, Australia; ^6^ Department of Morphology, Surgery and Experimental Medicine, Section of Pathology, Oncology and Experimental Biology, University of Ferrara, Ferrara, Italy

**Keywords:** P2X7, cancer, tumor progression, tumor microenvironment, therapeutics

## Abstract

P2X7 is a transmembrane receptor expressed in multiple cell types including neurons, dendritic cells, macrophages, monocytes, B and T cells where it can drive a wide range of physiological responses from pain transduction to immune response. Upon activation by its main ligand, extracellular ATP, P2X7 can form a nonselective channel for cations to enter the cell. Prolonged activation of P2X7, *via* high levels of extracellular ATP over an extended time period can lead to the formation of a macropore, leading to depolarization of the plasma membrane and ultimately to cell death. Thus, dependent on its activation state, P2X7 can either drive cell survival and proliferation, or induce cell death. In cancer, P2X7 has been shown to have a broad range of functions, including playing key roles in the development and spread of tumor cells. It is therefore unsurprising that P2X7 has been reported to be upregulated in several malignancies. Critically, ATP is present at high extracellular concentrations in the tumor microenvironment (TME) compared to levels observed in normal tissues. These high levels of ATP should present a survival challenge for cancer cells, potentially leading to constitutive receptor activation, prolonged macropore formation and ultimately to cell death. Therefore, to deliver the proven advantages for P2X7 in driving tumor survival and metastatic potential, the P2X7 macropore must be tightly controlled while retaining other functions. Studies have shown that commonly expressed P2X7 splice variants, distinct SNPs and post-translational receptor modifications can impair the capacity of P2X7 to open the macropore. These receptor modifications and potentially others may ultimately protect cancer cells from the negative consequences associated with constitutive activation of P2X7. Significantly, the effects of both P2X7 agonists and antagonists in preclinical tumor models of cancer demonstrate the potential for agents modifying P2X7 function, to provide innovative cancer therapies. This review summarizes recent advances in understanding of the structure and functions of P2X7 and how these impact P2X7 roles in cancer progression. We also review potential therapeutic approaches directed against P2X7.

## Introduction

Extracellular ATP is a danger associated molecular pattern (DAMP), which can act through the P2 receptor family including ionotropic P2X ion channel receptors and the G protein-coupled P2Y receptor family (Abbracchio and Burnstock, 1994; Brake et al., 1994; Valera et al., 1994; North, 2002; Burnstock, 2014). The P2X receptor family is formed by seven members able to assemble into homo- and hetero-trimers at the cell membrane (North, 2002; Burnstock, 2006). P2X family members are distinguished by their relative affinities to ATP, within the nanomolar range for P2X1 and P2X3, the low micromolar range for P2X2, P2X4, and P2X5 and the hundreds of micromolar range for P2X7 and also their rate of channel desensitization, milliseconds for P2X1 and P2X3, seconds for P2X2, P2X4, and P2X5 and no observable desensitization for P2X7, (Koshimizu et al., 1999; North and Surprenant, 2000; North, 2002; Burnstock, 2006; Jarvis and Khakh, 2009; Yan et al., 2010; Coddou et al., 2011).

P2X7 is characterized by its biphasic response to ATP stimulation. Upon activation with ATP for a relatively short period (2s or less), P2X7 opens a nonselective cation channel facilitating Na^+^ and Ca^2+^ influx and K^+^ efflux while prolonged ATP stimulation (4s or above) leads to the formation of a macropore permeable to molecules of <900 Da. However, recent reports have shown that ATP activated P2X7 receptors can open a NMDG^+^-permeable macropore within milliseconds and without progressive dilatation (Harkat et al., 2017; Pippel et al., 2017). Formation of this large molecular weight-permeable pore has been associated with the release of ATP in the microenvironment (Pellegatti et al., 2005; Adinolfi et al., 2010). Extracellular ATP helps to sustain macropore opening and is likely to drive several physiological and pathological mechanisms including chronic pain (Sorge et al., 2012), proliferation and activation of microglia (Bianco et al., 2006; Monif et al., 2009) and the death of enteric neurons during colitis (Gulbransen et al., 2012). In healthy tissues, P2X7 is expressed mainly in the central nervous system (CNS), peripheral nervous system (PNS), and in immune cells, which supports its emergence as a target for chronic pain and chronic inflammatory diseases (De Marchi et al., 2016). P2X7 is also overexpressed in a number of cancer types, potentially driving tumor development and survival (Adinolfi et al., 2002; Adinolfi et al., 2009; Adinolfi et al., 2012; Barden et al., 2014; Amoroso et al., 2015; Barden et al., 2016; Gilbert et al., 2019).

As a consequence of its ion channel activity, P2X7 is involved in driving several functions known to be important during tumor initiation and progression (Di Virgilio et al., 2018a). Yet, analysis of sequencing repositories from large tumor panels, including deep sequencing initiatives, does not highlight the preferential selection of mutation or copy number variation in the P2RX7 gene by tumor cells (Lawrence et al., 2014). This suggests that other receptor control mechanisms might modulate the critical role P2X7 plays during cancer progression. Extracellular ATP is present at low levels in healthy tissues but increases dramatically in response to tissue damage, inflammation, hypoxia, ischaemia and in the tumor microenvironment (TME). This increase in extracellular ATP has been attributed to cell death, cell stress, vesicular release, activation of pannexin and connexin, and to ATP-binding cassette (ABC) transporters (Pellegatti et al., 2008; Eltzschig et al., 2012; Galluzzi et al., 2017). In the TME, extracellular ATP is hydrolyzed into AMP by the ectonucleotidase CD39, AMP is then hydrolyzed into adenosine by the CD73 ectonucleotidase (Allard et al., 2017). While extracellular ATP acts as a DAMP to trigger proinflammatory immune responses, adenosine was found to be a potent immunosuppressor (Di Virgilio et al., 2018a). In this context, P2X7 collaboration with purinergic related proteins such as CD39 and Pannexin 1, was found to play an important role in the regulation of the antitumor immune response (de Andrade Mello et al., 2017).

The high extracellular ATP concentration found in the TME is sufficient to open the P2X7 macropore that could in turn lead to membrane depolarization and ultimately to cell death. To support tumor progression, cancer cells need to inhibit P2X7 macropore activity while retaining other receptor signaling functions. Several studies have described molecular mechanisms that lead to loss or attenuation of macropore function (See **Table 1**). These include the report of a misfolded form of P2X7 termed nonfunctional P2X7 (nfP2X7) that is characterized by the exposure of an extracellular epitope named E200 (Gidley-Baird and Barden, 2002; Barden et al., 2014; Gilbert et al., 2019). In this review we seek to describe how the interplay between P2X7 functions drive tumor progression and immune escape. We first present the topological features, functions and processes activated downstream of the P2X7 receptor before discussing how the interplay between these features may explain the paradoxical role of P2X7 in the tumor and the immune compartment. We then discuss the expression and role of P2X7 across multiple cancer types before reviewing the therapeutic approaches taken to target P2X7 to date.

**Table 1:** Channel modifications leading to the loss or gain of P2X7 macropore function.

Designation	Type of modification	Outcome	Reference
**Modification leading to Loss of P2X7 Macropore function**			
V76A	SNP	Partial loss of macropore function	(Stokes et al., 2010; Oyanguren-Desez et al., 2011)
R117W	SNP	Partial loss of channel and macropore function	(Roger et al., 2010; Wiley et al., 2011; Jiang et al., 2013)
G150R	SNP	Loss of macropore function	(Stokes et al., 2010)
E186K	SNP	Loss of channel and macropore function	(Roger et al., 2010; Wiley et al., 2011; Jiang et al., 2013)
N187D	SNP	Possible loss of function	(Chong et al., 2010b)
L191P	SNP	Partial loss of channel and macropore function	(Roger et al., 2010; Wiley et al., 2011; Jiang et al., 2013)
R276H	SNP	Loss of macropore function	(Stokes et al., 2010)
R307Q	SNP	Loss of macropore function	(Gu et al., 2004; Gartland et al., 2012; Jorgensen et al., 2012; Gu et al., 2015)
A348T	SNP	Mild increase of channel function in human P2X7 and mild decrease in channel function in rat P2X7	(Cabrini et al., 2005; Bradley et al., 2011)
T357S	SNP	Partial loss of channel and macropore function	(Cabrini et al., 2005; Shemon et al., 2006)
Q460R	SNP	Partial loss of channel and macropore function	(Cabrini et al., 2005; Stokes et al., 2010)
E496A	SNP	Loss of channel and macropore function	(Gu et al., 2001; Boldt et al., 2003; Cabrini et al., 2005; Roger et al., 2010; Sun et al., 2010; Gidlof et al., 2012; Wesselius et al., 2012; Jiang et al., 2013)
I568N	SNP	Loss of channel and pore function due to impaired P2X7 trafficking to the plasma membrane	(Wiley et al., 2003)
P2X7 variant B	Splice variant	Loss of macropore function (P2X7 variant A and B coexpression leads to gain of macropore function)	(Cheewatrakoolpong et al., 2005; Adinolfi et al., 2010)
P2X7 variant C	Splice variant	Assumed to have lost macropore function	(Cheewatrakoolpong et al., 2005; Benzaquen et al., 2019)
P2X7 variant D	Splice variant	Assumed to have lost macropore function	(Cheewatrakoolpong et al., 2005; Benzaquen et al., 2019)
P2X7 variant E	Splice variant	Assumed to have lost macropore function	(Cheewatrakoolpong et al., 2005; Benzaquen et al., 2019)
P2X7 variant F	Splice variant	Assumed to have lost macropore function	(Cheewatrakoolpong et al., 2005; Benzaquen et al., 2019)
P2X7 variant G	Splice variants	Loss of macropore function	(Cheewatrakoolpong et al., 2005; Benzaquen et al., 2019)
P2X7 variant H	Splice variants	Loss of macropore function	(Cheewatrakoolpong et al., 2005)
P2X7 variant J	Splice variant	Loss of macropore function (P2X7 variant J act as a dominant negative when coexpressed with P2X7 variant A leading to loss macropore function)	(Feng et al., 2006)
N187A	Impaired N-glycosylation	Loss of macropore function	(Lenertz et al., 2010)
R578Q	Impaired N-glycosylation	Loss of macropore function	(Wickert et al., 2013)
R277 or Y298	Proteolytic cleavage	Loss of macropore function following MMP-2 cleavage of P2X7 extracellular domain	(Young et al., 2018)
C-terminal tail	Binding partner	NMMHC-IIA	(Guo et al., 2007; Gu et al., 2009)
TM domains	Cholesterol binding	Loss of macropore function	(Robinson et al., 2014; Karasawa et al., 2017)
C362S and C363S	Prevent cholesterol inhibition rescue	Loss of macropore function	(Karasawa et al., 2017)
**Modification leading to Gain of P2X7 Macropore function**			
H155Y	SNP	Gain of macropore function	(Cabrini et al., 2005; Stokes et al., 2010; Oyanguren-Desez et al., 2011; Jiang et al., 2013)
A166G	SNP	Gain of macropore function	(Jiang et al., 2013)
H270R	SNP	Gain of macropore function	(Stokes et al., 2010)
R125	ADP-ribosylation of mouse P2X7	Gating of mouse P2X7 macropore	(Adriouch et al., 2008)
R206K, R276K, R277K (mouse)	Mutation	Gain of macropore function in mouse P2X7	(Adriouch et al., 2008)
TM domain	phosphatidylglycerol and sphingomyelin binding	Gain of macropore function	(Karasawa et al., 2017)

### Topology of the P2X7 Receptor

**Figure 1:**
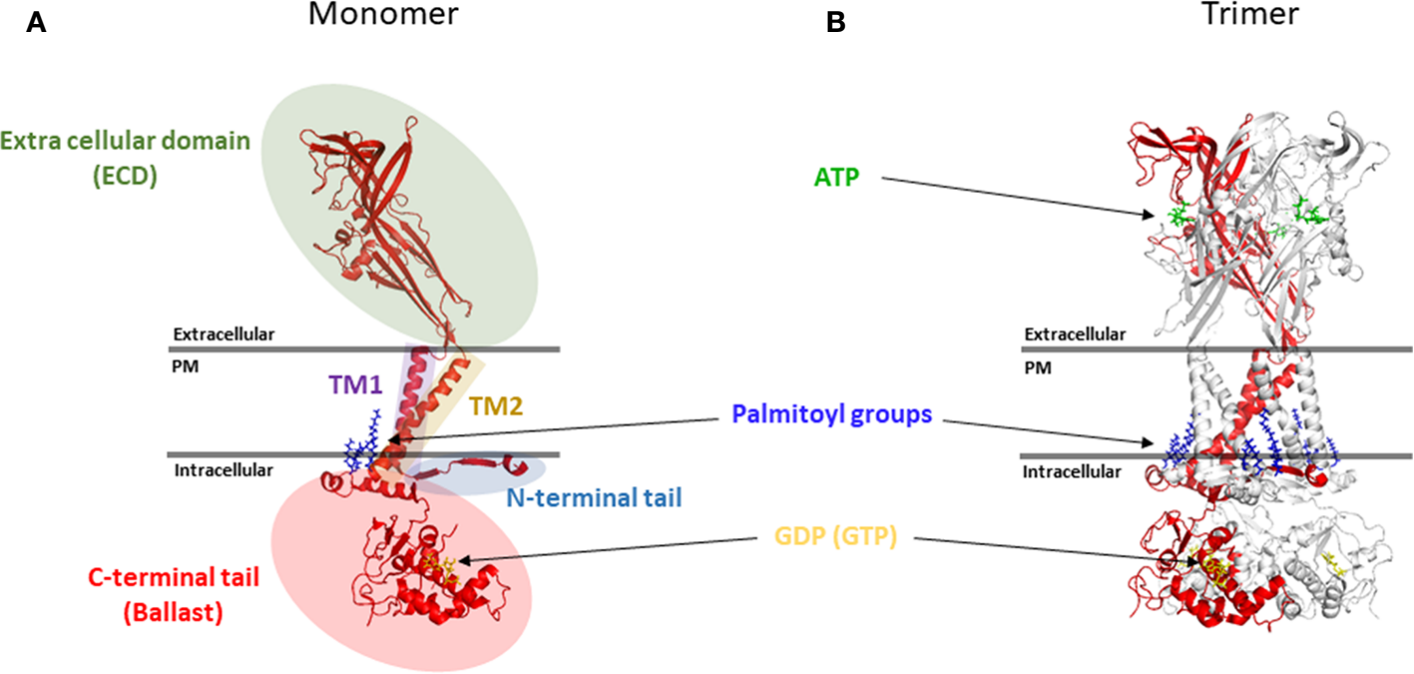
Topology of the P2X7 receptor. **(A)** Five main structural domains are present within each P2X7 monomer **(B)** Positioning of P2X7 monomer in the trimer. Rendering were generated from the rat P2X7 structure (PDB file 6U9W) (McCarthy et al., 2019) and positioned together with ATP, palmitoyl groups and GDP (GTP) molecules in relation to the plasma membrane (PM). Rendering were performed using PyMOL (https://pymol.org/).

The human *P2RX7* gene is located on chromosome 12 and encodes 13 exons that translate into a 595 amino acid protein. The location of *P2RX7* (12q24.31) is adjacent to the *P2RX4* gene, which is only 20Mbp downstream in the same reading direction (Buell et al., 1998a). Both genes are believed to be derived from successive gene duplications (Dubyak, 2007; Hou and Cao, 2016). Indeed, a recent report suggests that P2X7 was probably formed in lower vertebrates through the fusion of a P2X4-like gene with a Zn-coordinating cysteine-based domain (ZCD) coding exon (Rump et al., 2020). While heteromerisation of P2X7 and P2X4 *in vivo* is still controversial, both genes are found to be widely coexpressed (Guo et al., 2007; Kaczmarek-Hajek et al., 2012) and colocalize to act in concert in the regulation of the same physio-pathological functions (Kopp et al., 2019). Thirteen P2X7 splice variants have been identified to date (Benzaquen et al., 2019). While the resolution of the structure of human P2X7 has not yet been achieved, due to its propensity to aggregate, the partial structure of human P2X3 (Mansoor et al., 2016), zebra fish P2X4 (Kawate et al., 2009; Hattori and Gouaux, 2012; Kasuya et al., 2017), chicken P2X7 (Kasuya et al., 2017), panda P2X7 (Karasawa and Kawate, 2016; Karasawa et al., 2017), and more recently the full-length rat P2X7 (McCarthy et al., 2019) have been resolved. These have begun to reveal the molecular mechanism of ATP channel gating and the topology of the P2X7 trimer at the cell membrane. The P2X7 receptor is divided into five main structural domains (**Figure 1**).

#### N-Terminal Cytoplasmic Tail

A short N-terminal cytoplasmic tail of 25 amino acids (aa), which is anchored in the membrane *via* the palmitoylation of a cysteine residue at position 4 to form a cytoplasmic cap involved in the sensitisation of the channel to its agonist through key residues such as T15 and Q17 (Yan et al., 2010; McCarthy et al., 2019; Liang et al., 2019).

#### First Transmembrane Domain (TM1) and Extracellular Domain

The N-terminal cytoplasmic tail is followed by a first transmembrane domain named TM1 (aa 26 to 46) and a large extracellular domain of 282 aa (aa 47 to 329), which contains an inter-subunit ATP binding pocket (Hansen et al., 1997; Hattori and Gouaux, 2012; Karasawa et al., 2017; McCarthy et al., 2019). The extracellular domain also includes 5 disulfide bonds between cysteine residues 119–168, 129–152, 135–162, 216–226, and 260–269, which play a critical role in maintaining the conformation of the extracellular domain. These disulfide bridges are homologous across the other members of the P2X receptors which suggest that they form highly conserved protein folds (Hansen et al., 1997). Several post-translational modifications have been identified in the P2X7 extracellular domain which can regulate receptor functions; these include ADP-ribosylation of R125 on murine P2X7 that is involved in the gating of the murine P2X7 receptor (Schwarz et al., 2012) and N-linked glycosylation of 5 asparagine residues (D187, D202, D213, D241, and D284) with D187 N-linked glycosylation playing a key role in the regulation of P2X7 signaling through the MAP kinase pathway (Lenertz et al., 2010).

#### Second Transmembrane Domain (TM2)

The extracellular region is followed by a second transmembrane domain named TM2 (aa 330 to 349), which contains several key pore-lining residues. These include S342, which sits at the narrowest part of the channel to regulate its gating (Pippel et al., 2017; McCarthy et al., 2019). Phosphorylation of the adjacent Y343 also regulates channel gating which further emphasizes the importance of those two residues during channel gating (Kim et al., 2001; Liang et al., 2019).

#### C-terminal Cytoplasmic Tail

**Figure 2:**
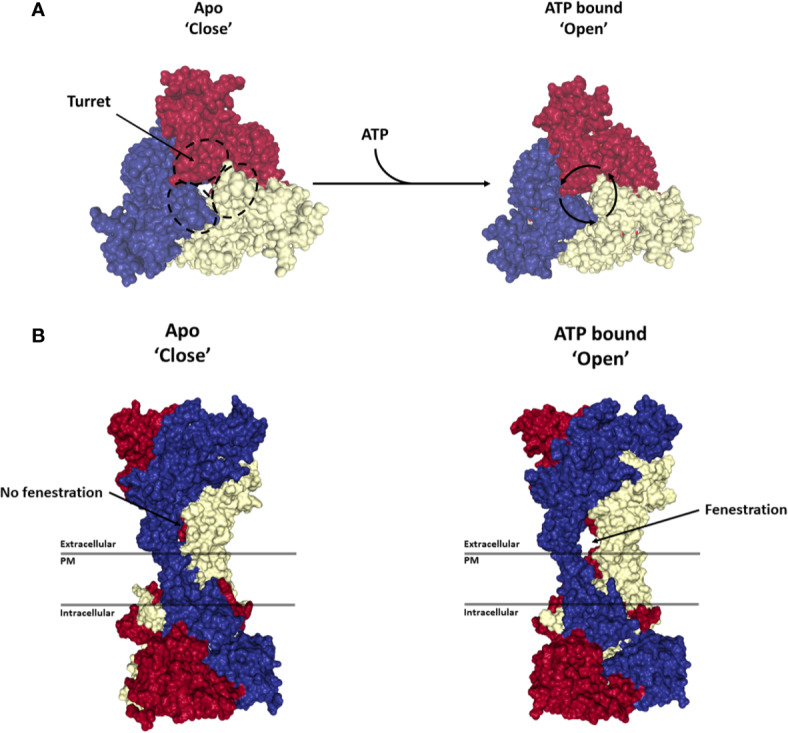
Activation of P2X7 receptor. Structures show the top **(A)** and side view **(B)** of the P2X7 trimer in the Apo-closed state (PDB file: 6U9V) and ATP bound open state (PDB file: 6U9W). Each monomer is represented in a different color (blue, red or yellow). ATP binding to the trimer leads to a rotation of each monomer around its respective turret leading to the opening of a side fenestration and an ion channel through the plasma membrane (PM). Renderings were generated from the rat P2X7 structure (McCarthy et al., 2019) using PyMOL (https://pymol.org/).

The second transmembrane domain is flanked by a long C-terminal cytoplasmic tail of 245 aa (aa 350 to 595), which acts as a ballast and is involved in the modulation of macropore opening (Surprenant et al., 1996; Cheewatrakoolpong et al., 2005; Costa-Junior et al., 2011; McCarthy et al., 2019). The beginning of the C-terminal tail contains a cysteine rich region that is palmitoylated on at least five residues (C362, C363, C374, C377, and S360) and plays an important role in trafficking of the receptor to the cell membrane and in preventing receptor desensitization (Gonnord et al., 2009; McCarthy et al., 2019). These multiple palmitoylated residues act as a hinge allowing each C-terminal tail to create two zinc binding sites and a guanosine diphosphate or triphosphate (GDP/GTP) binding site. From a functional perspective, the C-terminal tail has been shown to be required for the opening of the macropore (Surprenant et al., 1996; Adinolfi et al., 2010). Recent reports have also shown that pore opening requires movement of the TM2 helix which is directly linked to the globular ballast folded underneath (Pippel et al., 2017; McCarthy et al., 2019). This suggests that a significant movement of the ballast is needed to allow for the macropore to form (See **Figures 1** and **2**). Given the presence of GDP/GTP binding sites in the P2X7 C-terminal tail, it is tempting to hypothesize that such a structural rearrangement may involve an exchange between GDP and GTP. This type of mechanism has already been extensively characterized for GTPase enzymes, which promote the hydrolysis of GTP to GDP to trigger protein interaction and signaling cascades (Bos et al., 2007). This switch could either be mediated by P2X7 itself or through accessory proteins such as GTPase-activating proteins (GAPs) and guanine nucleotide exchange factors (GEFs). However, further work is needed to confirm this hypothesis.

## P2X7 Possesses Ion Channel Functionality

P2X7 is distinguished from other P2X family members by its relative insensitivity to its natural agonist ATP (EC_50_ above 80 −100 µM) (Surprenant et al., 1996). ATP binding to the inter-subunit pocket leads to a rotation of each subunit around its turret and a widening of the lower body of the P2X7 trimer. This conformational rearrangement is associated with the opening of a lateral fenestration in the lower body of the trimer that in turn allows influx of Ca^2+^ and Na^+^ ions into and the efflux of K^+^ ions from the cell (**Figures 2A, B**) (Karasawa and Kawate, 2016; McCarthy et al., 2019). Several well characterized small molecule inhibitors have been reported to inhibit this mechanism through binding to an allosteric pocket located in between the turret of each subunit, thereby preventing the closure of the turret necessary for the opening of the channel (**Figures 2A, B**) (Karasawa and Kawate, 2016). Observations made using rat P2X7 suggest that the C-terminal cytoplasmic tail does not significantly regulate receptor current facilitation, desensitization, affinity for ATP or its receptor ion selectivity (McCarthy et al., 2019).

**Table 2:** Studies confirming the presence of P2X7.

Cancer type	Studies confirming the presence of P2X7
Prostate	(Slater et al., 2004a; Slater et al., 2005; Maianski et al., 2007; Adinolfi et al., 2009; Barden et al., 2009; Ravenna et al., 2009; Adinolfi et al., 2010; Barden et al., 2014; Ghalali et al., 2014; Qiu et al., 2014; Solini et al., 2015; Barden et al., 2016; Gilbert et al., 2019)
Lung	(Barden et al., 2009; Takai et al., 2012; Jelassi et al., 2013; Barden et al., 2014; Takai et al., 2014; Schmid et al., 2015; Gilbert et al., 2019)
Kidney	(Liu et al., 2015; Gilbert et al., 2019)
Colorectal	(Coutinho-Silva et al., 2005; Barden et al., 2009; Li et al., 2009; Kunzli et al., 2011; Bian et al., 2013; Barden et al., 2014; Barden et al., 2016; Qian et al., 2017; Gilbert et al., 2019; Zhang et al., 2019)
Gastric	(Barden et al., 2009; Barden et al., 2014; Calik et al., 2020)
Breast	(Slater et al., 2004b; Barden et al., 2009; Li et al., 2009; Tafani et al., 2010; Jelassi et al., 2011; Huang et al., 2013; Jelassi et al., 2013; Barden et al., 2014; Chadet et al., 2014; Zheng et al., 2014; Tan et al., 2015; Draganov et al., 2015; Xia et al., 2015; Barden et al., 2016; Park et al., 2016; Gilbert et al., 2019; Park et al., 2019)
Cutaneous squamous-cell and basal-cell carcinomas	(Greig et al., 2003; Slater and Barden, 2005; Barden et al., 2016; Yang et al., 2016; Gilbert et al., 2017)
Melanoma	(Slater et al., 2003; White et al., 2005; Shankavaram et al., 2009; Reinhold et al., 2012; Barden et al., 2014; Roger et al., 2015; Gilbert et al., 2019)
Leukemia and lymphoma	(Peng et al., 1999; Gu et al., 2000; Adinolfi et al., 2002; Wiley et al., 2002; Barden et al., 2003; Zhang et al., 2003; Zhang et al., 2004; Yoon et al., 2006; Barden et al., 2009; Constantinescu et al., 2010; Chong et al., 2010b; Chong et al., 2010b; Wang et al., 2011; Barden et al., 2014; Chen et al., 2014; Ledderose et al., 2016; Salaro et al., 2016; Salvestrini et al., 2017)
Neuroblastoma	(Kaiho et al., 1998; Schrier et al., 2002; Raffaghello et al., 2006; Sun, 2010; Gutierrez-Martin et al., 2011; Bianchi et al., 2014; Amoroso et al., 2015; Gomez-Villafuertes et al., 2015; Wilkaniec et al., 2017; Ulrich et al., 2018; Gilbert et al., 2019)
Glioma	(Wei et al., 2008; Tamajusuku et al., 2010; Ryu et al., 2011; Tafani et al., 2011; Gehring et al., 2012; Fang et al., 2013; Barden et al., 2014; Gehring et al., 2015; McLarnon, 2017; Ji et al., 2018; Bergamin et al., 2019; Kan et al., 2019)
Ovarian	(Barden et al., 2014; Vazquez-Cuevas et al., 2014; Gilbert et al., 2019)
Cervical and endometrial	(Feng et al., 2006; Feng et al., 2006; Li et al., 2006; Li et al., 2007; Barden et al., 2009; Barden et al., 2014)
Bladder	(Barden et al., 2009; Barden et al., 2014; Hu et al., 2016; Gilbert et al., 2019)
Papillary Thyroid	(Ruggeri et al., 2002; Solini et al., 2008; Barden et al., 2009; Li et al., 2009; Dardano et al., 2009; Gu et al., 2010; Barden et al., 2014; Kwon et al., 2014)
Pancreatic	(Kunzli et al., 2007; Mistafa and Stenius, 2009; Barden et al., 2014; Giannuzzo et al., 2015; Giannuzzo et al., 2016; Choi et al., 2018; Gilbert et al., 2019)
Bone (osteosarcoma, Ewing sarcoma, chondromyxoid fibroma)	(Gartland et al., 2001; Alqallaf et al., 2009; Barden et al., 2009; Liu and Chen, 2010; Adinolfi et al., 2012b; Barden et al., 2014; Giuliani et al., 2014; Zhang et al., 2019a)
Head and neck	(Barden et al., 2009; Barden et al., 2014; Bae et al., 2017)
Testicular	(Barden et al., 2009; Barden et al., 2014)
Esophageal	(Barden et al., 2009; Barden et al., 2014; Santos et al., 2017)
Trophoblastic	(Barden et al., 2009; Barden et al., 2014)
Mesothelioma	(Barden et al., 2009; Barden et al., 2014; Amoroso et al., 2016)

Structural analysis of P2X family members together with experimental characterization and mathematical modeling have provided insights into the transition states of the P2X7 receptor following ATP binding. While occupation of all three ATP binding sites on the trimer leads to a symmetric conformational rearrangement and to channel gating, the binding of a single ATP molecule leads to an asymmetric rearrangement of the trimer leaving the fenestration closed and decreasing the affinity of the second and third ATP binding sites. Upon binding of a second ATP molecule, a low conductance is observed with a further decrease in the affinity of the third ATP binding site (Yan et al., 2010). These observations explain why P2X7 is relatively insensitive to ATP compared to other P2X family members. The functional implication is that P2X7 ion channel activation is only achieved when the level of ATP is significantly elevated in the extracellular compartment. Such conditions can be found in the TME, during inflammatory responses and in chronic inflammatory diseases. In these settings, P2X7 mediated calcium signaling was shown to trigger intracellular signaling and changes in gene expression that are critical for cancer cell survival and proliferation (Di Virgilio, 2015; Di Virgilio et al., 2017; Di Virgilio et al., 2018a). Indeed, overexpression of full length P2X7 was shown to promote tumor growth (Jelassi et al., 2011; Adinolfi et al., 2012a), while P2X7 blockade was shown to inhibit neoplastic growth in several experimental models (Adinolfi et al., 2012a; Adinolfi et al., 2015a; Amoroso et al., 2015; Amoroso et al., 2016; Giannuzzo et al., 2016; De Marchi et al., 2019; Zhang et al., 2019a).

## P2X7 Activation Can Open a Transmembrane Macropore

Evidence for ATP-induced dye uptake was first identified in the 1970s and 80s (Rozengurt and Heppel, 1975; Cockcroft and Gomperts, 1979; Steinberg et al., 1987) but it was only a decade later that P2X7, previously known as P2Z, was shown to play a key role in the formation of a transmembrane dye-permeable macropore (Surprenant et al., 1996; Virginio et al., 1999). The formation of a transmembrane pore is not believed to be limited exclusively to P2X7. Other ion channels including P2X2, P2X4, TRPV1 and ASICs have also been shown to increase conductance and allow molecules of <900 Da into the cell (Khakh and Lester, 1999; Li et al., 2015). However, while P2X2, P2X4, TRPV1, and ASICs undergo a “channel to pore dilatation” upon prolonged activation, P2X7 pore opening is almost instantaneous (within milliseconds) (Li et al., 2015; Pippel et al., 2017).

While demonstration of the P2X7 macropore has been described extensively *in vitro* (Di Virgilio et al., 2018b), opening of the macropore *in vivo* remains to be demonstrated. Opening of the macropore allows the flow of ions across the membrane leading to rapid membrane depolarization and ultimately to cell death. *In vitro* assessment of macropore opening is typically performed by measuring the increase in fluorescence associated with the influx of ethidium^+^ (314 Da), YO-PRO-1^2+^ (375 Da), Lucifer yellow (444 Da), DAPI (277 Da) or the current associated with N-methyl-D-glucamine^+^ (NMDG^+^, 195 Da) influx (Di Virgilio et al., 2019). Given the significant risk that an open macropore may have on cell viability, the low sensitivity of P2X7 for ATP provides a first mechanism of protection for cells. Furthermore, P2X7 macropore opening has been shown to be reversible upon the removal of ATP within 10 to 15 min from initiation of stimulation in a mouse macrophage cell line, however reversibility may be cell type specific (Di Virgilio et al., 1988; Di Virgilio et al., 2018b). This reversible closure of the macropore can potentially provide a second mechanism to protect cells from P2X7-mediated cell death. Although the advantages of gated opening of the P2X7 pore are not well understood, such transient mechanisms could allow small molecules, such as peptides and micro-RNA, to enter or leave the cell (Di Virgilio et al., 1988) as observed for connexins and pannexins at cell to cell junctions (Esseltine and Laird, 2016). The demonstration that P2X7 is permeable to the large natural cation spermidine, a polyamine molecule involved in cell survival, supports this hypothesis (Harkat et al., 2017). Since opening of a high conductance transmembrane conduit has inevitable dire consequences on cell survival, it is likely that under more physiological conditions, localized opening of the P2X7 macropore occurs that will perturb cytoplasmic ion homeostasis at discrete sites only (Franceschini et al., 2015).

The mechanism(s) leading to macropore opening have been the subject of much controversy. Several hypotheses have been proposed to support P2X7 dilatation in response to sustained agonist stimulation and the involvement of associated proteins such as pannexin-1 (Reviewed in (Di Virgilio et al., 2017; Di Virgilio et al., 2018b)). However, recent analysis of human and rat P2X7 electrophysiological activity in response to agonist shows that the macropore function is intrinsic to P2X7 and does not involve progressive pore dilatation (Harkat et al., 2017; Pippel et al., 2017). Extracellular ATP is present at concentrations in the low nanomolar range in healthy tissue but can increase dramatically upon tissue damage, inflammation or during tumor development to the tens to hundreds of micromolar range and reach the high activation threshold of 0.3 to 0.5mM for P2X7 (Di Virgilio, 2015; Morciano et al., 2017; Di Virgilio et al., 2018b). We demonstrated that antibodies raised against the E200 epitope of P2X7 bind specifically to cancer cell lines that are devoid of macropore function but that retain P2X7 ion channel function (Barden et al., 2003; Gilbert et al., 2019). In these cell lines, overexpressed wild type P2X7 rescues macropore function but is not detected by antibodies that target E200 supporting the specific exposure of E200 epitope by nfP2X7 (Gilbert et al., 2019). E200 specific antibodies were used to show that nfP2X7 is present intracellularly and can be released to the plasma membrane upon stimulation with KM11060, a small molecule corrector that increases the secretion rate of misfolded proteins to the membrane as shown previously for the Cystic Fibrosis Transmembrane Receptor (CFTR) mutants (Robert et al., 2008). nfP2X7 was also shown to be necessary for cell survival (Gilbert et al., 2019). Hence, these data suggest that to sustain growth without compromising survival, cancer cells expressing high levels of P2X7 have developed a mechanism to inhibit macropore function while retaining ion channel functionality (Gilbert et al., 2019).

Although mutations of *P2X7,* such as E496A, have been shown to abrogate the macropore (Gu et al., 2001; Ghiringhelli et al., 2009), we and others have demonstrated that P2X7 is expressed at the cell membrane in the absence of mutations such as E496A. This suggests that additional mechanisms are involved in protecting cancer cells (Gilbert et al., 2019). Indeed, several other mechanisms have been shown to modulate P2X7 macropore activity. These include splice variants (Benzaquen et al., 2019), N-glycosylation (Lenertz et al., 2010), ADP-ribosylation (Adriouch et al., 2008), proteolytic cleavage (Young et al., 2018), and interactions with binding partners or other P2X family members (Guo et al., 2007; Gu et al., 2009). They also include interactions with cholesterol in the membrane (Robinson et al., 2014; Karasawa et al., 2017), that may attenuate or abrogate P2X7 macropore formation (summarized in **Table 1**). One or more of these mechanisms has the potential to protect cells that possess P2X7 receptors in a high ATP microenvironment over a sustained time period.

## P2X7 as a Scavenger Driving Phagocytosis

Further to its ability to open an ion channel and macropore, P2X7 has also been shown to act as a scavenger receptor promoting phagocytosis of nonopsonised targets such as apoptotic cells, latex beads and live or dead bacteria (Staphylococcus *aureus* and Escherichia *coli*) under serum free conditions (Gu B. et al., 2010; Gu et al., 2011; Gu et al., 2012). Binding of P2X7 to the nonmuscle myosin heavy chain (NMMHC-IIA) was shown to be required for the engulfment of particles (Gu B. et al., 2010; Gu et al., 2011). This function is inversely correlated with P2X7 macropore opening, which requires NMMHC-IIA dissociation from P2X7 upon ATP stimulation (Gu et al., 2009; Gu B. et al., 2010; Gu et al., 2011). P2X7 mediated phagocytosis was first demonstrated in monocytes and macrophages before being extended to microglia cells in the CNS where it plays a critical role in the clearance of nonopsonised particles and debris involved in neuro-inflammation (Wiley et al., 2011; Gu and Wiley, 2018; Janks et al., 2018). The ability of the P2X7-NMMHC-IIA complex to act as a scavenger suggests another P2X7-mediated role for macrophages infiltrated in the TME where P2X7 would recognize and promote the phagocytosis of apoptotic cells and potentially support the presentation of associated antigen to immune effector cells. Indeed, during inflammation and in the TME, the extracellular ATP concentration is significantly higher than in healthy tissues (Di Virgilio et al., 2018b). Under these conditions, P2X7 (as well as P2X4) stimulation potentiates phagocytosis by inflammatory macrophages (Zumerle et al., 2019).

## Downstream Effect of P2X7 Activation

Several signaling pathways are activated downstream of P2X7 including hypoxia and proinflammatory pathways *via* hypoxia-inducible factor 1a (HIF-1α), nitric oxide synthase (NOS2), cyclooxygenase 2 (COX2) and acute phase protein pentraxin-3 (PTX3) (Tafani et al., 2010). Downstream effects of P2X7 activation also include survival and proliferation pathways such as the NF-κB (Tafani et al., 2010; Liu et al., 2011), NFATc1 (Adinolfi et al., 2009), Phosphoinositide 3-kinases (PI3Ks) (Bian et al., 2013; Amoroso et al., 2015), mitogen-activated kinases (MAPK) (Bradford and Soltoff, 2002; Amstrup and Novak, 2003), phospholipase A2, C and D (Humphreys and Dubyak, 1996; Coutinho-Silva et al., 2003; Andrei et al., 2004) acid sphingomyelinase (Bianco et al., 2009) and myc oncogene (Amoroso et al., 2015).

The regulation of downstream signaling pathways by P2X7 is defined at three levels. First, the concentration and duration of agonist exposure. While acute exposure to relatively high amounts of ATP has been shown to activate PI3K/AKT and AMPK-PRAS-40-mTOR signaling pathways to trigger tumor cell death (Bian et al., 2013), lower and chronic activation has been shown to activate the NOD-like receptor containing a pyrin (NLRP3) inflammasome pathway. Second, regulation may involve the interaction of P2X7 with other membrane proteins, such as other P2X family members or Pannexin 1. Third, interaction with scaffolding proteins associated with the cytoskeleton such as NMMHC-IIA (Gu et al., 2009). Indeed, the P2X7 receptor signaling complex has been shown to regulate the macropore and interact with the actin cytoskeleton machinery (Kim et al., 2001). These three levels of regulation are involved in several mechanisms that are key to cancer progression.

### Cell Death

The early discovery of the P2X7 macropore drove interest in P2X7 as a receptor capable of mediating cell death through disruption of intracellular homeostasis and cytolysis leading to necrosis (Zanovello et al., 1990; Surprenant et al., 1996). However, many reports have highlighted cell death mechanisms associated with P2X7 activation that are typical of an apoptotic pathway. These include membrane depolarization, redistribution of phosphatidylserine (PS) to the outer membrane, caspase 3, 8 and 9 activation and membrane blebbing (Zanovello et al., 1990; Ferrari et al., 1997a; Humphreys et al., 2000; Mackenzie et al., 2005). Interestingly, the recent resolution of the rat P2X7 structure has highlighted the presence of a putative phospholipid in a perpendicular position to the transmembrane helices in the middle of the plasma membrane that led McCarthy and colleagues to suggest that P2X7 might possess an intrinsic flippase-like function driving PS redistribution to the outer membrane (McCarthy et al., 2019).

### Proliferation

The notion that the P2X7 receptor, together with mediating cytotoxic activity, also exerts a proliferative drive dates back to 1999, when in a pivotal paper from Baricordi and colleagues, it was demonstrated that transfection with the receptor conferred a proliferative advantage to leukaemic cell lines growing in the absence of serum (Baricordi et al., 1999). In the following years, the trophic activity of P2X7 was associated with an increase in mitochondrial function leading to an augmented production of intracellular ATP (Adinolfi et al., 2005) and, in general, to increased levels of calcium inside cellular stores (Adinolfi et al., 2009).

P2X7 calcium channel activity plays a central role in driving proliferation and is shared by the human splice variant P2X7B that retains ion channel activity while losing the ability to form a macropore (Adinolfi et al., 2010). When transfected in the osteosarcoma cell line Te85, P2X7B causes an increase in proliferation that is even greater than that attributable to transfection of P2X7A (Giuliani et al., 2014). Several calcium-related intracellular pathways involved in cell proliferation were shown to be activated by the P2X7 receptor. These include the JNK/MAPK, PI3K/AKT/myc and HIF1α-VEGF pathways (Adinolfi et al., 2012b; Adinolfi et al., 2015b; Di Virgilio et al., 2016; Di Virgilio and Adinolfi, 2017; Orioli et al., 2017). Among these, the nuclear factor of activated T cells (NFAT) pathway is one of the best characterized. NFAT is a calcium-calcineurin activated nuclear factor involved in the proliferation of several cell types including T lymphocytes where P2X7-dependent activation of NFAT was shown to facilitate clonal expansion and IL-2 secretion thus positively influencing lymphocytic growth (Yu et al., 2010). NFATc1 mediated pathway was also responsible for increased proliferation of HEK-293 (Adinolfi et al., 2009; Adinolfi et al., 2010) and Te85 cells transfected with both P2X7A and B isoforms (Giuliani et al., 2014) and was found to be upregulated in xenografts overexpressing the receptor (Adinolfi et al., 2012a).

Association between P2X7, proliferation and survival has been demonstrated in several primary cell types including osteoblasts (Grol et al., 2013; Agrawal et al., 2017; Yang et al., 2019), microglia (Monif et al., 2009), and CD8^+^ memory T cells (Borges da Silva et al., 2018) where it supports physiological functions such as inflammatory responses and osteogenesis. However, increased P2X7 activity was also linked with increased proliferation and survival in many cancer cell types including pancreatic cancer (Giannuzzo et al., 2016), leukemia (Salaro et al., 2016) and glioma (Ji et al., 2018). Indeed, reports showing enhanced P2X7 expression in tumor tissues compared with normal tissue across many cancer types suggest a role for P2X7 during tumorigenesis (See section: The function and expression of P2X7 across cancer types below). Following the demonstration of the growth-promoting activity of P2X7 *in vitro*, it was also shown in several studies that P2X7 can influence *in vivo* tumor growth and that consequently, down-modulation of the receptor by silencing or pharmacological intervention can reduce tumor burden in preclinical models (Di Virgilio et al., 2018a). Adinolfi and colleagues first demonstrated the growth-promoting activity of P2X7 in four different tumor models in mice including HEK-293 cells and ACN neuroblastoma derived xenografts and syngeneic colon carcinoma and melanoma models (Adinolfi et al., 2012a). Several studies have confirmed these data and extended them, demonstrating that P2X7 blockade leads to a general reduction of tumor burden in neuroblastoma (Amoroso et al., 2015; Ulrich et al., 2018), mesothelioma (Amoroso et al., 2016), melanoma (Hattori et al., 2012; Adinolfi et al., 2015a), glioma (Ji et al., 2018), osteosarcoma (Zhang et al., 2019a) and myeloid leukemia (De Marchi et al., 2019a).

### Autophagy

Autophagy is an important mechanism that maintains cell homeostasis by eliminating damaged cellular components and recycling them *via* metabolic pathways. By doing so, autophagy safeguards energy production under cellular stress such as starvation (White et al., 2015). Depending on the time of activation, autophagy can either help to prevent cancer or exert a tumor-promoting action (Mulcahy Levy and Thorburn, 2020). While data supporting P2X7 involvement in autophagy and cancer progression are limited, the receptor is known to act on several pathways related to autophagy including the pathway leading to autophagosome formation (Orioli et al., 2017). Indeed, P2X7 was shown to activate autophagosome-lysosome fusion by upregulating Beclin1 (Sun et al., 2015; Mawatwal et al., 2017) and LC3-II (Young et al., 2015; Fabbrizio et al., 2017; Mawatwal et al., 2017). In dystrophic myoblasts, P2X7-dependent activation of autophagy correlated with the ability to form the macropore but not calcium influx-induced signaling (Young et al., 2015). The first demonstrations of P2X7-mediated trophic activity were obtained by growing cells under serum starvation where autophagy is typically induced (Baricordi et al., 1999; Adinolfi et al., 2005). Cancer cells cultured under these conditions were found to upregulate P2X7 expression suggesting that autophagy can drive P2X7 overexpression and consequent trophic activity (Gomez-Villafuertes et al., 2015).

Chemotherapy-induced autophagy can be central in the modulation of the anticancer immune response (Kang et al., 2020). In particular, autophagy is a key driver in immunogenic cell death (ICD), a form of cancer cell death, which can elicit a potent immune response mediated by dendritic cells (DCs) and CD8^+^ T lymphocytes that can eradicate residual tumor cells and prevents metastasis formation following a first therapeutic intervention (Galluzzi et al., 2017). Several classes of chemotherapeutics such as anthracyclines and oxaliplatin, are known to cause the induction of ICD (Galluzzi et al., 2017). Before ICD takes place, premortem autophagy is required for the release of ATP in the TME where ATP attracts antigen-presenting cells *via* P2Y2 receptors (Michaud et al., 2011; Ma et al., 2013) and down-regulates Treg infiltration (Pietrocola et al., 2016), to favor an antitumor immune response. Although a clear correlation between P2X7 activation and ICD in the TME is not available, it was recently demonstrated that P2X7 expression modulates ATP levels in the TME affecting CD73 and CD39 ectonucleotidase and reducing Treg levels and fitness (De Marchi et al., 2019). P2X7 systemic blockade activated an ICD–like mechanism in cancer cells leading to tumor growth reduction and activation of antitumor responses while reducing the tumor-promoting inflammatory cytokine IL-1β (De Marchi et al., 2019).

### Role of P2X7 in the Immune Response

Analysis of data from P2X7 small molecule inhibitors and genetic knock out of *P2RX7* has demonstrated the key role played by P2X7 in inflammation and immunity (Di Virgilio et al., 2017; Adinolfi et al., 2018; Adinolfi et al., 2019) and, as a consequence, in chronic inflammatory diseases such as Duchenne muscular dystrophy (Ferrari et al., 1994; Sinadinos et al., 2015), rheumatoid arthritis (Stock et al., 2012) and inflammatory bowel disease (Arulkumaran et al., 2011; Eser et al., 2015). In the TME, the permanent release of DAMPs including ATP, high mobility group box 1 (HMGB1) and calreticulin (CRT) drives chronic inflammatory settings that are supplemented by immunosuppressive signals leading to a dampening of immune effector cell responses (Galluzzi et al., 2017). By responding to ATP proinflammatory signals, P2X7 counterbalances the potent immunosuppressive signaling driven by the ATP degradation product, adenosine (Li et al., 2019). In the following section, we describe the role of P2X7 in inflammation and immunity and use this as a foundation to define the role of P2X7 in the TME.

ATP, released through pannexin-1 hemichannels leads to activation of P2X7 as well as other P2 receptors including P2X1, P2X4, P2X5 and P2Y6 and is associated with the activation of T-cells (Wang et al., 2004; Overes et al., 2008; Schenk et al., 2008; Tsukimoto et al., 2009; Woehrle et al., 2010). However, the role of P2X7 in response to extracellular ATP appears to be central to the proinflammatory response including the stimulation of CD4^+^ and CD8^+^ effector T cells (Aswad et al., 2005; Schenk et al., 2008), stimulation of natural killer T cells (Beldi et al., 2008), inhibition of differentiation in type 1 regulatory cells, promotion of Treg cell death (Aswad et al., 2005; Schenk et al., 2011; Figliuolo et al., 2017) and the differentiation of inflammatory Th17 lymphocytes (Atarashi et al., 2008; Pandolfi et al., 2016). P2X7 was also found to collaborate with P2Y2 to promote the chemotaxis of myeloid cells including macrophages, neutrophils and the recruitment and activation of DCs (Idzko et al., 2002; Pelegrin et al., 2008; Savio et al., 2017). P2X7 activation also promotes the immunosuppressive role of myeloid-derived suppressor cells (MDSCs) infiltrated in the TME by stimulating the release of reactive oxygen species (ROS), arginase 1 (ARG1) and transforming growth factor-β1 (TGF-β1) (Bianchi et al., 2014).

At the molecular level, P2X7 plays a central role in the assembly and maturation of the NLRP3 inflammasome leading to the activation of caspase 1 followed by the cleavage of pro-interleukin 1 beta (pro-IL-1β) into mature IL-1β before IL-1β is released by macrophages and other immune cells (Ferrari et al., 1997b). Direct interaction of P2X7 with the components of the inflammasome including the apoptosis-associated speck-like protein containing a caspase recruitment domain (ASC) and the NOD-like receptor family (NLR) has been shown in neurons, astrocytes and microglial cells (Silverman et al., 2009; Minkiewicz et al., 2013; Franceschini et al., 2015). However, following opening of the P2X7 ion channel in response to the binding of ATP, it is the depletion of cytoplasmic K^+^ that is the main driver leading to inflammasome activation (Sanz and Di Virgilio, 2000; Munoz-Planillo et al., 2013). While other channels also mediate K^+^ efflux in response to ATP (Di et al., 2018), P2X7 activated K^+^ efflux together with Ca^2+^ influx remain central to inflammasome activation and the secretion of cytokines and chemokines. Among these, IL-1β is a key proinflammatory cytokine secreted in response to P2X7 activation of the inflammasome (Perregaux and Gabel, 1998; Perregaux et al., 2000; Solle et al., 2001). Other cytokines and chemokines are also secreted by a variety of cell types. These include IL-6 (Solini et al., 1999; Kurashima et al., 2012; Shieh et al., 2014), IL-8 (Wei et al., 2008), IL-18 (Perregaux et al., 2000), tumor necrosis factor alpha (TNFα) (Ferrari et al., 2000; Shieh et al., 2014), CC-chemokine ligand 2 (CCL2 also named MCP-1) (Panenka et al., 2001; Kurashima et al., 2012; Shieh et al., 2014), CCL3 (Kataoka et al., 2009), CCL7 (Kurashima et al., 2012), CXCL2 (Shiratori et al., 2010; Kurashima et al., 2012), and prostaglandin E2 (PGE2) (Panupinthu et al., 2008; Barbera-Cremades et al., 2012). P2X7 also triggers activation and shedding of matrix metalloproteases (MMPs) from peripheral-blood mononuclear cells (PBMCs) (Gu et al., 1998; Gu and Wiley, 2006; Young et al., 2018). It also has been suggested that P2X7 plays a role in the resolution of inflammation through secretion of the anti-inflammatory cytokines IL-10 and TGFβ and proteins such as annexin A1 (Chessell et al., 2005; Bianchi et al., 2014; Moncao-Ribeiro et al., 2014; de Torre-Minguela et al., 2016). However, other reports have demonstrated P2X7 mediated inhibition of soluble HLA-G and IL-10 secretion by monocytes (Rizzo et al., 2009) and the negative modulation of HLA-G in women affected with herpes virus 6 A (Pegoraro et al., 2020).

P2X7 may cooperate in mediating the response to pathogen-associated molecular patterns (PAMPs) such as lipopolysaccharides (LPS) through a potential LPS binding motif in the P2X7 C-terminal tail (Denlinger et al., 2001). LPS activation of TLR receptor also stimulates the production of pro-IL-1β, which is then processed by caspase-1 following P2X7 and NLRP3 inflammasome activation (Walev et al., 1995). Similarly, TLR2 and 4 were found to cooperate with P2X4 and P2X7 when activated by biglycan, a ubiquitous leucine-rich repeat proteoglycan found in the extracellular matrix (Babelova et al., 2009). P2X7 was shown to interact with the myeloid differentiation primary response 88 (MyD88) to activate the NF-kB signaling pathway (Liu et al., 2011). MyD88 mediates NF-kB signaling downstream of the TLR receptors and might mediate the cooperation between P2X7 and TLR signaling pathways. P2X7 together with CD14 as a coreceptor was found to support LPS binding and the internalization of P2X7 (Dagvadorj et al., 2015). Overall, these studies support a model whereby activation of TLR2 and 4 cooperate with P2X7 to drive chronic inflammatory settings. While ATP, PAMPs and biglycan were found to be key ligands of P2X7 and TLR receptors, in the TME, DAMPs such as ATP and HMGB1, a ligand for TLR4, are also present in significant concentrations and capable of driving the chronic inflammatory settings described above (Galluzzi et al., 2017).

### Role of the P2X7 in Immunometabolism

Metabolic reprogramming and the maintenance of mitochondrial fitness are increasingly recognized as key factors in T lymphocyte differentiation and effector functions (Borges da Silva et al., 2018; Bailis et al., 2019). Generation of long-lived T memory cells depends on efficient oxidative phosphorylation and fatty acid oxidation, while effector T cells are mainly dependent on glycolysis (Patel and Powell, 2017). Therefore, mitochondria have a central role in directing T cell functions. The receptors and pathways mediating the regulation of mitochondrial metabolism modulation in T cells are still poorly characterized, however it is increasingly clear that the P2X7 receptor plays an important role. P2X7 has long been known as a prototypic cytotoxic receptor (Di Virgilio et al., 1998), but its tonic activity also supports healthy mitochondrial metabolism, enhances ATP production *via* the respiratory chain and promotes cell growth (Adinolfi et al., 2005; Adinolfi et al., 2009). The specific role of P2X7 in supporting metabolic fitness of long-lived T memory cells has now been demonstrated (Borges da Silva et al., 2018). Stimulation of the P2X7 receptor *via* extracellular ATP is crucial to promote memory CD8^+^ T cell generation and long-term survival that is mediated mainly by a trophic effect on mitochondrial metabolism and the related increase in ATP synthesis. These data support a previous report from Di Virgilio and coworkers showing that P2X7 receptor expression has a broad effect on immunometabolism through modulation of glycolysis (Amoroso et al., 2012). Indeed, aerobic glycolysis is also impaired in P2X7-deficient CD8^+^ T lymphocytes (Borges da Silva et al., 2018). Thus, the P2X7 receptor may function as a “metabolic sensor” that links the DAMP extracellular ATP, to the intracellular energy-producing machinery (i.e., oxidative phosphorylation and glycolysis).

The “metabolic sensor” function of the P2X7 receptor is of particular relevance at inflammatory and tumor sites since the extracellular ATP concentration in the inflammatory or tumor microenvironments (IME and TME, respectively) is several-fold higher than in the healthy interstitium (Pellegatti et al., 2008; Di Virgilio et al., 2018a). The impact of such a high extracellular ATP concentration on the viability of tumor infiltrated lymphocytes due to opening of the macropore is not known. While it is possible that loss of viability may take place, the long-term presence of infiltrated lymphocytes suggests that modulation of abrogation of macropore function through one or more of the mechanisms described in **Table 1** is likely to take place in these cells. Although details of the mechanism whereby P2X7 ‘transduces’ the information carried by high extracellular ATP is not fully known, key immune functions are significantly affected by P2X7 activation (Di Virgilio et al., 2017; Di Virgilio and Adinolfi, 2017). Most importantly, P2X7 is a major stimulus for Il-1β and IL-18 release, and therefore for the initiation of inflammation, potentiation of antigen presentation by DCs and an efficient immune response, including antitumor immune responses (Mutini et al., 1999; Di Virgilio et al., 2018a). Converging reports by multiple laboratories have confirmed the initial observation by Di Virgilio and coworkers of a potent P2X7-mediated growth-promoting and stimulatory effect on human T lymphocytes (Baricordi et al., 1996; Schenk et al., 2008; Yip et al., 2009; Yu et al., 2010; Danquah et al., 2016). P2X7 is upregulated in Treg and T follicular helper cells (Tfh) that are severely inhibited by its activation (Gavin et al., 2007; Taylor et al., 2008; Hale et al., 2013). This is hypothesized to have important implications for host-microbiota interaction in gut-associated secondary lymphoid organs where ATP released by bacteria down-modulates the activity of resident Tfh cells, thus reducing secretion of high affinity IgA and their binding to commensal bacteria (Di Virgilio et al., 2018c). Grassi and coworkers have shown that P2X7 deletion affects microbiota composition and, in consequence, host metabolic homeostasis (Perruzza et al., 2017; Proietti et al., 2019). This might be significant in view of the known role of microbiota in the pathogenesis of several types of cancer and the influence of microbiota on the response to immune checkpoint inhibitors (Gopalakrishnan et al., 2018; Zitvogel et al., 2018; Vivarelli et al., 2019).

### P2X7 and Purinergic Signaling in Antitumor Immunity

The function of the P2X7 receptor in the TME is better understood in the context of the overall mechanisms that control the extracellular ATP concentration (Adinolfi et al., 2019). Of importance is the role of CD39 and CD73 ectonucleotidases, that hydrolyze ATP to adenosine (Vijayan et al., 2017). Adenosine has long been considered one of the main drivers of immunosuppression (Antonioli et al., 2013). Yet, recent data demonstrates that the P2X7, CD39, and CD73 axis also plays a significant role in the modulation of the TME immune component. P2X7 activation can be promoted by targeting CD39, with beneficial effects on antitumor responses that cannot be explained merely by the inhibition of adenosine generation (Li et al., 2019). The use of P2X7 targeted small molecule inhibitors and *P2RX7* genetic knock out in a murine melanoma model leads to modulation of CD39 and CD73 expression levels on several immune cell populations including Tregs, CD4^+^ effector lymphocytes, macrophage and DCs (De Marchi et al., 2019). P2X7 null mice show a decrease in tumor-infiltrated CD8^+^ T cells and an increase in Tregs overexpressing the fitness markers PD-1, OX40, and CD73. This outcome correlated with a decrease of extracellular ATP levels. In contrast, systemic inhibition of P2X7 with the antagonist A740003 did not affect the number of tumor-infiltrated CD8^+^ and Treg lymphocytes but increased the number of CD4^+^ effector T cells. A reduced expression of CD39 and CD73 was also observed on CD4^+^ T cells and in DCs highlighting the importance of the crosstalk between P2X7, CD39, and CD73 in these cell types (De Marchi et al., 2019). The collaboration between P2X7 and CD39 was further demonstrated in two recent studies investigating the antitumoral activity of a CD39 blocking antibody (Li et al., 2019; Yan et al., 2020). Blocking of CD39 led to reduced tumor burden and metastatic spread in several murine models. Anti-CD39 antitumoral activity required the activation of P2X7 on immune cells as P2X7-mediated activation of the NLRP3 inflammasome led to IL-18 release by myeloid cells and induction of pyroptosis (Li et al., 2019; Yan et al., 2020). These data demonstrate that P2X7 collaboration with CD39 plays a central role in the regulation of anti-tumor immunity.

### Membrane Blebbing

Membrane blebbing consists of plasma membrane sections protruding and retracting to create protrusions at the cell surface. Although the mechanism leading to membrane blebbing is not fully understood, P2X7 is one of the proteins that have been shown to initiate this mechanism upon sustained ATP stimulation for several minutes (MacKenzie et al., 2001). ATP mediated opening of the P2X7 macropore leads to a large Ca^2+^ influx that can induce membrane blebbing. Multiple proteins are involved in this mechanism. These include the serine/threonine kinase ROCK I (Morelli et al., 2003), heat shock protein HSP90 that negatively regulates receptor-mediated blebbing (Adinolfi et al., 2003) and the epithelial membrane protein 2 (EMP-2) (Wilson et al., 2002). EMP-2 was shown to interact with the P2X7 C-terminal tail suggesting that P2X7 mediated blebbing requires opening of the macropore (Wilson et al., 2002). While membrane blebbing is commonly associated with cellular stress and the initiation of the apoptotic pathway, P2X7 may also be involved in triggering reversible membrane blebbing allowing leukocytes to migrate and invade through extracellular matrices (Qu and Dubyak, 2009). Finally, P2X7-mediated membrane blebbing has been proposed as an early step leading to shedding of microvesicles in the microenvironment (Qu and Dubyak, 2009).

### Release of Microvesicles

Since the discovery that P2X7 is central to the release of the proinflammatory cytokine IL-1β by macrophages, there have been extensive investigations on how this mechanism occurs, since IL-1β belongs to the leaderless secretory protein group lacking the secretory signal necessary for conventional protein secretion through the endoplasmic reticulum route (Dinarello, 2002). These studies have shown that IL-1β secretion is instead mainly caused by P2X7 induced nonconventional pathways including microvesicle shedding, exosomes and modified lysosomes (MacKenzie et al., 2001; Ferrari et al., 2006; Pizzirani et al., 2007; Qu et al., 2007; Lopez-Castejon and Brough, 2011; Piccioli and Rubartelli, 2013). Other studies further showed involvement of P2X7 in microvesicles that release cytokines by fibroblasts and microglia (Solini et al., 1999; Bianco et al., 2005), HIV particles by macrophages (Graziano et al., 2015) and tissue factor by macrophages and DCs (Baroni et al., 2007; Moore and MacKenzie, 2007).

### Release of ROS

During environmental stress such as ionizing radiation, UV, or heat exposure, increased ROS production mediates oxidative stress, damages cellular structures and initiates cell death. However, low levels of ROS can act as signaling molecules. In phagocytes, ROS are used as bactericides to complete the phagocytosis process (D’Autreaux and Toledano, 2007; Reczek and Chandel, 2015). Hence, cells need to control ROS levels tightly in order to meet their physiological needs, while preventing cell death (Dupre-Crochet et al., 2013). Production of ROS is mediated mainly by NADPH oxidases (NOXs), and the electron transport chain in mitochondria. During tumor development, oncogenic stimulation, increased metabolic activity and mitochondrial defects lead to increased ROS production forcing cancer cells to increase their antioxidant capacity to prevent cell death (Reczek and Chandel, 2015). Several reports have shown the induction of ROS production downstream of P2X7 activation [reviewed in (Guerra et al., 2007)]. They show that P2X7-mediated Ca^2+^ influx leads to activation of kinases that phosphorylate NADPH oxidases (NOXs) to activate the production of ROS (Moore and MacKenzie, 2009; Wang and Sluyter, 2013). However, there are contradictory reports on the nature of the pathway involved in the phosphorylation of NOXs. In microglial cells, p38 MAPK and PI3K but not ERK1/2 have been shown to mediate ROS production (Parvathenani et al., 2003). In macrophages, two studies have shown activation of ERK1/2 downstream of PKC, c-Src, Pyk2 but no involvement of PI3K or p38 MAPK (Lenertz et al., 2009; Martel-Gallegos et al., 2013) while Noguchi et al. reported activation of the ASK1, p38 MAPK signaling pathway is required for ROS production (Noguchi et al., 2008). Low levels of ATP stimulation increase mitochondrial Ca^2+^ content leading to hyperpolarization of mitochondrial potential and increased ATP production (Adinolfi et al., 2005). In line with these data, P2X7 was shown to drive the expression of glycolytic enzymes leading to increased glycolysis and oxidative phosphorylation that sustains cancer cell growth in the absence of glucose and serum (Amoroso et al., 2012).

## Control of P2X7 Localization as a Mean to Modulate P2X7 Function

P2X7 has been shown to localize in several cellular compartments including the plasma membrane, endoplasmic reticulum (ER), lysosomes, and phagosomes (Kuehnel et al., 2009; Robinson and Murrell-Lagnado, 2013). In human monocytes and lymphocytes, P2X7 was found to be localized mainly intracellularly while differentiation of monocytes into macrophages led to P2X7 localizing to the plasma membrane (Hickman et al., 1994; Gu et al., 2000; Gudipaty et al., 2001). In contrast, in mouse microglia and macrophages, P2X7 localizes mainly at the plasma membrane (Boumechache et al., 2009). In cancer cells, Gilbert and colleagues showed that nfP2X7 colocalizes with the ER marker, calreticulin indicating that a misfolded form of the receptor may be retained intracellularly. This was further supported by the relocalization of this form to the plasma membrane upon treatment with small molecule corrector KM11060 that increases the rate of protein secretion (Gilbert et al., 2019). Interestingly, this increased membrane expression was also stimulated by high ATP concentrations (500 µM and above) that suggests that cells may be using ER to plasma membrane localization as a mean to regulate P2X7 functions (Gilbert et al., 2019).

Palmitoylation of cysteine residues at the hinge of the C-terminal tail was shown to be required for P2X7 to localize to the plasma membrane and associate with lipid rafts (Gonnord et al., 2009). While several reports have confirmed P2X7 interaction with lipid rafts in multiple cell types (Bannas et al., 2005; Garcia-Marcos et al., 2006; Barth et al., 2007), the localization of P2X7 in lipid rafts may not be exclusive. Garcia-Marcos et al. have identified two P2X7 pools located in both lipid raft and nonlipid raft membrane compartments (Garcia-Marcos et al., 2006). They further showed that the nonlipid raft P2X7 pool can open a nonselective cation channel while the lipid raft P2X7 pool only is able to activate phospholipase A2 (PLA_2_) (Garcia-Marcos et al., 2006). Hence, the nature of the lipid content in the membrane surrounding P2X7 receptors can regulate receptor signaling and ultimately its function. Indeed, cholesterol was shown to inhibit P2X7 macropore function (Robinson et al., 2014; Karasawa et al., 2017) while sphingomyelin and 1-palmitoyl-2-oleoyl-sn-glycero-3-phospho-(1’-rac-glycerol) (POPG) were shown to enhance macropore function (Karasawa et al., 2017). Similarly, phosphatidylinositol 4,5-bisphosphate (PIP2) was shown to modulate P2X7 ion channel function (Zhao et al., 2007). Although no direct interaction was found between P2X7 and PIP2, several residues in the C-terminal tail (R385, K387 and K395) were shown to be involved in PIP2 regulation. The I568N polymorphism also located in the P2X7 C-terminal tail was shown to impair P2X7 trafficking to the membrane (Wiley et al., 2003) while the H155Y polymorphism increased P2X7 localization to the plasma membrane (Bradley et al., 2011).

Evidence from several groups points to the existence of signaling cassettes located in the C-terminal tail of P2X7 which supports P2X7 trafficking to the plasma membrane (Denlinger et al., 2003; Wiley et al., 2003; Smart et al., 2003; Chaumont et al., 2004). Indeed, Lenertz have shown the presence of an ER retention/retrieval cassette in the P2X7 C-terminal tail near R576 (Lenertz et al., 2009). This cassette allows P2X7 monomers to be retained in the ER while being assembled into trimers. P2X7 channels are then addressed to the plasma membrane through the secretory pathway (Lenertz et al., 2009). A similar mechanism has already been reported for other multimeric membrane receptors such as the N-methyl-D-aspartate (NMDA), glutamate receptor and the γ-aminobutyric acid type B (GABAB) receptor (Michelsen et al., 2005). The section of P2X7 C-terminal tail overlapping with that cassette also was found to bind to phospholipids as well as LPS in a macrophage cell line thereby modulating receptor membrane localization and its capacity to signal through the MAP Kinase and NF-κB pathway (Denlinger et al., 2001; Liu et al., 2011).

### The Function and Expression of P2X7 Across Cancer Types

The presence of P2X7 has been shown in a large number of studies using tumor-derived biopsies, cell lines, xenografts and syngenic murine models across multiple and diverse cancer types. The majority of these publications, which demonstrate the presence of P2X7, have not directly assessed functionality of the P2X7 macropore. Given the high concentration of ATP in solid tumors, present at levels capable of inducing P2X7 pore activation (Pellegatti et al., 2008) and thereby death, it is unlikely that cells expressing fully functional P2X7 could survive in the TME over extended time periods. Therefore, it is probable that cells expressing P2X7 within the TME need to express P2X7 predominantly in a form where pore activity is attenuated. Despite this attenuation of pore function, it is clear from a number of studies that the P2X7 expressed in these cancer cells retains significant signaling functionality and the ability to drive the formation, survival and metastatic potential of tumor cells as previously reported (Jelassi et al., 2011; Adinolfi et al., 2012a; Amoroso et al., 2015; De Marchi et al., 2019; Gilbert et al., 2019).

The P2X7 receptor is expressed on a number of cancer types including, but not limited to prostate, lung, kidney, colorectal, gastric, breast, cutaneous squamous-cell and basal-cell carcinomas, melanoma, leukemia, neuroblastoma, glioma, ovarian, cervical, bladder, papillary thyroid, pancreatic and bone cancer. This includes nfP2X7 (Barden et al., 2014; Gilbert et al., 2019). The key data demonstrating the expression and function of P2X7 on these cancer types is discussed below while **Table 2** provides a list of references per cancer type.

#### Prostate Cancer

In an immunohistochemistry (IHC) study looking at 116 prostate cancer biopsies using an affinity-purified polyclonal antibody to the E200 epitope of P2X7 (supplied by Biosceptre), P2X7, in a nonpore functioning form, was identified in all malignant samples regardless of their stage or the age of the patient (Slater et al., 2004a). This was confirmed using a mouse monoclonal antibody (BPM09, supplied by Biosceptre) (Maianski et al., 2007). In another study, increased expression of nfP2X7 was observed as prostate disease progressed (Barden et al., 2016). P2X7 expression by IHC also was compared with the levels of prostate-specific antigen (PSA) in 174 prostate cancer biopsies (Slater et al., 2005). Increased nfP2X7 staining correlated with increased PSA levels indicating that P2X7 may provide a diagnostic biomarker candidate for early prostate cancer. These results were consistent with those of a separate study demonstrating increased P2X7 mRNA and protein expression in prostate tumor samples compared with normal tissue (Ravenna et al., 2009). Increased P2X7 expression correlates with expression of epidermal growth factor receptor (EGFR) and estrogen receptor (ER)α, which are well known drivers of cancer cell proliferation, suggesting that P2X7 might cooperate with these receptors to promote cell proliferation (Ravenna et al., 2009). These observations support the proposed role for P2X7 in cancer cell survival and proliferation (Adinolfi et al., 2009; Adinolfi et al., 2010). Indeed, functional P2X7 was shown to drive invasion and metastasis of prostate cancer cell lines stimulated by extracellular ATP (Ghalali et al., 2014; Qiu et al., 2014).

#### Lung Cancer

In a study analyzing P2X7 mRNA expression in 26 patients with nonsmall cell lung cancer (NSCLC), compared with 21 patients with chronic obstructive pulmonary disease (COPD) without signs of malignancy, higher P2X7 expression was observed in bronchoalveolar lavage derived cells of tumors with distant metastases (Schmid et al., 2015). P2X7 is also expressed in human NSCLC cell lines including A549, PC9 and H292 cells but not in the nonmalignant bronchial epithelial cells BEAS-2B (Barden et al., 2009; Takai et al., 2012; Jelassi et al., 2013; Takai et al., 2014). In H292 cells, inhibition or down-regulation of P2X7 abrogated TGF-β1 induced migration and actin remodeling. P2X7 is required for TGF-β1-induced exocytosis of ATP that then acts as a paracrine factor in lung cancer cell model. Overall, these data suggest a role for P2X7 in promoting invasion and the development of aggressive forms of lung cancer.

#### Kidney Cancer

Clear-cell renal cell carcinoma (ccRCC) is the most common form of renal cell carcinoma. In a study analyzing 273 ccRCC patients by IHC, P2X7 expression was correlated with the clinicopathologic features and cancer-specific survival (CSS) (Liu et al., 2015). Although intratumoral P2X7 expression was lower than peritumoral P2X7 expression, those patients with high intratumoral P2X7 expression had a worse prognosis. Overall, these data suggest that intratumoral P2X7 is involved in the progression of ccRCC. In contrast, the significant P2X7 expression found in peritumoral tissues may reflect P2X7 involvement in the TME. Further exploration of the cell type expressing P2X7 in ccRCC TME is now needed to better understand the nature of that involvement.

#### Colorectal and Gastric Cancer

P2X7 protein was identified by IHC with staining distributed throughout the cell in a small number of normal colorectal epithelia and colon adenocarcinomas (Li et al., 2009). Qian et al. demonstrated that high P2X7 expression correlated with tumor size, lymph nodes metastasis, TNM stage and was also associated with poor overall survival in a cohort of 116 colon carcinoma (Qian et al., 2017). These contradicting results suggest that there may be subtypes of colorectal cancer characterized by their level of P2X7 expression. Indeed, Zhang et al. analyzed normal tissue and colorectal cancer samples from 97 patients and found both P2X7 high and P2X7 low populations with P2X7 high population having increased metastasis and reduced survival (Zhang et al., 2019b). Both P2X7 and nfP2X7 were identified in human colorectal cancer epithelium although the nfP2X7 expression was much more significant (Barden et al., 2014; Gilbert et al., 2019). While nfP2X7 was identified in the human HT-29 and Colo-205 colon cancer cell lines (Barden et al., 2009), other reports have shown P2X7 expression in human HCT8, Caco-2, Colo-205, and murine MCA38 colon cancer cell lines and the latter cell line was found to possess functional P2X7 macropore (Coutinho-Silva et al., 2005; Kunzli et al., 2011; Bian et al., 2013). Analysis of P2X7 expression in 156 gastric cancers corelated P2X7 expression with tumor burden and poor survival suggesting that P2X7 may be involved in the progression of gastric cancer (Calik et al., 2020).

#### Breast Cancer

An IHC study analyzing nfP2X7 expression in 40 breast tumors of diverse histological subtypes demonstrated that nfP2X7 expression was absent in normal and hyperplastic breast epithelial samples while *in situ* or invasive lobular or ductal carcinoma expressed high levels of nfP2X7 (Slater et al., 2004b). Tumor cells from invasive carcinomas showed membrane staining as opposed to intracellular staining in *in situ* carcinomas. Therefore, nfP2X7 membrane staining may be stage-specific and nfP2X7 expression levels may help to distinguish between different stages of breast cancer. P2X7 mRNA and protein also were upregulated under hypoxic conditions in the noninvasive breast cancer cell line MCF-7 which has a nonfunctional P2X7 pore (Tafani et al., 2010; Chadet et al., 2014). In contrast, two independent studies looking at P2X7 expression showed a reduction of P2X7 staining in breast cancer versus normal tissue (Li et al., 2009; Huang et al., 2013). The antibodies used in these studies were raised against the C-terminal tail of the receptor and are therefore likely to mainly detect P2X7 variant A. These antibodies are unable to discriminate between functional P2X7 and nfP2X7. Hence, while discrepancies exist for the expression of P2X7 variant A in breast cancer, nfP2X7 appears to be specifically upregulated at the surface of breast cancer cells. Tan et al. reported higher P2X7 expression levels in the breast cancer tissues when compared with normal breast tissue (Tan et al., 2015). Furthermore, a positive correlation was observed between P2X7 expression and the estrogen receptor (ER)+ by qRT-PCR, western blot and immunohistochemistry analysis.

#### Cutaneous Squamous-Cell and Basal-Cell Carcinomas

P2X7 is highly expressed both in nodular basal cell carcinomas (BCC) and in infiltrative BCC cells where it was shown to be present in some tumor cell nuclei (Greig et al., 2003). P2X7 expression was also found in the human squamous cell carcinoma (SCC) cell line A431, (Greig et al., 2003). nfP2X7 protein also was upregulated in 25 SCC compared to 20 keratoacanthomas (KA) (Slater and Barden, 2005). Using an affinity purified sheep polyclonal antibody to nfP2X7, systemic treatment of advanced cat SCC showed decreased lesion size (Barden et al., 2016). In a more recent study, the presence of nfP2X7 was shown in BCC and antibodies specific for nfP2X7 rather than fully functional P2X7 were shown to be a safe treatment for BCC with 65% of patients showing reduced lesion area, 20% showing stable tumor size and 15% with increased lesion area (Gilbert et al., 2017). Overall, these data support the targeting of nfP2X7 in BCC and SCC.

#### Melanoma

In an IHC study looking at 80 human melanoma biopsies, nfP2X7 protein was overexpressed in malignant tissues when compared with nonmalignant samples. P2X7 upregulation was also observed on keratinocytes of the epidermis surrounding the tumor (Slater et al., 2003). A study analyzing P2X7 in 14 human melanoma biopsies confirmed the upregulation of P2X7 in melanoma with over 75% of samples staining positively (White et al., 2005). Upregulation of P2X7 in melanoma samples was confirmed in cell lines at the mRNA level. Indeed, whole genome microarray screening of the NCI-60 cancer cell line panel has shown full length P2X7 upregulation as a hallmark of melanoma cell lines (Shankavaram et al., 2009; Reinhold et al., 2012; Roger et al., 2015). The human melanoma cell line A375 was found to express P2X7 variant A and to have a functional large pore (White et al., 2005).

#### Leukemia

P2X7 is upregulated in T-cell acute lymphoblastic leukemia and in murine erythroleukemia (MEL) cells (Constantinescu et al., 2010; Chen et al., 2014). In MEL cells, P2X7 was also shown to have a functional macropore (Constantinescu et al., 2010). P2X7 was also upregulated in pediatric leukemias (Chong et al., 2010a) as well as in human myeloid leukaemic cell lines F-36P and HL-60 (Yoon et al., 2006). In a separate study, P2X7 mRNA and protein were upregulated in 8 out of 11 cell lines, 69 out of 87 bone marrow mononuclear cell (BMMC) samples from leukemia patients and 9 out of 10 myelodysplastic syndrome (MDS) patients (Zhang et al., 2004). Furthermore, P2X7 also was significantly upregulated in acute myelogenous leukemia (AML) and acute lymphoblastic leukemia (ALL) (Zhang et al., 2004). P2X7 expression was found to be higher in AML subtypes having poor prognosis. Following standard induction, P2X7 was upregulated in a group with a low remission rate (Zhang et al., 2004). Increased P2X7 expression was found in lymphocytes from patients with the evolutive form of B-cell chronic lymphocytic leukemia (B-CLL) (Adinolfi et al., 2002). Furthermore, P2X7 expression correlated with the severity of B-CLL. However, the large molecular weight pore function was not assessed in this study (Adinolfi et al., 2002). In a separate study, antibodies targeting P2X7 were shown to bind to the surface of B-CLL cells. While the analysis of P2X7 membrane expression by flow cytometry suggests that the B-CLL populations tested are not fully uniform, data suggest that B-CLL populations with both functional and nonfunctional macropore can be identified (Gu et al., 2000). Overall, these data support a role for P2X7 in the progression of leukemia and the development of aggressive forms of the disease.

#### Neuroblastoma

In an IHC study, P2X7 was highly expressed in neuroblastoma irrespective of the tumor grade. P2X7 was also expressed in several neuroblastoma cell lines including ACN, GI-CA-N, HTLA-230, GI-ME-N, LAN-5, LAN-1, SK-N-BE-2, and SH-SY-5Y with cell surface staining characterized for at least ACN (Raffaghello et al., 2006). P2X7 stimulation *in vitro* with ATP did not induce apoptosis of neuroblastoma cells but instead stimulated their proliferation *via* the enhanced secretion of substance P, which suggests that these cells were nonfunctional for P2X7 macropore (Raffaghello et al., 2006). In an independent study, P2X7 mRNA expression was analyzed in 131 patients with neuroblastoma (Amoroso et al., 2015). High P2X7 expression correlated with poor prognosis while patients with low P2X7 expression levels had less aggressive tumors (Amoroso et al., 2015).

#### Glioma

Both mRNA and protein were detected in human glioma cell lines U-138MG, U-251MG, and M059J. P2X7 was shown to mediate cell death in response to ATP in glioma cells (Wei et al., 2008; Tamajusuku et al., 2010; Gehring et al., 2012; Fang et al., 2013). While U-138 MG and U-251 MG cells appeared to have a nonfunctional P2X7 pore, M059J glioma cells were reported as having functional receptor macropore (Gehring et al., 2012). P2X7 is also upregulated in mouse GL261 glioma cells (Bergamin et al., 2019). P2X7 was also upregulated in an *in vivo* model where rats received an intra-striatal injection of C6 glioma cells (Ryu et al., 2011). In these cells, P2X7 promoted chemotaxis *in vitro,* which suggests that P2X7 may play a role in the metastasis process of glioma (Ryu et al., 2011). In a separate study, Gehring et al. showed that high P2X7 expression was a good prognostic factor for glioma radiosensitivity and survival probability (Gehring et al., 2015). P2X7 was highly upregulated at the mRNA and protein level in tumor tissue as opposed to peritumoral and adjacent normal tissue (Tafani et al., 2011). P2X7 upregulation was also observed in cancer stem cells from GBM cultured under hypoxic conditions. This evidence supports the potential involvement of P2X7 in cancer stem cells (Tafani et al., 2011).

#### Ovarian Cancer

P2X7 expression was analyzed by IHC in nine human ovarian carcinoma biopsies and compared with ovarian surface epithelium in healthy tissue (Vazquez-Cuevas et al., 2014). P2X7 was highly expressed in both healthy tissue and ovarian carcinoma (Vazquez-Cuevas et al., 2014). P2X7 also was found to be expressed in the human ovarian cancer cell lines SKOV-3 and CAOV-3 (Vazquez-Cuevas et al., 2014). Both P2X7 and nfP2X7 were expressed in ovarian cancer tissue (Barden et al., 2014; Gilbert et al., 2019).

#### Cervical Cancer

IHC analysis of squamous cell cancer of the cervix showed increased nfP2X7-specific membranous staining when compared to normal tissue (Barden et al., 2014). In contrast, Li et al. have reported a decreased expression of full length P2X7 in tissues of complex hyperplasia with atypia or endometrial adenocarcinoma compared to normal endometrium, simple hyperplasia or complex hyperplasia tissues (Li et al., 2007).

#### Bladder Cancer

Using Protein Pathway Array (PPA) to investigate the expression of 285 proteins and phosphoproteins in bladder urothelial cell carcinoma tissues and adjacent nontumor tissues, Hu et al. reported that expression of P2X7 was an independent factor, favorable for overall survival (Hu et al., 2016). Both P2X7 and nfP2X7 were confirmed to be expressed in bladder cancer tissue in separate IHC studies (Barden et al., 2014; Gilbert et al., 2019).

#### Papillary Thyroid Cancer

P2X7 mRNA was upregulated in 37 human papillary thyroid cancer (PTC) samples when compared with adjacent normal tissue (Solini et al., 2008). P2X7 protein upregulation was also confirmed by IHC in all malignant tissues tested including the classical and follicular forms of the disease (Solini et al., 2008). Adjacent normal tissue was devoid of P2X7 staining. P2X7 staining was found to be diffuse in the cytoplasm and intense at the cell surface of malignant thyrocytes (Solini et al., 2008). P2X7 mRNA and protein was also detected in FB1 and FB2 human thyroid cancer cell lines. In response to ATP, these cells showed a P2X7 dependent release of IL-6, a cytokine associated with aggressiveness of PTC (Ruggeri et al., 2002; Solini et al., 2008). P2X7 upregulation in PTC was confirmed by IHC in two independent studies (Li et al., 2009; Gu L. et al., 2010). P2X7 upregulation was shown to correlate with tumor growth, capsular infiltration and lymph node metastases (Gu L. et al., 2010; Kwon et al., 2014). Additional reports showed nfP2X7 expression in PTC tissue (Barden et al., 2009; Barden et al., 2014). Overall, these studies support a correlation between P2X7 and nfP2X7 expression and the development of aggressive PTC.

#### Pancreatic Cancer

P2X7 mRNA and protein was shown to be upregulated in chronic pancreatitis and pancreatic cancer tissue and cell lines *in vitro* and *in vivo* (Kunzli et al., 2007; Giannuzzo et al., 2015; Giannuzzo et al., 2016). P2X7 large pore functionality was not assessed in these studies. Other reports demonstrated both P2X7 and nfP2X7 expression in pancreatic cancer tissue (Barden et al., 2014; Gilbert et al., 2019).

#### Bone Cancer

P2X7 was upregulated in osteosarcoma, Ewing sarcoma, chondromyxoid fibroma as well as in bone cancer cell lines such as SaOs-2 and HOS (Gartland et al., 2001; Alqallaf et al., 2009; Liu and Chen, 2010). SaOs-2 cells have a functional P2X7 large pore (Alqallaf et al., 2009). Moreover, P2X7 upregulation plays a key role in several malignancies metastasising to the bone that supports an involvement of P2X7 in the host tissue during the metastatic process (reviewed in (Di Virgilio et al., 2009; Adinolfi et al., 2012b; Adinolfi et al., 2015a). P2X7B splice variant was upregulated in osteosarcoma and its expression was associated with cell proliferation (Giuliani et al., 2014).

## Therapeutic Approaches Taken to Target P2X7

Several pharmaceutical organizations have engaged in the development of both small-molecules and biologics directed against P2X7 (Mehta et al., 2014; Baudelet et al., 2015; Jacobson and Muller, 2016) (**Table 3**). These developments can be seen in the increasing number of filed patents for P2X7 targeted treatment (Gunosewoyo and Kassiou, 2010; Sluyter and Stokes, 2011; Park and Kim, 2017; Pevarello et al., 2017). Early development of small molecule inhibitors targeted the P2X7 orthosteric site to compete ATP mediated P2X7 activation. However, the lack of specificity of these inhibitors led to the identification and development of several classes of inhibitors that bind to the inter-subunit allosteric pocket preventing ATP induced rotation of each subunit and closure of the turret (Karasawa and Kawate, 2016). Within this inter-subunit allosteric pocket, several point mutants including but not limited to F88A, D92A, F95A, and F103A were identified to play an important role in the mode of action of these inhibitors (Allsopp et al., 2017; Allsopp et al., 2018; Bin Dayel et al., 2019). Engagement of this allosteric pocket allowed progressive development of antagonists in the low nanomolar range (**Table 2**) while providing better selectivity for P2X7 against other P2X family members (Donnelly-Roberts et al., 2009a; Allsopp et al., 2017; Allsopp et al., 2018; Bin Dayel et al., 2019). Initial identification of lead candidates against the inter-subunit allosteric pocket revealed compounds with good activity against human P2X7 but inactive against the rat isoform making pharmacology studies difficult (Michel et al., 2008a; Michel et al., 2008b; Caseley et al., 2015). However, docking of these candidates in the inter-subunit allosteric pocket highlighted the importance of human F95 (L95 in rat P2X7) in developing pi-stacking interactions with inhibitors (Caseley et al., 2015; Allsopp et al., 2017; Allsopp et al., 2018).

**Table 3:** Small molecule inhibitors and biologics used against P2X7.

Molecule	Format	pEC50/pIC50 (method used)	Binding site/other targets	Development stage	Disease settings	References
**Nonselective inhibitors**						
Suramin	Small molecule	> 300 μM (change in current)	Orthosteric site; Binds to multiple other targets including several P2 receptor subtypes, several growth factors, human immunodeficiency virus (HIV) reverse transcriptase, vasoactive intestinal peptide receptors and G protein–coupled receptors.	In the clinic	Used as antiparasitic agent against trypanosomes infection. Tested in the clinic for the treatment of autism and hand foot and mouth disease. Tested as combination with chemotherapy in multiple solid and blood born tumors.	(Middaugh et al., 1992; Surprenant et al., 1996; Jacobson et al., 2002)
NF279 (Suramin analog)	Small molecule	20 µM (Ca^2+^ influx at 3min) 891 nM (Yo-Pro uptake)	Orthosteric site High affinity to P2X_1_	Preclinical	N/A	(Donnelly-Roberts et al., 2009a)
MRS2159	Small molecule	1.7 µM (Ca^2+^ influx at 3min) 288 nM (Yo-Pro uptake)	High affinity to P2X_1_	Preclinical	N/A	(Donnelly-Roberts et al., 2009a)
2_,3_-dialdehyde ATP (oxidized ATP)	Small molecule closely related to ATP	100–300 μM (change in current)	Orthosteric site	Preclinical	N/A	(Surprenant et al., 1996; Michel et al., 2000; Hibell et al., 2001)
pyridoxalphosphate-6-azophenyl-2’,4’-disulfonic acid (PPADS)	Small molecule	3.2 µM (Ca^2+^ influx at 3min) 1.2 µM (Yo-Pro uptake)	Orthosteric site	Preclinical	N/A	(Donnelly-Roberts et al., 2009a; Huo et al., 2018)
pyridoxal-5′-phosphate-6-(2′-naphthylazo-6′-nitro-4′,8′-disulphonate) (PPNDS)	Small molecule	407 nM (Ca^2+^ influx at 3min) 200 nM (Yo-Pro uptake)	Orthosteric site	Preclinical	N/A	(Donnelly-Roberts et al., 2009a)
KN-62	Small molecule	10 µM (Ca^2+^ influx at 3min) 214 nM (Yo-Pro uptake)	Inter-subunit allosteric pocket and central cavity involving D92, F103, T90, and V312 in human P2X7. High concentrations (≥1 μM) also block Ca2^+^/calmodulin-dependent protein kinase II.	Preclinical	N/A	(Donnelly-Roberts et al., 2009a; Bin Dayel et al., 2019)
Brilliant Blue G (BBG)	Small molecule	< 100 µM (Ca^2+^ influx at 3min) 1.9 µM (Yo-Pro uptake)	Inter-subunit allosteric pocket and central cavity involving D92, F103, and V312 residues. Also blocks voltage-gated sodium channels.	Preclinical	N/A	(Donnelly-Roberts et al., 2009a; Bin Dayel et al., 2019)
**Selective inhibitors**						
A438079	Small molecule	123 nM (Ca^2+^ influx at 3min) 933 nM (Yo-Pro uptake)	Inter-subunit allosteric pocket involving F88, D92, T94, F95, and F103 residues.	Preclinical	N/A	(Nelson et al., 2006; Donnelly-Roberts et al., 2009a; Allsopp et al., 2018)
A740003	Small molecule	44 nM (Ca^2+^ influx at 3min) 93 nM (Yo-Pro uptake)	Inter-subunit allosteric pocket involving F88, D92, T94, F95 and F103, M105 and V312 residues.	Preclinical	N/A	(Honore et al., 2006; Donnelly-Roberts et al., 2009b; Karasawa and Kawate, 2016; Allsopp et al., 2018)
A804598	Small molecule	11 nM (Ca^2+^ influx)	Inter-subunit allosteric pocket involving F88, F95, F103, M105, and F108 residues.	Preclinical	N/A	(Donnelly-Roberts et al., 2009b; Karasawa and Kawate, 2016)
A839977	Small molecule	20–150 nM (Ca^2+^ influx and Yo-Pro uptake)	Not known	Preclinical	N/A	(Honore et al., 2009)
AZ10606120	Small molecule	10 - 200 nM (Ethudium uptake and Ca^2+^ influx)	Inter-subunit allosteric pocket involving residues 73 to 79, T90, T94, F103, and V312 residues.	Preclinical	N/A	(Michel and Fonfria, 2007; Karasawa and Kawate, 2016; Allsopp et al., 2017)
AZ11645373	Small molecule	10–20 nM (change in current and Yo-Pro uptake)	Inter-subunit allosteric pocket involving L83, S86, F88, D92, T94, F95, P96, F108, I310, and V312 residues.	Preclinical	N/A	(Stokes et al., 2006; Caseley et al., 2015; Bin Dayel et al., 2019)
AZD9056	Small molecule	12 nM (IL-1β secretion)	Not known	Evaluated in a Phase 2b against rheumatoid arthritis. Clinical trial number: NCT00520572	Rheumatoid arthritis	(Keystone et al., 2012; McInnes et al., 2014)
GSK1482160	Small molecule	Kd=5.09 ± 0.98 nmol/l, Ki=2.63 ± 0.6 nmol/l ([11C]GSK1482160 binding to HEK293-hP2X7 live cells)	noncompetitive, negative allosteric modulators	Phase 1: First Time in Human Study Evaluating the Safety, Tolerability, Pharmacokinetics, Pharmacodynamics and the Effect of Food of Single Assending Doses of GSK1482160. Clinical trial number: NCT00849134	Pain from inflammatory disease such as arthritis	(Di Virgilio et al., 2017; Han et al., 2017)
GW791343	Small molecule (positive modulator)	Negative allosteric modulator of human P2X7 but positive allosteric modulator of rat P2X7 at high concentrations.	Inter-subunit allosteric pocket involving F103 and V312 residues.	Preclinical	N/A	(Michel et al., 2008a; Karasawa and Kawate, 2016)
CE-224535 (Pfizer)	Small molecule	4 nM (Yo-Pro uptake) 1 nM (IL-1β secretion)	noncompetitive, negative allosteric modulators	Phase 2a: Efficacy and safety of CE-224,535, an antagonist of P2X7 receptor, in treatment of patients with rheumatoid arthritis inadequately controlled by methotrexate. Clinical trial number: NCT00628095;	Rheumatoid arthritis	(Duplantier et al., 2011; Di Virgilio et al., 2017)
EVT-401 (Evotec)	Small molecule	Not known	Not known	Phase 1 in healthy volunteers to investigate the safety, tolerability, pharmacokinetics and pharmacodynamics of EVT-401.	Inflammatory conditions such as Rheumatoid Arthritis	M.G. Kelly, J. Kincaid, Bicycloheteroaryl compounds as P2X7 modulators and uses thereof, US 7297700, 2007. Evotec Announces the Successful Completion of the First Phase 1 Study with EVT 401: https://www.evotec.com/en/invest/news–announcements/press-releases/p/evotec-announces-the-successful-completion-of-the-first-phase-i-study-with-evt-401-an-oral-p2x7-receptor-antagonist—very-good-safety-profile-and-confirmed-on-target-activity-4447
JNJ47965567	Small molecule	5 nM (Ca^2+^ influx)	Inter-subunit allosteric pocket involving F88, F103, M105, F108 and V312 residues.	Preclinical	N/A	(Bhattacharya et al., 2013; Karasawa and Kawate, 2016)
JNJ42253432	Small molecule	20nM (Ca^2+^ influx)	Not known	Preclinical	N/A	(Lord et al., 2014)
JNJ54232334	Small molecule	0.3 nM against human P2X7, 32 nM against rat P2X7 (Ca^2+^ influx)	Not known	Preclinical	N/A	(Lord et al., 2015)
JNJ54140515	Small molecule	79 nM against rat P2X7 (Ca^2+^ influx)	Not known	Preclinical	N/A	(Lord et al., 2015)
JNJ54166060	Small molecule	4 nM (Ca^2+^ influx)	Not known Inhibits CYP3A (IC50 = 2 μM)	Preclinical	N/A	(Swanson et al., 2016)
**Biologics**						
L4	mAb	5nM (change in current)	Not known	Preclinical	N/A	(Buell et al., 1998b)
Hano43	Rat mAb	N/A	Not known	N/A	N/A	(Adriouch et al., 2008)
Hano44	Rat mAb	N/A	Paratope includes R151 in finger-like structure cysteine-rich region	N/A	N/A	(Adriouch et al., 2008)
K1G	Rabbit polyclonal Ab.	N/A	Paratope includes R151 in finger-like structure cysteine-rich region	N/A	N/A	(Adriouch et al., 2008)
1F11	Rat mAb	N/A	Not known	N/A	N/A	(Kurashima et al., 2012)
13A7 nanobody	Bivalent nanobody-Fc	0.5 nM (inhibit CD62L shedding in mouse cell line)	Bind finger-like structure cysteine-rich region (same region as Hano44) mouse P2X7. No binding to human P2X7	N/A	N/A	(Danquah et al., 2016)
14D5 nanobody (enhancer)	Bivalent nanobody-Fc	0.1 nM (enhanced CD62L shedding in mouse cell line)	Bind finger-like structure cysteine-rich region (same region as Hano44) mouse P2X7. No binding to human P2X7	N/A	N/A	(Danquah et al., 2016)
Dano1-Fc	Bivalent nanobody-Fc	0.2 nM (Ca^2+^ influx and DAPI uptake)	Not known	Preclinical	N/A	(Danquah et al., 2016)
BIL010t	Polyclonal Anti-nfP2X7 ointment	N/A	E200 peptide in P2X7 extracellular domain	Investigation of the Safety and Tolerability of BSCT (Anti-nf-P2X7) 10% Ointment (Completed, has results) Clinical trial number: NCT02587819	Basal Cell carcinoma (BCC)	(Gilbert et al., 2017)
BIL06v	Vaccine	N/A	E200 peptide in P2X7 extracellular domain	A Phase 1 Study to Evaluate the Safety, Tolerability, Immunogenicity and Antitumor Activity of BIL06v/Alhydrogel in Patients with Advanced Solid Tumors. (Active, not recruiting) Clinical trial number: ACTRN12618000838213	Solid Tumors	

Several of these small molecules including AZD9056 (AstraZeneca), CE-224,535 (Pfizer), EVT-401 (Evotec), and GSK1482160 (GlaxoSmithKline plc) have entered clinical studies to treat rheumatoid arthritis as well as other inflammatory conditions (**Table 2**). Although all clinical data are not yet available, no clear benefits to patients have been observed so far (Jacobson and Muller, 2016; Di Virgilio et al., 2017). A recent report of an anti-P2X7 bivalent nanobody-Fc with IC_50 _in the sub-nanomolar range developed by Ablynx (now Sanofi) appear to show promising preclinical efficacy in chronic inflammatory disease models (Danquah et al., 2016). As reports increasingly highlight the critical role of P2X7 and ATP in tumor biology and its microenvironment, the rationale for targeting P2X7 in cancer in single or combination therapies is becoming increasingly relevant (Di Virgilio et al., 2017; Li et al., 2019). Indeed, the recent report of the well-tolerated BIL010t, topical anti-P2X7 treatment of BCC (Biosceptre) revealed promising patient outcomes with 65% of patients treated showing reduced lesion area and 20% with stable disease (Gilbert et al., 2017). Assessment of safety and immunogenicity of an anti P2X7 vaccine (BIL06v – Biosceptre) used in patients with advanced solid tumors is ongoing in Australia.

## Conclusion

Since the discovery of P2X7 as a cytotoxic receptor, studies have shown that the role of P2X7 in tumor biology and its microenvironment is much more complex than initially thought. Because of the significant involvement of P2X7 in the diverse mechanisms that drive tumor progression, this receptor is now believed to be of significant value as a target for the development of new cancer therapies. Preclinical and clinical evidence of P2X7-targeted therapeutics demonstrate the potential for these candidates as innovative cancer therapies. Furthermore, the interplay between the activities of P2X7 in tumor cell biology and antitumor immune responses indicates that P2X7 targeted therapies could also be of significant value when used in combination therapies. Therefore, additional studies, to better link the molecular mechanisms associated with P2X7 activation, signaling and cancer development, are now needed to drive highly innovative, safe and effective first in class therapeutics into the clinic.

## Author Contributions

Conception and design: RL, SM, EA, and FV. Writing, review, and/or revision of the manuscript: RL, EA, CH, MP, JB, FV, and SM.

## Funding

CH and MP, together with Biopsceptre (UK) Limited, have received a CASE Award from the UK Research Council [Biotechnology and Biological Sciences Research Council (BBSRC) (Reference No. BB/M009513/1)] to support a Doctoral Training Partnership. Funder role: Function of P2X7 in healthy and diseased human skin.

EA is supported by the Italian Association for Cancer Research, IG grant IG22837. Funder role: support for the investigation of the purinergic-adenosinergic axis in inflammation leading to colon cancer development.

FV is supported by the following grants:

- Italian Association for Cancer Research, IG grant 22883. Funder role: support for the investigation of repurposing purinergic signalling for cancer therapy.
- Italian Association for Cancer Research IG grant n.18581. Funder role: support for the investigation of the role of the P2X7 receptor in reprogramming cancer cell metabolism.
- European Commission, Grant EU COST BM1406. Funder role: support for exchange of young investigators between laboratories involved in the investigation of P2X7 role in immune response.
- Royal Society Exchange Fellowship IES\R3\170196. Funder role: support for the exchange of investigators involved in the study of P2X7-dependent microvesicle shedding from mononuclear cells.
- Projects of National Interest (PRIN) Ministry of Education of Italy, grant n. 20178YTNWC. Funder role: support for investigation of the mechanism responsible for P2X7 receptor-mediated release of pro-inflammatory cytokines from mononuclear phagocytes.

## Conflict of Interest

RL is an employee of Biosceptre (UK) Ltd.; JB is an employee of Biosceptre international Ltd.,; SM was an employee of Biosceptre (UK) Ltd; FV serves as a member of Biosceptre Scientific Advisory Board. Biosceptre is a company commercializing nfP2X7 targeted therapies. CH and MP have received a CASE Award together with Biosceptre (UK) Limited to support a Doctoral Training Partnership from the UK Research Council [Biotechnology and Biological Sciences Research Council (BBSRC) (Reference No. BB/M009513/1)].

The remaining authors declare that the research was conducted in the absence of any commercial or financial relationships that could be construed as a potential conflict of interest..

## References

[B1] Abbracchio M. P. Burnstock G. (1994). Purinoceptors: are there families of P2X and P2Y purinoceptors? Pharmacol. Ther. 64 (3), 445–475. 10.1016/0163-7258(94)00048-4 7724657

[B2] Adinolfi E. Melchiorri L. Falzoni S. Chiozzi P. Morelli A. Tieghi A. (2002). P2X7 receptor expression in evolutive and indolent forms of chronic B lymphocytic leukemia. Blood 99 (2), 706–708. 10.1182/blood.V99.2.706 11781259

[B3] Adinolfi E. Kim M. Young M. T. Di Virgilio F. Surprenant A. (2003). Tyrosine phosphorylation of HSP90 within the P2X7 receptor complex negatively regulates P2X7 receptors. J. Biol. Chem. 278 (39), 37344–37351. 10.1074/jbc.M301508200 12869560

[B4] Adinolfi E. Callegari M. G. Ferrari D. Bolognesi C. Minelli M. Wieckowski M. R. (2005). Basal activation of the P2X7 ATP receptor elevates mitochondrial calcium and potential, increases cellular ATP levels, and promotes serum-independent growth. Mol. Biol. Cell 16 (7), 3260–3272. 10.1091/mbc.E04-11-1025 15901833 PMC1165409

[B5] Adinolfi E. Callegari M. G. Cirillo M. Pinton P. Giorgi C. Cavagna D. (2009). Expression of the P2X7 receptor increases the Ca2+ content of the endoplasmic reticulum, activates NFATc1, and protects from apoptosis. J. Biol. Chem. 284 (15), 10120–10128. 10.1074/jbc.M805805200 19204004 PMC2665066

[B6] Adinolfi E. Cirillo M. Woltersdorf R. Falzoni S. Chiozzi P. Pellegatti P. (2010). Trophic activity of a naturally occurring truncated isoform of the P2X7 receptor. FASEB J. 24 (9), 3393–3404. 10.1096/fj.09-153601 20453110

[B7] Adinolfi E. Raffaghello L. Giuliani A. L. Cavazzini L. Capece M. Chiozzi P. (2012a). Expression of P2X7 receptor increases in vivo tumor growth. Cancer Res. 72 (12), 2957–2969. 10.1158/0008-5472.CAN-11-1947 22505653

[B8] Adinolfi E. Amoroso F. Giuliani A. L. (2012b). P2X7 Receptor Function in Bone-Related Cancer. J. Osteoporos 2012, 637863. 10.1155/2012/637863 22970409 PMC3431089

[B9] Adinolfi E. Capece M. Franceschini A. Falzoni S. Giuliani A. L. Rotondo A. (2015a). Accelerated tumor progression in mice lacking the ATP receptor P2X7. Cancer Res. 75 (4), 635–644. 10.1158/0008-5472.CAN-14-1259 25542861

[B10] Adinolfi E. Capece M. Amoroso F. De Marchi E. Franceschini A. (2015b). Emerging roles of P2X receptors in cancer. Curr. Med. Chem. 22 (7), 878–890. 10.2174/0929867321666141012172913 25312206

[B11] Adinolfi E. Giuliani A. L. De Marchi E. Pegoraro A. Orioli E. Di Virgilio F. (2018). The P2X7 receptor: A main player in inflammation. Biochem. Pharmacol. 151, 234–244. 10.1016/j.bcp.2017.12.021 29288626

[B12] Adinolfi E. De Marchi E. Orioli E. Pegoraro A. Di Virgilio F. (2019). Role of the P2X7 receptor in tumor-associated inflammation. Curr. Opin. Pharmacol. 47, 59–64. 10.1016/j.coph.2019.02.012 30921559

[B13] Adriouch S. Bannas P. Schwarz N. Fliegert R. Guse A. H. Seman M. (2008). ADP-ribosylation at R125 gates the P2X7 ion channel by presenting a covalent ligand to its nucleotide binding site. FASEB J. 22 (3), 861–869. 10.1096/fj.07-9294com 17928361

[B14] Agrawal A. Henriksen Z. Syberg S. Petersen S. Aslan D. Solgaard M. (2017). P2X7Rs are involved in cell death, growth and cellular signaling in primary human osteoblasts. Bone 95, 91–101. 10.1016/j.bone.2016.11.011 27856358

[B15] Allard B. Longhi M. S. Robson S. C. Stagg J. (2017). The ectonucleotidases CD39 and CD73: Novel checkpoint inhibitor targets. Immunol. Rev. 276 (1), 121–144. 10.1111/imr.12528 28258700 PMC5338647

[B16] Allsopp R. C. Dayl S. Schmid R. Evans R. J. (2017). Unique residues in the ATP gated human P2X7 receptor define a novel allosteric binding pocket for the selective antagonist AZ10606120. Sci. Rep. 7 (1), 725. 10.1038/s41598-017-00732-5 28389651 PMC5429621

[B17] Allsopp R. C. Dayl S. Bin Dayel A. Schmid R. Evans R. J. (2018). Mapping the Allosteric Action of Antagonists A740003 and A438079 Reveals a Role for the Left Flipper in Ligand Sensitivity at P2X7 Receptors. Mol. Pharmacol. 93 (5), 553–562. 10.1124/mol.117.111021 29535152 PMC5896373

[B18] Alqallaf S. M. Evans B. A. Kidd E. J. (2009). Atypical P2X receptor pharmacology in two human osteoblast-like cell lines. Br. J. Pharmacol. 156 (7), 1124–1135. 10.1111/j.1476-5381.2009.00119.x 19226284 PMC2697685

[B19] Amoroso F. Falzoni S. Adinolfi E. Ferrari D. Di Virgilio F. (2012). The P2X7 receptor is a key modulator of aerobic glycolysis. Cell Death Dis. 3, e370. 10.1038/cddis.2012.105 22898868 PMC3434661

[B20] Amoroso F. Capece M. Rotondo A. Cangelosi D. Ferracin M. Franceschini A. (2015). The P2X7 receptor is a key modulator of the PI3K/GSK3beta/VEGF signaling network: evidence in experimental neuroblastoma. Oncogene 34 (41), 5240–5251. 10.1038/onc.2014.444 25619831

[B21] Amoroso F. Salaro E. Falzoni S. Chiozzi P. Giuliani A. L. Cavallesco G. (2016). P2X7 targeting inhibits growth of human mesothelioma. Oncotarget 7 (31), 49664–49676. 10.18632/oncotarget.10430 27391069 PMC5226537

[B22] Amstrup J. Novak I. (2003). P2X7 receptor activates extracellular signal-regulated kinases ERK1 and ERK2 independently of Ca2+ influx. Biochem. J. 374 (Pt 1), 51–61. 10.1042/BJ20030585 12747800 PMC1223572

[B23] Andrei C. Margiocco P. Poggi A. Lotti L. V. Torrisi M. R. Rubartelli A. (2004). Phospholipases C and A2 control lysosome-mediated IL-1 beta secretion: Implications for inflammatory processes. Proc. Natl. Acad. Sci. U. S. A. 101 (26), 9745–9750. 10.1073/pnas.0308558101 15192144 PMC470745

[B24] Antonioli L. Blandizzi C. Pacher P. Hasko G. (2013). Immunity, inflammation and cancer: a leading role for adenosine. Nat. Rev. Cancer 13 (12), 842–857. 10.1038/nrc3613 24226193

[B25] Arulkumaran N. Unwin R. J. Tam F. W. (2011). A potential therapeutic role for P2X7 receptor (P2X7R) antagonists in the treatment of inflammatory diseases. Expert Opin. Invest. Drugs 20 (7), 897–915. 10.1517/13543784.2011.578068 PMC3114873 21510825

[B26] Aswad F. Kawamura H. Dennert G. (2005). High sensitivity of CD4+CD25+ regulatory T cells to extracellular metabolites nicotinamide adenine dinucleotide and ATP: a role for P2X7 receptors. J. Immunol. 175 (5), 3075–383. 10.4049/jimmunol.175.5.3075 16116196

[B27] Atarashi K. Nishimura J. Shima T. Umesaki Y. Yamamoto M. Onoue M. (2008). ATP drives lamina propria T(H)17 cell differentiation. Nature 455 (7214), 808–812. 10.1038/nature07240 18716618

[B28] Babelova A. Moreth K. Tsalastra-Greul W. Zeng-Brouwers J. Eickelberg O. Young M. F. (2009). Biglycan, a danger signal that activates the NLRP3 inflammasome via toll-like and P2X receptors. J. Biol. Chem. 284 (36), 24035–24048. 10.1074/jbc.M109.014266 19605353 PMC2781998

[B29] Bae J. Y. Lee S. W. Shin Y. H. Lee J. H. Jahng J. W. Park K. (2017). P2X7 receptor and NLRP3 inflammasome activation in head and neck cancer. Oncotarget. 8 (30), 48972–48982. 10.18632/oncotarget.16903 28430665 PMC5564741

[B30] Bailis W. Shyer J. A. Zhao J. Canaveras J. C. G. Al Khazal F. J. Qu R. (2019). Distinct modes of mitochondrial metabolism uncouple T cell differentiation and function. Nature 571 (7765), 403–407. 10.1038/s41586-019-1311-3 31217581 PMC6939459

[B31] Bannas P. Adriouch S. Kahl S. Braasch F. Haag F. Koch-Nolte F. (2005). Activity and specificity of toxin-related mouse T cell ecto-ADP-ribosyltransferase ART2.2 depends on its association with lipid rafts. Blood 105 (9), 3663–3670. 10.1182/blood-2004-08-3325 15657180

[B32] Barbera-Cremades M. Baroja-Mazo A. Gomez A. I. Machado F. Di Virgilio F. Pelegrin P. (2012). P2X7 receptor-stimulation causes fever via PGE2 and IL-1beta release. FASEB J. 26 (7), 2951–2962. 10.1096/fj.12-205765 22490780

[B33] Barden J. A. Sluyter R. Gu B. J. Wiley J. S. (2003). Specific detection of non-functional human P2X(7) receptors in HEK293 cells and B-lymphocytes. FEBS Lett. 538 (1-3), 159–162. 10.1016/S0014-5793(03)00172-8 12633871

[B34] Barden J. Gidley-Baird A. Armstrong A. Bean P. Pilkington G. Sempf F. (2009). Abstract #LB-153: Evaluation of non-functional P2X7 receptor as a potential pan cancer therapeutic and diagnostic target. Cancer Res. 69 (9).

[B35] Barden J. A. Yuksel A. Pedersten J. Danieletto S. Delprado W. (2014). Non-Functional P2X7: A Novel and Ubiquitous Target in Human Cancer. J. Clin. Cell Immunol. 5 (4), 237. 10.4172/2155-9899.1000237

[B36] Barden J. A. Gidley-Baird A. Teh L. C. Rajasekariah G. H. Pedersen J. Christensen N. I. (2016). Therapeutic Targeting of the Cancer-Specific Cell Surface Biomarker nfP2X7. J. Clin. Cell. Immunol. 7 (3), 432. 10.4172/2155-9899.1000432

[B37] Baricordi O. R. Ferrari D. Melchiorri L. Chiozzi P. Hanau S. Chiari E. (1996). An ATP-activated channel is involved in mitogenic stimulation of human T lymphocytes. Blood 87 (2), 682–690. 10.1182/blood.V87.2.682.bloodjournal872682 8555491

[B38] Baricordi O. R. Melchiorri L. Adinolfi E. Falzoni S. Chiozzi P. Buell G. (1999). Increased proliferation rate of lymphoid cells transfected with the P2X(7) ATP receptor. J. Biol. Chem. 274 (47), 33206–33208. 10.1074/jbc.274.47.33206 10559192

[B39] Baroni M. Pizzirani C. Pinotti M. Ferrari D. Adinolfi E. Calzavarini S. (2007). Stimulation of P2 (P2X7) receptors in human dendritic cells induces the release of tissue factor-bearing microparticles. FASEB J. 21 (8), 1926–1933. 10.1096/fj.06-7238com 17314141

[B40] Barth K. Weinhold K. Guenther A. Young M. T. Schnittler H. Kasper M. (2007). Caveolin-1 influences P2X7 receptor expression and localization in mouse lung alveolar epithelial cells. FEBS J. 274 (12), 3021–3033. 10.1111/j.1742-4658.2007.05830.x 17498208

[B41] Baudelet D. Lipka E. Millet R. Ghinet A. (2015). Involvement of the P2X7 purinergic receptor in inflammation: an update of antagonists series since 2009 and their promising therapeutic potential. Curr. Med. Chem. 22 (6), 713–729. 10.2174/0929867322666141212120926 25515510

[B42] Beldi G. Wu Y. Banz Y. Nowak M. Miller L. Enjyoji K. (2008). Natural killer T cell dysfunction in CD39-null mice protects against concanavalin A-induced hepatitis. Hepatology 48 (3), 841–852. 10.1002/hep.22401 18752325 PMC2929828

[B43] Benzaquen J. Heeke S. Janho Dit Hreich S. Douguet L. Marquette C. H. Hofman P. (2019). Alternative splicing of P2RX7 pre-messenger RNA in health and diseases: Myth or reality? BioMed. J. 42 (3), 141–154. 10.1016/j.bj.2019.05.007 31466708 PMC6717933

[B44] Bergamin L. S. Capece M. Salaro E. Sarti A. C. Falzoni S. Pereira M. S. L. (2019). Role of the P2X7 receptor in in vitro and in vivo glioma tumor growth. Oncotarget 10 (47), 4840–4856. 10.18632/oncotarget.27106 31448051 PMC6690673

[B45] Bhattacharya A. Wang Q. Ao H. Shoblock J. R. Lord B. Aluisio L. (2013). Pharmacological characterization of a novel centrally permeable P2X7 receptor antagonist: JNJ-47965567. Br. J. Pharmacol. 170 (3), 624–640. 10.1111/bph.12314 23889535 PMC3792000

[B46] Bian S. Sun X. Bai A. Zhang C. Li L. Enjyoji K. (2013). P2X7 integrates PI3K/AKT and AMPK-PRAS40-mTOR signaling pathways to mediate tumor cell death. PloS One 8 (4), e60184. 10.1371/journal.pone.0060184 23565201 PMC3615040

[B47] Bianchi G. Vuerich M. Pellegatti P. Marimpietri D. Emionite L. Marigo I. (2014). ATP/P2X7 axis modulates myeloid-derived suppressor cell functions in neuroblastoma microenvironment. Cell Death Dis. 5, e1135. 10.1038/cddis.2014.109 24651438 PMC3973218

[B48] Bianco F. Pravettoni E. Colombo A. Schenk U. Moller T. Matteoli M. (2005). Astrocyte-derived ATP induces vesicle shedding and IL-1 beta release from microglia. J. Immunol. 174 (11), 7268–7277. 10.4049/jimmunol.174.11.7268 15905573

[B49] Bianco F. Ceruti S. Colombo A. Fumagalli M. Ferrari D. Pizzirani C. (2006). A role for P2X7 in microglial proliferation. J. Neurochem. 99 (3), 745–758. 10.1111/j.1471-4159.2006.04101.x 16836656

[B50] Bianco F. Perrotta C. Novellino L. Francolini M. Riganti L. Menna E. (2009). Acid sphingomyelinase activity triggers microparticle release from glial cells. EMBO J. 28 (8), 1043–1054. 10.1038/emboj.2009.45 19300439 PMC2664656

[B51] Bin Dayel A. Evans R. J. Schmid R. (2019). Mapping the Site of Action of Human P2X7 Receptor Antagonists AZ11645373, Brilliant Blue G, KN-62, Calmidazolium, and ZINC58368839 to the Intersubunit Allosteric Pocket. Mol. Pharmacol. 96 (3), 355–363. 10.1124/mol.119.116715 31263019 PMC6701605

[B52] Boldt W. Klapperstuck M. Buttner C. Sadtler S. Schmalzing G. Markwardt F. (2003). Glu496Ala polymorphism of human P2X7 receptor does not affect its electrophysiological phenotype. Am. J. Physiol. Cell Physiol. 284 (3), C749–C756. 10.1152/ajpcell.00042.2002 12431909

[B53] Borges da Silva H. Beura L. K. Wang H. Hanse E. A. Gore R. Scott M. C. (2018). The purinergic receptor P2RX7 directs metabolic fitness of long-lived memory CD8(+) T cells. Nature 559 (7713), 264–268. 10.1038/s41586-018-0282-0 29973721 PMC6054485

[B54] Bos J. L. Rehmann H. Wittinghofer A. (2007). GEFs and GAPs: critical elements in the control of small G proteins. Cell 129 (5), 865–877. 10.1016/j.cell.2007.05.018 17540168

[B55] Boumechache M. Masin M. Edwardson J. M. Gorecki D. C. Murrell-Lagnado R. (2009). Analysis of assembly and trafficking of native P2X4 and P2X7 receptor complexes in rodent immune cells. J. Biol. Chem. 284 (20), 13446–13454. 10.1074/jbc.M901255200 19304656 PMC2679444

[B56] Bradford M. D. Soltoff S. P. (2002). P2X7 receptors activate protein kinase D and p42/p44 mitogen-activated protein kinase (MAPK) downstream of protein kinase C. Biochem. J. 366 (Pt 3), 745–755. 10.1042/BJ20020358 12057008 PMC1222820

[B57] Bradley H. J. Baldwin J. M. Goli G. R. Johnson B. Zou J. Sivaprasadarao A. (2011). Residues 155 and 348 contribute to the determination of P2X7 receptor function via distinct mechanisms revealed by single-nucleotide polymorphisms. J. Biol. Chem. 286 (10), 8176–8187. 10.1074/jbc.M110.211284 21205829 PMC3048704

[B58] Brake A. J. Wagenbach M. J. Julius D. (1994). New structural motif for ligand-gated ion channels defined by an ionotropic ATP receptor. Nature 371 (6497), 519–523. 10.1038/371519a0 7523952

[B59] Buell G. N. Talabot F. Gos A. Lorenz J. Lai E. Morris M. A. (1998a). Gene structure and chromosomal localization of the human P2X7 receptor. Recept. Channels 5 (6), 347–354. 9826911

[B60] Buell G. Chessell I. P. Michel A. D. Collo G. Salazzo M. Herren S. (1998b). Blockade of human P2X7 receptor function with a monoclonal antibody. Blood 92 (10), 3521–3528. 10.1182/blood.V92.10.3521 9808543

[B61] Burnstock G. (2006). Purinergic signalling. Br. J. Pharmacol. 147 Suppl 1, S172–S181. 10.1038/sj.bjp.0706429 16402102 PMC1760723

[B62] Burnstock G. (2014). Purinergic signalling: from discovery to current developments. Exp. Physiol. 99 (1), 16–34. 10.1113/expphysiol.2013.071951 24078669 PMC4208685

[B63] Cabrini G. Falzoni S. Forchap S. L. Pellegatti P. Balboni A. Agostini P. (2005). A His-155 to Tyr polymorphism confers gain-of-function to the human P2X7 receptor of human leukemic lymphocytes. J. Immunol. 175 (1), 82–89. 10.4049/jimmunol.175.1.82 15972634

[B64] Calik I. Calik M. Sarikaya B. Ozercan I. H. Arslan R. Artas G. (2020). P2X7R as an independent prognostic indicator in gastric cancer. Bosn. J. Basic Med. Sci. 20 (2), 188–196. 10.17305/bjbms.2020.4620 32070268 PMC7202194

[B65] Caseley E. A. Muench S. P. Baldwin S. A. Simmons K. Fishwick C. W. Jiang L. H. (2015). Docking of competitive inhibitors to the P2X7 receptor family reveals key differences responsible for changes in response between rat and human. Bioorg. Med. Chem. Lett. 25 (16), 3164–3167. 10.1016/j.bmcl.2015.06.001 26099538 PMC4508345

[B66] Chadet S. Jelassi B. Wannous R. Angoulvant D. Chevalier S. Besson P. (2014). The activation of P2Y2 receptors increases MCF-7 breast cancer cells migration through the MEK-ERK1/2 signalling pathway. Carcinogenesis 35 (6), 1238–1247. 10.1093/carcin/bgt493 24390819

[B67] Chaumont S. Jiang L. H. Penna A. North R. A. Rassendren F. (2004). Identification of a trafficking motif involved in the stabilization and polarization of P2X receptors. J. Biol. Chem. 279 (28), 29628–29638. 10.1074/jbc.M403940200 15126501

[B68] Cheewatrakoolpong B. Gilchrest H. Anthes J. C. Greenfeder S. (2005). Identification and characterization of splice variants of the human P2X7 ATP channel. Biochem. Biophys. Res. Commun. 332 (1), 17–27. 10.1016/j.bbrc.2005.04.087 15896293

[B69] Chen S. Feng W. Yang X. Yang W. Ru Y. Liao J. (2014). Functional expression of P2X family receptors in macrophages is affected by microenvironment in mouse T cell acute lymphoblastic leukemia. Biochem. Biophys. Res. Commun. 446 (4), 1002–1009. 10.1016/j.bbrc.2014.03.048 24661878

[B70] Chessell I. P. Hatcher J. P. Bountra C. Michel A. D. Hughes J. P. Green P. (2005). Disruption of the P2X7 purinoceptor gene abolishes chronic inflammatory and neuropathic pain. Pain 114 (3), 386–396. 10.1016/j.pain.2005.01.002 15777864

[B71] Choi J. H. Ji Y. G. Ko J. J. Cho H. J. Lee D. H. (2018). Activating P2X7 Receptors Increases Proliferation of Human Pancreatic Cancer Cells via ERK1/2 and JNK. Pancreas 47 (5), 643–651. 10.1097/MPA.0000000000001055 29683976

[B72] Chong J. H. Zheng G. G. Zhu X. F. Guo Y. Wang L. Ma C. H. (2010a). Abnormal expression of P2X family receptors in Chinese pediatric acute leukemias. Biochem. Biophys. Res. Commun. 391 (1), 498–504. 10.1016/j.bbrc.2009.11.087 19919827

[B73] Chong J. H. Zheng G. G. Ma Y. Y. Zhang H. Y. Nie K. Lin Y. M. (2010b). The hyposensitive N187D P2X7 mutant promotes malignant progression in nude mice. J. Biol. Chem. 285 (46), 36179–36187. 10.1074/jbc.M110.128488 20837475 PMC2975240

[B74] Cockcroft S. Gomperts B. D. (1979). ATP induces nucleotide permeability in rat mast cells. Nature 279 (5713), 541–542. 10.1038/279541a0 450099

[B75] Coddou C. Yan Z. Obsil T. Huidobro-Toro J. P. Stojilkovic S. S. (2011). Activation and regulation of purinergic P2X receptor channels. Pharmacol. Rev. 63 (3), 641–683. 10.1124/pr.110.003129 21737531 PMC3141880

[B76] Constantinescu P. Wang B. Kovacevic K. Jalilian I. Bosman G. J. Wiley J. S. (2010). P2X7 receptor activation induces cell death and microparticle release in murine erythroleukemia cells. Biochim. Biophys. Acta 1798 (9), 1797–1804. 10.1016/j.bbamem.2010.06.002 20529664

[B77] Costa-Junior H. M. Sarmento Vieira F. Coutinho-Silva R. (2011). C terminus of the P2X7 receptor: treasure hunting. Purinerg. Signal 7 (1), 7–19. 10.1007/s11302-011-9215-1 PMC3083127 21484094

[B78] Coutinho-Silva R. Stahl L. Raymond M. N. Jungas T. Verbeke P. Burnstock G. (2003). Inhibition of chlamydial infectious activity due to P2X7R-dependent phospholipase D activation. Immunity 19 (3), 403–412. 10.1016/s1074-7613(03)00235-8 14499115

[B79] Coutinho-Silva R. Stahl L. Cheung K. K. de Campos N. E. de Oliveira Souza C. Ojcius D. M. (2005). P2X and P2Y purinergic receptors on human intestinal epithelial carcinoma cells: effects of extracellular nucleotides on apoptosis and cell proliferation. Am. J. Physiol. Gastrointest. Liver Physiol. 288 (5), G1024–G1035. 10.1152/ajpgi.00211.2004 15662049

[B80] D’Autreaux B. Toledano M. B. (2007). ROS as signalling molecules: mechanisms that generate specificity in ROS homeostasis. Nat. Rev. Mol. Cell Biol. 8 (10), 813–824. 10.1038/nrm2256 17848967

[B81] Dagvadorj J. Shimada K. Chen S. Jones H. D. Tumurkhuu G. Zhang W. (2015). Lipopolysaccharide Induces Alveolar Macrophage Necrosis via CD14 and the P2X7 Receptor Leading to Interleukin-1alpha Release. Immunity 42 (4), 640–653. 10.1016/j.immuni.2015.03.007 25862090 PMC4423803

[B82] Danquah W. Meyer-Schwesinger C. Rissiek B. Pinto C. Serracant-Prat A. Amadi M. (2016). Nanobodies that block gating of the P2X7 ion channel ameliorate inflammation. Sci. Transl. Med. 8 (366), 366ra162. 10.1126/scitranslmed.aaf8463 27881823

[B83] Dardano A. Falzoni S. Caraccio N. Polini A. Tognini S. Solini A. (2009). 1513A>C polymorphism in the P2X7 receptor gene in patients with papillary thyroid cancer: correlation with histological variants and clinical parameters. J. Clin. Endocrinol. Metab. 94 (2), 695–698. 10.1210/jc.2008-1322 19017759

[B84] de Andrade Mello P. Coutinho-Silva R. Savio L. E. B. (2017). Multifaceted Effects of Extracellular Adenosine Triphosphate and Adenosine in the Tumor-Host Interaction and Therapeutic Perspectives. Front. Immunol. 8, 1526. 10.3389/fimmu.2017.01526 29184552 PMC5694450

[B85] De Marchi E. Orioli E. Dal Ben D. Adinolfi E. (2016). P2X7 Receptor as a Therapeutic Target. Adv. Protein Chem. Struct. Biol. 104, 39–79. 10.1016/bs.apcsb.2015.11.004 27038372

[B86] De Marchi E. Orioli E. Pegoraro A. Sangaletti S. Portararo P. Curti A. (2019). The P2X7 receptor modulates immune cells infiltration, ectonucleotidases expression and extracellular ATP levels in the tumor microenvironment. Oncogene 38 (19), 3636–3650. 10.1038/s41388-019-0684-y 30655604 PMC6756114

[B87] de Torre-Minguela C. Barbera-Cremades M. Gomez A. I. Martin-Sanchez F. Pelegrin P. (2016). Macrophage activation and polarization modify P2X7 receptor secretome influencing the inflammatory process. Sci. Rep. 6, 22586. 10.1038/srep22586 26935289 PMC4776275

[B88] Denlinger L. C. Fisette P. L. Sommer J. A. Watters J. J. Prabhu U. Dubyak G. R. (2001). Cutting edge: the nucleotide receptor P2X7 contains multiple protein- and lipid-interaction motifs including a potential binding site for bacterial lipopolysaccharide. J. Immunol. 167 (4), 1871–1876. 10.4049/jimmunol.167.4.1871 11489964

[B89] Denlinger L. C. Sommer J. A. Parker K. Gudipaty L. Fisette P. L. Watters J. W. (2003). Mutation of a dibasic amino acid motif within the C terminus of the P2X7 nucleotide receptor results in trafficking defects and impaired function. J. Immunol. 171 (3), 1304–1311. 10.4049/jimmunol.171.3.1304 12874219

[B90] Di A. Xiong S. Ye Z. Malireddi R. K. S. Kometani S. Zhong M. (2018). The TWIK2 Potassium Efflux Channel in Macrophages Mediates NLRP3 Inflammasome-Induced Inflammation. Immunity 49 (1), 56–65 e4. 10.1016/j.immuni.2018.04.032 29958799 PMC6051907

[B91] Di Virgilio F. Adinolfi E. (2017). Extracellular purines, purinergic receptors and tumor growth. Oncogene 36 (3), 293–303. 10.1038/onc.2016.206 27321181 PMC5269532

[B92] Di Virgilio F. Meyer B. C. Greenberg S. Silverstein S. C. (1988). Fc receptor-mediated phagocytosis occurs in macrophages at exceedingly low cytosolic Ca2+ levels. J. Cell Biol. 106 (3), 657–666. 10.1083/jcb.106.3.657 3346321 PMC2115077

[B93] Di Virgilio F. Chiozzi P. Falzoni S. Ferrari D. Sanz J. M. Venketaraman V. (1998). Cytolytic P2X purinoceptors. Cell Death Differ. 5 (3), 191–199. 10.1038/sj.cdd.4400341 10200464

[B94] Di Virgilio F. Ferrari D. Adinolfi E. (2009). P2X(7): a growth-promoting receptor-implications for cancer. Purinerg. Signal 5 (2), 251–256. 10.1007/s11302-009-9145-3 PMC2686832 19263244

[B95] Di Virgilio F. Falzoni S. Giuliani A. L. Adinolfi E. (2016). P2 receptors in cancer progression and metastatic spreading. Curr. Opin. Pharmacol. 29, 17–25. 10.1016/j.coph.2016.05.001 27262778

[B96] Di Virgilio F. Dal Ben D. Sarti A. C. Giuliani A. L. Falzoni S. (2017). The P2X7 Receptor in Infection and Inflammation. Immunity 47 (1), 15–31. 10.1016/j.immuni.2017.06.020 28723547

[B97] Di Virgilio F. Sarti A. C. Falzoni S. De Marchi E. Adinolfi E. (2018a). Extracellular ATP and P2 purinergic signalling in the tumour microenvironment. Nat. Rev. Cancer 18 (10), 601–618. 10.1038/s41568-018-0037-0 30006588

[B98] Di Virgilio F. Schmalzing G. Markwardt F. (2018b). The Elusive P2X7 Macropore. Trends Cell Biol. 28 (5), 392–404. 10.1016/j.tcb.2018.01.005 29439897

[B99] Di Virgilio F. Sarti A. C. Grassi F. (2018c). Modulation of innate and adaptive immunity by P2X ion channels. Curr. Opin. Immunol. 52, 51–59. 10.1016/j.coi.2018.03.026 29631184

[B100] Di Virgilio F. Jiang L. H. Roger S. Falzoni S. Sarti A. C. Vultaggio-Poma V. (2019). Structure, function and techniques of investigation of the P2X7 receptor (P2X7R) in mammalian cells. Methods Enzymol. 629, 115–150. 10.1016/bs.mie.2019.07.043 31727237

[B101] Di Virgilio F. (2015). P2X receptors and inflammation. Curr. Med. Chem. 22 (7), 866–877. 10.2174/0929867322666141210155311 25524252

[B102] Dinarello C. A. (2002). The IL-1 family and inflammatory diseases. Clin. Exp. Rheumatol. 20 (5 Suppl 27), S1–13. 14989423

[B103] Donnelly-Roberts D. L. Namovic M. T. Han P. Jarvis M. F. (2009a). Mammalian P2X7 receptor pharmacology: comparison of recombinant mouse, rat and human P2X7 receptors. Br. J. Pharmacol. 157 (7), 1203–1214. 10.1111/j.1476-5381.2009.00233.x 19558545 PMC2743839

[B104] Donnelly-Roberts D. L. Namovic M. T. Surber B. Vaidyanathan S. X. Perez-Medrano A. Wang Y. (2009b). [3H]A-804598 ([3H]2-cyano-1-[(1S)-1-phenylethyl]-3-quinolin-5-ylguanidine) is a novel, potent, and selective antagonist radioligand for P2X7 receptors. Neuropharmacology 56 (1), 223–229. 10.1016/j.neuropharm.2008.06.012 18602931

[B105] Draganov D. Gopalakrishna-Pillai S. Chen Y. R. Zuckerman N. Moeller S. Wang C. (2015). Modulation of P2X4/P2X7/Pannexin-1 sensitivity to extracellular ATP via Ivermectin induces a non-apoptotic and inflammatory form of cancer cell death. Sci. Rep. 5, 16222. 10.1038/srep16222 26552848 PMC4639773

[B106] Dubyak G. R. (2007). Go it alone no more–P2X7 joins the society of heteromeric ATP-gated receptor channels. Mol. Pharmacol. 72 (6), 1402–1405. 10.1124/mol.107.042077 17895406

[B107] Duplantier A. J. Dombroski M. A. Subramanyam C. Beaulieu A. M. Chang S. P. Gabel C. A. (2011). Optimization of the physicochemical and pharmacokinetic attributes in a 6-azauracil series of P2X7 receptor antagonists leading to the discovery of the clinical candidate CE-224,535. Bioorg. Med. Chem. Lett. 21 (12), 3708–3711. 10.1016/j.bmcl.2011.04.077 21565499

[B108] Dupre-Crochet S. Erard M. Nubetae O. (2013). ROS production in phagocytes: why, when, and where? J. Leukoc. Biol. 94 (4), 657–670. 10.1189/jlb.1012544 23610146

[B109] Eltzschig H. K. Sitkovsky M. V. Robson S. C. (2012). Purinergic signaling during inflammation. N Engl. J. Med. 367 (24), 2322–2333. 10.1056/NEJMra1205750 23234515 PMC3675791

[B110] Eser A. Colombel J. F. Rutgeerts P. Vermeire S. Vogelsang H. Braddock M. (2015). Safety and Efficacy of an Oral Inhibitor of the Purinergic Receptor P2X7 in Adult Patients with Moderately to Severely Active Crohn’s Disease: A Randomized Placebo-controlled, Double-blind, Phase IIa Study. Inflammation Bowel Dis. 21 (10), 2247–2253. 10.1097/MIB.0000000000000514 26197451

[B111] Esseltine J. L. Laird D. W. (2016). Next-Generation Connexin and Pannexin Cell Biology. Trends Cell Biol. 26 (12), 944–955. 10.1016/j.tcb.2016.06.003 27339936

[B112] Fabbrizio P. Amadio S. Apolloni S. Volonte C. (2017). P2X7 Receptor Activation Modulates Autophagy in SOD1-G93A Mouse Microglia. Front. Cell Neurosci. 11, 249. 10.3389/fncel.2017.00249 28871219 PMC5566572

[B113] Fang J. Chen X. Zhang L. Chen J. Liang Y. Li X. (2013). P2X7R suppression promotes glioma growth through epidermal growth factor receptor signal pathway. Int. J. Biochem. Cell Biol. 45 (6), 1109–1120. 10.1016/j.biocel.2013.03.005 23523696

[B114] Feng Y. H. Li X. Wang L. Zhou L. Gorodeski G. I. (2006). A truncated P2X7 receptor variant (P2X7-j) endogenously expressed in cervical cancer cells antagonizes the full-length P2X7 receptor through hetero-oligomerization. J. Biol. Chem. 281 (25), 17228–17237. 10.1074/jbc.M602999200 16624800 PMC2409001

[B115] Feng Y. H. Li X. Zeng R. Gorodeski G. I. (2006). Endogenously expressed truncated P2X7 receptor lacking the C-terminus is preferentially upregulated in epithelial cancer cells and fails to mediate ligand-induced pore formation and apoptosis. Nucleos. Nucleot. Nucleic Acids 25 (9-11), 1271–1276. 10.1080/15257770600890921 17065105

[B116] Ferrari D. Munerati M. Melchiorri L. Hanau S. di Virgilio F. Baricordi O. R. (1994). Responses to extracellular ATP of lymphoblastoid cell lines from Duchenne muscular dystrophy patients. Am. J. Physiol. 267 (4 Pt 1), C886–C892. 10.1152/ajpcell.1994.267.4.C886 7524344

[B117] Ferrari D. Chiozzi P. Falzoni S. Dal Susino M. Collo G. Buell G. (1997a). ATP-mediated cytotoxicity in microglial cells. Neuropharmacology 36 (9), 1295–1301. 10.1016/s0028-3908(97)00137-8 9364484

[B118] Ferrari D. Chiozzi P. Falzoni S. Dal Susino M. Melchiorri L. Baricordi O. R. (1997b). Extracellular ATP triggers IL-1 beta release by activating the purinergic P2Z receptor of human macrophages. J. Immunol. 159 (3), 1451–1458. 9233643

[B119] Ferrari D. La Sala A. Chiozzi P. Morelli A. Falzoni S. Girolomoni G. (2000). The P2 purinergic receptors of human dendritic cells: identification and coupling to cytokine release. FASEB J. 14 (15), 2466–2476. 10.1096/fj.00-0031com 11099464

[B120] Ferrari D. Pizzirani C. Adinolfi E. Lemoli R. M. Curti A. Idzko M. (2006). The P2X7 receptor: a key player in IL-1 processing and release. J. Immunol. 176 (7), 3877–3883. 10.4049/jimmunol.176.7.3877 16547218

[B121] Figliuolo V. R. Savio L. E. B. Safya H. Nanini H. Bernardazzi C. Abalo A. (2017). P2X7 receptor promotes intestinal inflammation in chemically induced colitis and triggers death of mucosal regulatory T cells. Biochim. Biophys. Acta 1863 (6), 1183–1194. 10.1016/j.bbadis.2017.03.004 28286160

[B122] Franceschini A. Capece M. Chiozzi P. Falzoni S. Sanz J. M. Sarti A. C. (2015). The P2X7 receptor directly interacts with the NLRP3 inflammasome scaffold protein. FASEB J. 29 (6), 2450–2461. 10.1096/fj.14-268714 25690658

[B123] Galluzzi L. Buque A. Kepp O. Zitvogel L. Kroemer G. (2017). Immunogenic cell death in cancer and infectious disease. Nat. Rev. Immunol. 17 (2), 97–111. 10.1038/nri.2016.107 27748397

[B124] Garcia-Marcos M. Perez-Andres E. Tandel S. Fontanils U. Kumps A. Kabre E. (2006). Coupling of two pools of P2X7 receptors to distinct intracellular signaling pathways in rat submandibular gland. J. Lipid Res. 47 (4), 705–714. 10.1194/jlr.M500408-JLR200 16415476

[B125] Gartland A. Hipskind R. A. Gallagher J. A. Bowler W. B. (2001). Expression of a P2X7 receptor by a subpopulation of human osteoblasts. J. Bone Miner. Res. 16 (5), 846–856. 10.1359/jbmr.2001.16.5.846 11341329

[B126] Gartland A. Skarratt K. K. Hocking L. J. Parsons C. Stokes L. Jorgensen N. R. (2012). Polymorphisms in the P2X7 receptor gene are associated with low lumbar spine bone mineral density and accelerated bone loss in post-menopausal women. Eur. J. Hum. Genet. 20 (5), 559–564. 10.1038/ejhg.2011.245 22234152 PMC3330223

[B127] Gavin M. A. Rasmussen J. P. Fontenot J. D. Vasta V. Manganiello V. C. Beavo J. A. (2007). Foxp3-dependent programme of regulatory T-cell differentiation. Nature 445 (7129), 771–775. 10.1038/nature05543 17220874

[B128] Gehring M. P. Pereira T. C. Zanin R. F. Borges M. C. Braga Filho A. Battastini A. M. (2012). P2X7 receptor activation leads to increased cell death in a radiosensitive human glioma cell line. Purinerg. Signal 8 (4), 729–739. 10.1007/s11302-012-9319-2 PMC3486168 22644907

[B129] Gehring M. P. Kipper F. Nicoletti N. F. Sperotto N. D. Zanin R. Tamajusuku A. S. (2015). P2X7 receptor as predictor gene for glioma radiosensitivity and median survival. Int. J. Biochem. Cell Biol. 68, 92–100. 10.1016/j.biocel.2015.09.001 26358881

[B130] Ghalali A. Wiklund F. Zheng H. Stenius U. Hogberg J. (2014). Atorvastatin prevents ATP-driven invasiveness via P2X7 and EHBP1 signaling in PTEN-expressing prostate cancer cells. Carcinogenesis 35 (7), 1547–1555. 10.1093/carcin/bgu019 24451147

[B131] Ghiringhelli F. Apetoh L. Tesniere A. Aymeric L. Ma Y. Ortiz C. (2009). Activation of the NLRP3 inflammasome in dendritic cells induces IL-1beta-dependent adaptive immunity against tumors. Nat. Med. 15 (10), 1170–1178. 10.1038/nm.2028 19767732

[B132] Giannuzzo A. Pedersen S. F. Novak I. (2015). The P2X7 receptor regulates cell survival, migration and invasion of pancreatic ductal adenocarcinoma cells. Mol. Cancer 14, 203. 10.1186/s12943-015-0472-4 26607222 PMC4660609

[B133] Giannuzzo A. Saccomano M. Napp J. Ellegaard M. Alves F. Novak I. (2016). Targeting of the P2X7 receptor in pancreatic cancer and stellate cells. Int. J. Cancer 139 (11), 2540–2552. 10.1002/ijc.30380 27513892 PMC5095874

[B134] Gidley-Baird A. Barden J. A. (2002). Antibodies to non-functional p2x7 receptor diagnosis and treatment of cancers and other conditions. Patent No: WO2003020762A1. Available at: https://patents.google.com/patent/WO2003020762A1/3Den

[B135] Gidlof O. Smith J. G. Melander O. Lovkvist H. Hedblad B. Engstrom G. (2012). A common missense variant in the ATP receptor P2X7 is associated with reduced risk of cardiovascular events. PloS One 7 (5), e37491. 10.1371/journal.pone.0037491 22662160 PMC3360776

[B136] Gilbert S. M. Gidley Baird A. Glazer S. Barden J. A. Glazer A. Teh L. C. (2017). A phase I clinical trial demonstrates that nfP2X7 -targeted antibodies provide a novel, safe and tolerable topical therapy for basal cell carcinoma. Br. J. Dermatol. 177 (1), 117–124. 10.1111/bjd.15364 28150889

[B137] Gilbert S. M. Oliphant C. J. Hassan S. Peille A. L. Bronsert P. Falzoni S. (2019). ATP in the tumour microenvironment drives expression of nfP2X7, a key mediator of cancer cell survival. Oncogene 38 (2), 194–208. 10.1038/s41388-018-0426-6 30087439 PMC6328436

[B138] Giuliani A. L. Colognesi D. Ricco T. Roncato C. Capece M. Amoroso F. (2014). Trophic activity of human P2X7 receptor isoforms A and B in osteosarcoma. PloS One 9 (9), e107224. 10.1371/journal.pone.0107224 25226385 PMC4165768

[B139] Gomez-Villafuertes R. Garcia-Huerta P. Diaz-Hernandez J. I. Miras-Portugal M. T. (2015). PI3K/Akt signaling pathway triggers P2X7 receptor expression as a pro-survival factor of neuroblastoma cells under limiting growth conditions. Sci. Rep. 5, 18417. 10.1038/srep18417 26687764 PMC4685307

[B140] Gonnord P. Delarasse C. Auger R. Benihoud K. Prigent M. Cuif M. H. (2009). Palmitoylation of the P2X7 receptor, an ATP-gated channel, controls its expression and association with lipid rafts. FASEB J. 23 (3), 795–805. 10.1096/fj.08-114637 18971257

[B141] Gopalakrishnan V. Helmink B. A. Spencer C. N. Reuben A. Wargo J. A. (2018). The Influence of the Gut Microbiome on Cancer, Immunity, and Cancer Immunotherapy. Cancer Cell 33 (4), 570–580. 10.1016/j.ccell.2018.03.015 29634945 PMC6529202

[B142] Graziano F. Desdouits M. Garzetti L. Podini P. Alfano M. Rubartelli A. (2015). Extracellular ATP induces the rapid release of HIV-1 from virus containing compartments of human macrophages. Proc. Natl. Acad. Sci. U. S. A 112 (25), E3265–E3273. 10.1073/pnas.1500656112 26056317 PMC4485148

[B143] Greig A. V. Linge C. Healy V. Lim P. Clayton E. Rustin M. H. (2003). Expression of purinergic receptors in non-melanoma skin cancers and their functional roles in A431 cells. J. Invest. Dermatol. 121 (2), 315–327. 10.1046/j.1523-1747.2003.12379.x 12880424

[B144] Grol M. W. Pereverzev A. Sims S. M. Dixon S. J. (2013). P2 receptor networks regulate signaling duration over a wide dynamic range of ATP concentrations. J. Cell Sci. 126 (Pt 16), 3615–3626. 10.1242/jcs.122705 23750003

[B145] Gu B. J. Wiley J. S. (2006). Rapid ATP-induced release of matrix metalloproteinase 9 is mediated by the P2X7 receptor. Blood 107 (12), 4946–4953. 10.1182/blood-2005-07-2994 16514055

[B146] Gu B. J. Wiley J. S. (2018). P2X7 as a scavenger receptor for innate phagocytosis in the brain. Br. J. Pharmacol. 175 (22), 4195–4208. 10.1111/bph.14470 30098011 PMC6193880

[B147] Gu B. Bendall L. J. Wiley J. S. (1998). Adenosine triphosphate-induced shedding of CD23 and L-selectin (CD62L) from lymphocytes is mediated by the same receptor but different metalloproteases. Blood 92 (3), 946–951. 10.1182/blood.V92.3.946 9680363

[B148] Gu B. J. Zhang W. Y. Bendall L. J. Chessell I. P. Buell G. N. Wiley J. S. (2000). Expression of P2X(7) purinoceptors on human lymphocytes and monocytes: evidence for nonfunctional P2X(7) receptors. Am. J. Physiol. Cell Physiol. 279 (4), C1189–C1197. 10.1152/ajpcell.2000.279.4.C1189 11003599

[B149] Gu B. J. Zhang W. Worthington R. A. Sluyter R. Dao-Ung P. Petrou S. (2001). A Glu-496 to Ala polymorphism leads to loss of function of the human P2X7 receptor. J. Biol. Chem. 276 (14), 11135–11142. 10.1074/jbc.M010353200 11150303

[B150] Gu B. J. Sluyter R. Skarratt K. K. Shemon A. N. Dao-Ung L. P. Fuller S. J. (2004). An Arg307 to Gln polymorphism within the ATP-binding site causes loss of function of the human P2X7 receptor. J. Biol. Chem. 279 (30), 31287–31295. 10.1074/jbc.M313902200 15123679

[B151] Gu B. J. Rathsam C. Stokes L. McGeachie A. B. Wiley J. S. (2009). Extracellular ATP dissociates nonmuscle myosin from P2X(7) complex: this dissociation regulates P2X(7) pore formation. Am. J. Physiol. Cell Physiol. 297 (2), C430–C439. 10.1152/ajpcell.00079.2009 19494237

[B152] Gu B. J. Saunders B. M. Jursik C. Wiley J. S. (2010). The P2X7-nonmuscle myosin membrane complex regulates phagocytosis of nonopsonized particles and bacteria by a pathway attenuated by extracellular ATP. Blood 115 (8), 1621–1631. 10.1182/blood-2009-11-251744 20007545

[B153] Gu L. Q. Li F. Y. Zhao L. Liu Y. Chu Q. Zang X. X. (2010). Association of XIAP and P2X7 receptor expression with lymph node metastasis in papillary thyroid carcinoma. Endocrine 38 (2), 276–282. 10.1007/s12020-010-9384-7 20972735

[B154] Gu B. J. Saunders B. M. Petrou S. Wiley J. S. (2011). P2X(7) is a scavenger receptor for apoptotic cells in the absence of its ligand, extracellular ATP. J. Immunol. 187 (5), 2365–2375. 10.4049/jimmunol.1101178 21821797

[B155] Gu B. J. Duce J. A. Valova V. A. Wong B. Bush A. I. Petrou S. (2012). P2X7 receptor-mediated scavenger activity of mononuclear phagocytes toward non-opsonized particles and apoptotic cells is inhibited by serum glycoproteins but remains active in cerebrospinal fluid. J. Biol. Chem. 287 (21), 17318–17330. 10.1074/jbc.M112.340885 22461619 PMC3366774

[B156] Gu B. J. Field J. Dutertre S. Ou A. Kilpatrick T. J. Lechner-Scott J. (2015). A rare P2X7 variant Arg307Gln with absent pore formation function protects against neuroinflammation in multiple sclerosis. Hum. Mol. Genet. 24 (19), 5644–5654. 10.1093/hmg/ddv278 26188005

[B157] Gudipaty L. Humphreys B. D. Buell G. Dubyak G. R. (2001). Regulation of P2X(7) nucleotide receptor function in human monocytes by extracellular ions and receptor density. Am. J. Physiol. Cell Physiol. 280 (4), C943–C953. 10.1152/ajpcell.2001.280.4.C943 11245611

[B158] Guerra A. N. Gavala M. L. Chung H. S. Bertics P. J. (2007). Nucleotide receptor signalling and the generation of reactive oxygen species. Purinerg. Signal 3 (1-2), 39–51. 10.1007/s11302-006-9035-x PMC2096761 18404417

[B159] Gulbransen B. D. Bashashati M. Hirota S. A. Gui X. Roberts J. A. MacDonald J. A. (2012). Activation of neuronal P2X7 receptor-pannexin-1 mediates death of enteric neurons during colitis. Nat. Med. 18 (4), 600–604. 10.1038/nm.2679 22426419 PMC3321107

[B160] Gunosewoyo H. Kassiou M. (2010). P2X purinergic receptor ligands: recently patented compounds. Expert Opin. Ther. Pat. 20 (5), 625–646. 10.1517/13543771003702424 20205618

[B161] Guo C. Masin M. Qureshi O. S. Murrell-Lagnado R. D. (2007). Evidence for functional P2X4/P2X7 heteromeric receptors. Mol. Pharmacol. 72 (6), 1447–1456. 10.1124/mol.107.035980 17785580

[B162] Gutierrez-Martin Y. Bustillo D. Gomez-Villafuertes R. Sanchez-Nogueiro J. Torregrosa-Hetland C. Binz T. (2011). P2X7 receptors trigger ATP exocytosis and modify secretory vesicle dynamics in neuroblastoma cells. J. Biol. Chem. 286 (13), 11370–11381. 10.1074/jbc.M110.139410 21292765 PMC3064193

[B163] Hale J. S. Youngblood B. Latner D. R. Mohammed A. U. Ye L. Akondy R. S. (2013). Distinct memory CD4+ T cells with commitment to T follicular helper- and T helper 1-cell lineages are generated after acute viral infection. Immunity 38 (4), 805–817. 10.1016/j.immuni.2013.02.020 23583644 PMC3741679

[B164] Han J. Liu H. Liu C. Jin H. Perlmutter J. S. Egan T. M. (2017). Pharmacologic characterizations of a P2X7 receptor-specific radioligand, [11C]GSK1482160 for neuroinflammatory response. Nucl. Med. Commun. 38 (5), 372–382. 10.1097/MNM.0000000000000660 28338530 PMC5401628

[B165] Hansen M. A. Barden J. A. Balcar V. J. Keay K. A. Bennett M. R. (1997). Structural motif and characteristics of the extracellular domain of P2X receptors. Biochem. Biophys. Res. Commun. 236 (3), 670–675. 10.1006/bbrc.1997.6815 9245711

[B166] Harkat M. Peverini L. Cerdan A. H. Dunning K. Beudez J. Martz A. (2017). On the permeation of large organic cations through the pore of ATP-gated P2X receptors. Proc. Natl. Acad. Sci. U. S. A 114 (19), E3786–E3E95. 10.1073/pnas.1701379114 28442564 PMC5441707

[B167] Hattori M. Gouaux E. (2012). Molecular mechanism of ATP binding and ion channel activation in P2X receptors. Nature 485 (7397), 207–212. 10.1038/nature11010 22535247 PMC3391165

[B168] Hattori F. Ohshima Y. Seki S. Tsukimoto M. Sato M. Takenouchi T. (2012). Feasibility study of B16 melanoma therapy using oxidized ATP to target purinergic receptor P2X7. Eur. J. Pharmacol. 695 (1-3), 20–26. 10.1016/j.ejphar.2012.09.001 22981895

[B169] Hibell A. D. Thompson K. M. Simon J. Xing M. Humphrey P. P. Michel A. D. (2001). Species- and agonist-dependent differences in the deactivation-kinetics of P2X7 receptors. Naunyn Schmiedebergs Arch. Pharmacol. 363 (6), 639–648. 10.1007/s002100100412 11414659

[B170] Hickman S. E. Khoury J. Greenberg S. Schieren I. Silverstein S. C. (1994). P2Z adenosine triphosphate receptor activity in cultured human monocyte-derived macrophages. Blood 84 (8), 2452–2456. 10.1182/blood.V84.8.2452.2452 7919365

[B171] Honore P. Donnelly-Roberts D. Namovic M. T. Hsieh G. Zhu C. Z. Mikusa J. P. (2006). A-740003 [N-(1-{[(cyanoimino)(5-quinolinylamino) methyl]amino}-2,2-dimethylpropyl)-2-(3,4-dimethoxyphenyl)acetamide], a novel and selective P2X7 receptor antagonist, dose-dependently reduces neuropathic pain in the rat. J. Pharmacol. Exp. Ther. 319 (3), 1376–1385. 10.1124/jpet.106.111559 16982702

[B172] Honore P. Donnelly-Roberts D. Namovic M. Zhong C. Wade C. Chandran P. (2009). The antihyperalgesic activity of a selective P2X7 receptor antagonist, A-839977, is lost in IL-1alphabeta knockout mice. Behav. Brain Res. 204 (1), 77–81. 10.1016/j.bbr.2009.05.018 19464323

[B173] Hou Z. Cao J. (2016). Comparative study of the P2X gene family in animals and plants. Purinerg. Signal 12 (2), 269–281. 10.1007/s11302-016-9501-z PMC4854843 26874702

[B174] Hu J. Ye F. Cui M. Lee P. Wei C. Hao Y. (2016). Protein Profiling of Bladder Urothelial Cell Carcinoma. PloS One 11 (9), e0161922. 10.1371/journal.pone.0161922 27626805 PMC5023150

[B175] Huang S. Chen Y. Wu W. Ouyang N. Chen J. Li H. (2013). miR-150 promotes human breast cancer growth and malignant behavior by targeting the pro-apoptotic purinergic P2X7 receptor. PloS One 8 (12), e80707. 10.1371/journal.pone.0080707 24312495 PMC3846619

[B176] Humphreys B. D. Dubyak G. R. (1996). Induction of the P2z/P2X7 nucleotide receptor and associated phospholipase D activity by lipopolysaccharide and IFN-gamma in the human THP-1 monocytic cell line. J. Immunol. 157 (12), 5627–5637. 8955215

[B177] Humphreys B. D. Rice J. Kertesy S. B. Dubyak G. R. (2000). Stress-activated protein kinase/JNK activation and apoptotic induction by the macrophage P2X7 nucleotide receptor. J. Biol. Chem. 275 (35), 26792–26798. 10.1074/jbc.M002770200 10854431

[B178] Huo H. Fryatt A. G. Farmer L. K. Schmid R. Evans R. J. (2018). Mapping the binding site of the P2X receptor antagonist PPADS reveals the importance of orthosteric site charge and the cysteine-rich head region. J. Biol. Chem. 293 (33), 12820–12831. 10.1074/jbc.RA118.003737 29997254 PMC6102130

[B179] Idzko M. Dichmann S. Ferrari D. Di Virgilio F. la Sala A. Girolomoni G. (2002). Nucleotides induce chemotaxis and actin polymerization in immature but not mature human dendritic cells via activation of pertussis toxin-sensitive P2y receptors. Blood 100 (3), 925–932. 10.1182/blood.v100.3.925 12130504

[B180] Jacobson K. A. Muller C. E. (2016). Medicinal chemistry of adenosine, P2Y and P2X receptors. Neuropharmacology 104, 31–49. 10.1016/j.neuropharm.2015.12.001 26686393 PMC4871727

[B181] Jacobson K. A. Jarvis M. F. Williams M. (2002). Purine and pyrimidine (P2) receptors as drug targets. J. Med. Chem. 45 (19), 4057–4093. 10.1021/jm020046y 12213051 PMC12443034

[B182] Janks L. Sharma C. V. R. Egan T. M. (2018). A central role for P2X7 receptors in human microglia. J. Neuroinflammation 15 (1), 325. 10.1186/s12974-018-1353-8 30463629 PMC6247771

[B183] Jarvis M. F. Khakh B. S. (2009). ATP-gated P2X cation-channels. Neuropharmacology 56 (1), 208–215. 10.1016/j.neuropharm.2008.06.067 18657557

[B184] Jelassi B. Chantome A. Alcaraz-Perez F. Baroja-Mazo A. Cayuela M. L. Pelegrin P. (2011). P2X(7) receptor activation enhances SK3 channels- and cystein cathepsin-dependent cancer cells invasiveness. Oncogene 30 (18), 2108–2122. 10.1038/onc.2010.593 21242969

[B185] Jelassi B. Anchelin M. Chamouton J. Cayuela M. L. Clarysse L. Li J. (2013). Anthraquinone emodin inhibits human cancer cell invasiveness by antagonizing P2X7 receptors. Carcinogenesis 34 (7), 1487–1496. 10.1093/carcin/bgt099 23524196

[B186] Ji Z. Xie Y. Guan Y. Zhang Y. Cho K. S. Ji M. (2018). Involvement of P2X7 Receptor in Proliferation and Migration of Human Glioma Cells. BioMed. Res. Int. 2018, 8591397. 10.1155/2018/8591397 29546069 PMC5818963

[B187] Jiang L. H. Baldwin J. M. Roger S. Baldwin S. A. (2013). Insights into the Molecular Mechanisms Underlying Mammalian P2X7 Receptor Functions and Contributions in Diseases, Revealed by Structural Modeling and Single Nucleotide Polymorphisms. Front. Pharmacol. 4, 55. 10.3389/fphar.2013.00055 23675347 PMC3646254

[B188] Jorgensen N. R. Husted L. B. Skarratt K. K. Stokes L. Tofteng C. L. Kvist T. (2012). Single-nucleotide polymorphisms in the P2X7 receptor gene are associated with post-menopausal bone loss and vertebral fractures. Eur. J. Hum. Genet. 20 (6), 675–681. 10.1038/ejhg.2011.253 22274585 PMC3355253

[B189] Kaczmarek-Hajek K. Lorinczi E. Hausmann R. Nicke A. (2012). Molecular and functional properties of P2X receptors–recent progress and persisting challenges. Purinerg. Signal 8 (3), 375–417. 10.1007/s11302-012-9314-7 PMC3360091 22547202

[B190] Kaiho H. Matsuoka I. Kimura J. Nakanishi H. (1998). Identification of P2X7 (P2Z) receptor in N18TG-2 cells and NG108-15 cells. J. Neurochem. 70 (3), 951–957. 10.1046/j.1471-4159.1998.70030951.x 9489714

[B191] Kan L. K. Williams D. Drummond K. O’Brien T. Monif M. (2019). The role of microglia and P2X7 receptors in gliomas. J. Neuroimmunol. 332, 138–146. 10.1016/j.jneuroim.2019.04.010 31031209

[B192] Kang R. Zeh H. Lotze M. Tang D. (2020). The Multifaceted Effects of Autophagy on the Tumor Microenvironment. Adv. Exp. Med. Biol. 1225, 99–114. 10.1007/978-3-030-35727-6_7 32030650

[B193] Karasawa A. Kawate T. (2016). Structural basis for subtype-specific inhibition of the P2X7 receptor. Elife 5. 10.7554/eLife.22153 PMC5176352 27935479

[B194] Karasawa A. Michalski K. Mikhelzon P. Kawate T. (2017). The P2X7 receptor forms a dye-permeable pore independent of its intracellular domain but dependent on membrane lipid composition. Elife 6. 10.7554/eLife.31186 PMC5624784 28920575

[B195] Kasuya G. Fujiwara Y. Tsukamoto H. Morinaga S. Ryu S. Touhara K. (2017). Structural insights into the nucleotide base specificity of P2X receptors. Sci. Rep. 7, 45208. 10.1038/srep45208 28332633 PMC5362899

[B196] Kasuya G. Yamaura T. Ma X. B. Nakamura R. Takemoto M. Nagumo H. (2017). Structural insights into the competitive inhibition of the ATP-gated P2X receptor channel. Nat. Commun. 8 (1), 876. 10.1038/s41467-017-00887-9 29026074 PMC5638823

[B197] Kataoka A. Tozaki-Saitoh H. Koga Y. Tsuda M. Inoue K. (2009). Activation of P2X7 receptors induces CCL3 production in microglial cells through transcription factor NFAT. J. Neurochem. 108 (1), 115–125. 10.1111/j.1471-4159.2008.05744.x 19014371

[B198] Kawate T. Michel J. C. Birdsong W. T. Gouaux E. (2009). Crystal structure of the ATP-gated P2X(4) ion channel in the closed state. Nature 460 (7255), 592–598. 10.1038/nature08198 19641588 PMC2720809

[B199] Keystone E. C. Wang M. M. Layton M. Hollis S. McInnes I. B. Team D. C. S. (2012). Clinical evaluation of the efficacy of the P2X7 purinergic receptor antagonist AZD9056 on the signs and symptoms of rheumatoid arthritis in patients with active disease despite treatment with methotrexate or sulphasalazine. Ann. Rheum. Dis. 71 (10), 1630–1635. 10.1136/annrheumdis-2011-143578 22966146

[B200] Khakh B. S. Lester H. A. (1999). Dynamic selectivity filters in ion channels. Neuron 23 (4), 653–658. 10.1016/s0896-6273(01)80025-8 10482233

[B201] Kim M. Jiang L. H. Wilson H. L. North R. A. Surprenant A. (2001). Proteomic and functional evidence for a P2X7 receptor signalling complex. EMBO J. 20 (22), 6347–6358. 10.1093/emboj/20.22.6347 11707406 PMC125721

[B202] Kopp R. Krautloher A. Ramirez-Fernandez A. Nicke A. (2019). P2X7 Interactions and Signaling - Making Head or Tail of It. Front. Mol. Neurosci. 12, 183. 10.3389/fnmol.2019.00183 31440138 PMC6693442

[B203] Koshimizu T. Koshimizu M. Stojilkovic S. S. (1999). Contributions of the C-terminal domain to the control of P2X receptor desensitization. J. Biol. Chem. 274 (53), 37651–37657. 10.1074/jbc.274.53.37651 10608821

[B204] Kuehnel M. P. Rybin V. Anand P. K. Anes E. Griffiths G. (2009). Lipids regulate P2X7-receptor-dependent actin assembly by phagosomes via ADP translocation and ATP synthesis in the phagosome lumen. J. Cell Sci. 122 (Pt 4), 499–504. 10.1242/jcs.034199 19174471

[B205] Kunzli B. M. Berberat P. O. Giese T. Csizmadia E. Kaczmarek E. Baker C. (2007). Upregulation of CD39/NTPDases and P2 receptors in human pancreatic disease. Am. J. Physiol. Gastrointest. Liver Physiol. 292 (1), G223–G230. 10.1152/ajpgi.00259.2006 16920697

[B206] Kunzli B. M. Bernlochner M. I. Rath S. Kaser S. Csizmadia E. Enjyoji K. (2011). Impact of CD39 and purinergic signalling on the growth and metastasis of colorectal cancer. Purinerg. Signal 7 (2), 231–241. 10.1007/s11302-011-9228-9 PMC3146639 21484085

[B207] Kurashima Y. Amiya T. Nochi T. Fujisawa K. Haraguchi T. Iba H. (2012). Extracellular ATP mediates mast cell-dependent intestinal inflammation through P2X7 purinoceptors. Nat. Commun. 3, 1034. 10.1038/ncomms2023 22948816 PMC3658010

[B208] Kwon J. H. Nam E. S. Shin H. S. Cho S. J. Park H. R. Kwon M. J. (2014). P2X7 Receptor Expression in Coexistence of Papillary Thyroid Carcinoma with Hashimoto’s Thyroiditis. Korean J. Pathol. 48 (1), 30–35. 10.4132/KoreanJPathol.2014.48.1.30 24627692 PMC3950232

[B209] Lawrence M. S. Stojanov P. Mermel C. H. Robinson J. T. Garraway L. A. Golub T. R. (2014). Discovery and saturation analysis of cancer genes across 21 tumour types. Nature 505 (7484), 495–501. 10.1038/nature12912 24390350 PMC4048962

[B210] Ledderose C. Woehrle T. Ledderose S. Strasser K. Seist R. Bao Y. (2016). Cutting off the power: inhibition of leukemia cell growth by pausing basal ATP release and P2X receptor signaling? Purinerg. Signal 12 (3), 439–451. 10.1007/s11302-016-9510-y PMC5023625 27020575

[B211] Lenertz L. Y. Gavala M. L. Hill L. M. Bertics P. J. (2009). Cell signaling via the P2X(7) nucleotide receptor: linkage to ROS production, gene transcription, and receptor trafficking. Purinerg. Signal 5 (2), 175–187. 10.1007/s11302-009-9133-7 PMC2686823 19263245

[B212] Lenertz L. Y. Wang Z. Guadarrama A. Hill L. M. Gavala M. L. Bertics P. J. (2010). Mutation of putative N-linked glycosylation sites on the human nucleotide receptor P2X7 reveals a key residue important for receptor function. Biochemistry 49 (22), 4611–4619. 10.1021/bi902083n 20450227 PMC2895974

[B213] Li X. Zhou L. Feng Y. H. Abdul-Karim F. W. Gorodeski G. I. (2006). The P2X7 receptor: a novel biomarker of uterine epithelial cancers. Cancer Epidemiol. Biomarkers Prev. 15 (10), 1906–1913. 10.1158/1055-9965.EPI-06-0407 17035398 PMC2376759

[B214] Li X. Qi X. Zhou L. Catera D. Rote N. S. Potashkin J. (2007). Decreased expression of P2X7 in endometrial epithelial pre-cancerous and cancer cells. Gynecol. Oncol. 106 (1), 233–243. 10.1016/j.ygyno.2007.03.032 17482244 PMC2398694

[B215] Li X. Qi X. Zhou L. Fu W. Abdul-Karim F. W. Maclennan G. (2009). P2X(7) receptor expression is decreased in epithelial cancer cells of ectodermal, uro-genital sinus, and distal paramesonephric duct origin. Purinerg. Signal 5 (3), 351–368. 10.1007/s11302-009-9161-3 PMC2717318 19399640

[B216] Li M. Toombes G. E. Silberberg S. D. Swartz K. J. (2015). Physical basis of apparent pore dilation of ATP-activated P2X receptor channels. Nat. Neurosci. 18 (11), 1577–1583. 10.1038/nn.4120 26389841 PMC5113834

[B217] Li X. Y. Moesta A. K. Xiao C. Nakamura K. Casey M. Zhang H. (2019). Targeting CD39 in Cancer Reveals an Extracellular ATP- and Inflammasome-Driven Tumor Immunity. Cancer Discovery 9 (12), 1754–1773. 10.1158/2159-8290.CD-19-0541 31699796 PMC6891207

[B218] Liang X. Samways D. S. K. Cox J. Egan T. M. (2019). Ca(2+) flux through splice variants of the ATP-gated ionotropic receptor P2X7 is regulated by its cytoplasmic N terminus. J. Biol. Chem. 294 (33), 12521–12533. 10.1074/jbc.RA119.009666 31248985 PMC6699846

[B219] Liu P. S. Chen C. Y. (2010). Butyl benzyl phthalate suppresses the ATP-induced cell proliferation in human osteosarcoma HOS cells. Toxicol. Appl. Pharmacol. 244 (3), 308–314. 10.1016/j.taap.2010.01.007 20114058

[B220] Liu Y. Xiao Y. Li Z. (2011). P2X7 receptor positively regulates MyD88-dependent NF-kappaB activation. Cytokine 55 (2), 229–236. 10.1016/j.cyto.2011.05.003 21621419

[B221] Liu Z. Liu Y. Xu L. An H. Chang Y. Yang Y. (2015). P2X7 receptor predicts postoperative cancer-specific survival of patients with clear-cell renal cell carcinoma. Cancer Sci. 106 (9), 1224–1231. 10.1111/cas.12736 26179886 PMC4582993

[B222] Lopez-Castejon G. Brough D. (2011). Understanding the mechanism of IL-1beta secretion. Cytokine Growth Factor Rev. 22 (4), 189–195. 10.1016/j.cytogfr.2011.10.001 22019906 PMC3714593

[B223] Lord B. Aluisio L. Shoblock J. R. Neff R. A. Varlinskaya E. I. Ceusters M. (2014). Pharmacology of a novel central nervous system-penetrant P2X7 antagonist JNJ-42253432. J. Pharmacol. Exp. Ther. 351 (3), 628–641. 10.1124/jpet.114.218487 25271258

[B224] Lord B. Ameriks M. K. Wang Q. Fourgeaud L. Vliegen M. Verluyten W. (2015). A novel radioligand for the ATP-gated ion channel P2X7: [3H] JNJ-54232334. Eur. J. Pharmacol. 765, 551–559. 10.1016/j.ejphar.2015.09.026 26386289

[B225] Ma Y. Adjemian S. Yang H. Catani J. P. Hannani D. Martins I. (2013). ATP-dependent recruitment, survival and differentiation of dendritic cell precursors in the tumor bed after anticancer chemotherapy. Oncoimmunology 2 (6), e24568. 10.4161/onci.24568 23894718 PMC3716753

[B226] MacKenzie A. Wilson H. L. Kiss-Toth E. Dower S. K. North R. A. Surprenant A. (2001). Rapid secretion of interleukin-1beta by microvesicle shedding. Immunity 15 (5), 825–835. 10.1016/s1074-7613(01)00229-1 11728343

[B227] Mackenzie A. B. Young M. T. Adinolfi E. Surprenant A. (2005). Pseudoapoptosis induced by brief activation of ATP-gated P2X7 receptors. J. Biol. Chem. 280 (40), 33968–33976. 10.1074/jbc.M502705200 15994333

[B228] Maianski Z. Pedersen J. Chabert C. Barden J. S P. D. (2007). 1435: Evaluation of a New Monoclonal Antibody Targeting the Apoptotic Purinergic Receptor P2x7, as a Diagnostic Tool for Prostate Cancer. J. Urol. 177 (4S), 474. 10.1016/S0022-5347(18)31636-7

[B229] Mansoor S. E. Lu W. Oosterheert W. Shekhar M. Tajkhorshid E. Gouaux E. (2016). X-ray structures define human P2X(3) receptor gating cycle and antagonist action. Nature 538 (7623), 66–71. 10.1038/nature19367 27626375 PMC5161641

[B230] Martel-Gallegos G. Casas-Pruneda G. Ortega-Ortega F. Sanchez-Armass S. Olivares-Reyes J. A. Diebold B. (2013). Oxidative stress induced by P2X7 receptor stimulation in murine macrophages is mediated by c-Src/Pyk2 and ERK1/2. Biochim. Biophys. Acta 1830 (10), 4650–4659. 10.1016/j.bbagen.2013.05.023 23711511

[B231] Mawatwal S. Behura A. Ghosh A. Kidwai S. Mishra A. Deep A. (2017). Calcimycin mediates mycobacterial killing by inducing intracellular calcium-regulated autophagy in a P2RX7 dependent manner. Biochim. Biophys. Acta Gen. Subj. 1861 (12), 3190–3200. 10.1016/j.bbagen.2017.09.010 28935606

[B232] McCarthy A. E. Yoshioka C. Mansoor S. E. (2019). Full-Length P2X7 Structures Reveal How Palmitoylation Prevents Channel Desensitization. Cell. 179 (3), 659–670.e13. 10.1016/j.cell.2019.09.017 PMC7053488 31587896

[B233] McInnes I. B. Cruwys S. Bowers K. Braddock M. (2014). Targeting the P2X7 receptor in rheumatoid arthritis: biological rationale for P2X7 antagonism. Clin. Exp. Rheumatol. 32 (6), 878–882. 25288220

[B234] McLarnon J. G. (2017). Roles of purinergic P2X7 receptor in glioma and microglia in brain tumors. Cancer Lett. 402, 93–99. 10.1016/j.canlet.2017.05.004 28536012

[B235] Mehta N. Kaur M. Singh M. Chand S. Vyas B. Silakari P. (2014). Purinergic receptor P2X(7): a novel target for anti-inflammatory therapy. Bioorg. Med. Chem. 22 (1), 54–88. 10.1016/j.bmc.2013.10.054 24314880

[B236] Michaud M. Martins I. Sukkurwala A. Q. Adjemian S. Ma Y. Pellegatti P. (2011). Autophagy-dependent anticancer immune responses induced by chemotherapeutic agents in mice. Science 334 (6062), 1573–1577. 10.1126/science.1208347 22174255

[B237] Michel A. D. Fonfria E. (2007). Agonist potency at P2X7 receptors is modulated by structurally diverse lipids. Br. J. Pharmacol. 152 (4), 523–537. 10.1038/sj.bjp.0707417 17700717 PMC2050815

[B238] Michel A. D. Kaur R. Chessell I. P. Humphrey P. P. (2000). Antagonist effects on human P2X(7) receptor-mediated cellular accumulation of YO-PRO-1. Br. J. Pharmacol. 130 (3), 513–520. 10.1038/sj.bjp.0703368 10821778 PMC1572117

[B239] Michel A. D. Chambers L. J. Walter D. S. (2008a). Negative and positive allosteric modulators of the P2X(7) receptor. Br. J. Pharmacol. 153 (4), 737–750. 10.1038/sj.bjp.0707625 18071294 PMC2259211

[B240] Michel A. D. Clay W. C. Ng S. W. Roman S. Thompson K. Condreay J. P. (2008b). Identification of regions of the P2X(7) receptor that contribute to human and rat species differences in antagonist effects. Br. J. Pharmacol. 155 (5), 738–751. 10.1038/bjp.2008.306 18660826 PMC2584934

[B241] Michelsen K. Yuan H. Schwappach B. (2005). Hide and run. Arginine-based endoplasmic-reticulum-sorting motifs in the assembly of heteromultimeric membrane proteins. EMBO Rep. 6 (8), 717–722. 10.1038/sj.embor.7400480 16065065 PMC1369147

[B242] Middaugh C. R. Mach H. Burke C. J. Volkin D. B. Dabora J. M. Tsai P. K. (1992). Nature of the interaction of growth factors with suramin. Biochemistry 31 (37), 9016–9024. 10.1021/bi00152a044 1390688

[B243] Minkiewicz J. de Rivero Vaccari J. P. Keane R. W. (2013). Human astrocytes express a novel NLRP2 inflammasome. Glia 61 (7), 1113–1121. 10.1002/glia.22499 23625868

[B244] Mistafa O. Stenius U. (2009). Statins inhibit Akt/PKB signaling via P2X7 receptor in pancreatic cancer cells. Biochem. Pharmacol. 78 (9), 1115–1126. 10.1016/j.bcp.2009.06.016 19540829

[B245] Moncao-Ribeiro L. C. Faffe D. S. Santana P. T. Vieira F. S. da Graca C. L. Marques-da-Silva C. (2014). P2X7 receptor modulates inflammatory and functional pulmonary changes induced by silica. PloS One 9 (10), e110185. 10.1371/journal.pone.0110185 25310682 PMC4195726

[B246] Monif M. Reid C. A. Powell K. L. Smart M. L. Williams D. A. (2009). The P2X7 receptor drives microglial activation and proliferation: a trophic role for P2X7R pore. J. Neurosci. 29 (12), 3781–3791. 10.1523/JNEUROSCI.5512-08.2009 19321774 PMC6665035

[B247] Moore S. F. MacKenzie A. B. (2007). Murine macrophage P2X7 receptors support rapid prothrombotic responses. Cell Signal 19 (4), 855–866. 10.1016/j.cellsig.2006.10.010 17175137

[B248] Moore S. F. MacKenzie A. B. (2009). NADPH oxidase NOX2 mediates rapid cellular oxidation following ATP stimulation of endotoxin-primed macrophages. J. Immunol. 183 (5), 3302–3308. 10.4049/jimmunol.0900394 19696433

[B249] Morciano G. Sarti A. C. Marchi S. Missiroli S. Falzoni S. Raffaghello L. (2017). Use of luciferase probes to measure ATP in living cells and animals. Nat. Protoc. 12 (8), 1542–1562. 10.1038/nprot.2017.052 28683062

[B250] Morelli A. Chiozzi P. Chiesa A. Ferrari D. Sanz J. M. Falzoni S. (2003). Extracellular ATP causes ROCK I-dependent bleb formation in P2X7-transfected HEK293 cells. Mol. Biol. Cell 14 (7), 2655–2664. 10.1091/mbc.02-04-0061 12857854 PMC165666

[B251] Mulcahy Levy J. M. Thorburn A. (2020). Autophagy in cancer: moving from understanding mechanism to improving therapy responses in patients. Cell Death Differ. 27 (3), 843–857. 10.1038/s41418-019-0474-7 31836831 PMC7206017

[B252] Munoz-Planillo R. Kuffa P. Martinez-Colon G. Smith B. L. Rajendiran T. M. Nunez G. (2013). K(+) efflux is the common trigger of NLRP3 inflammasome activation by bacterial toxins and particulate matter. Immunity 38 (6), 1142–1153. 10.1016/j.immuni.2013.05.016 23809161 PMC3730833

[B253] Mutini C. Falzoni S. Ferrari D. Chiozzi P. Morelli A. Baricordi O. R. (1999). Mouse dendritic cells express the P2X7 purinergic receptor: characterization and possible participation in antigen presentation. J. Immunol. 163 (4), 1958–1965. 10438932

[B254] Nelson D. W. Gregg R. J. Kort M. E. Perez-Medrano A. Voight E. A. Wang Y. (2006). Structure-activity relationship studies on a series of novel, substituted 1-benzyl-5-phenyltetrazole P2X7 antagonists. J. Med. Chem. 49 (12), 3659–3666. 10.1021/jm051202e 16759108

[B255] Noguchi T. Ishii K. Fukutomi H. Naguro I. Matsuzawa A. Takeda K. (2008). Requirement of reactive oxygen species-dependent activation of ASK1-p38 MAPK pathway for extracellular ATP-induced apoptosis in macrophage. J. Biol. Chem. 283 (12), 7657–7665. 10.1074/jbc.M708402200 18211888

[B256] North R. A. Surprenant A. (2000). Pharmacology of cloned P2X receptors. Annu. Rev. Pharmacol. Toxicol. 40, 563–580. 10.1146/annurev.pharmtox.40.1.563 10836147

[B257] North R. A. (2002). Molecular physiology of P2X receptors. Physiol. Rev. 82 (4), 1013–1067. 10.1152/physrev.00015.2002 12270951

[B258] Orioli E. De Marchi E. Giuliani A. L. Adinolfi E. (2017). P2X7 receptor orchestrates multiple signalling pathways triggering inflammation, autophagy and metabolic/trophic responses. Curr. Med. Chem. 24 (21), 2261–2275. 10.2174/0929867324666170303161659 28266268

[B259] Overes I. M. de Rijke B. van Horssen-Zoetbrood A. Fredrix H. de Graaf A. O. Jansen J. H. (2008). Expression of P2X5 in lymphoid malignancies results in LRH-1-specific cytotoxic T-cell-mediated lysis. Br. J. Haematol. 141 (6), 799–807. 10.1111/j.1365-2141.2008.07125.x 18410452

[B260] Oyanguren-Desez O. Rodriguez-Antiguedad A. Villoslada P. Domercq M. Alberdi E. Matute C. (2011). Gain-of-function of P2X7 receptor gene variants in multiple sclerosis. Cell Calcium 50 (5), 468–472. 10.1016/j.ceca.2011.08.002 21906809

[B261] Pandolfi J. B. Ferraro A. A. Sananez I. Gancedo M. C. Baz P. Billordo L. A. (2016). ATP-Induced Inflammation Drives Tissue-Resident Th17 Cells in Metabolically Unhealthy Obesity. J. Immunol. 196 (8), 3287–3296. 10.4049/jimmunol.1502506 26951799

[B262] Panenka W. Jijon H. Herx L. M. Armstrong J. N. Feighan D. Wei T. (2001). P2X7-like receptor activation in astrocytes increases chemokine monocyte chemoattractant protein-1 expression via mitogen-activated protein kinase. J. Neurosci. 21 (18), 7135–7142. 10.1523/JNEUROSCI.21-18-07135.2001 11549724 PMC6762971

[B263] Panupinthu N. Rogers J. T. Zhao L. Solano-Flores L. P. Possmayer F. Sims S. M. (2008). P2X7 receptors on osteoblasts couple to production of lysophosphatidic acid: a signaling axis promoting osteogenesis. J. Cell Biol. 181 (5), 859–871. 10.1083/jcb.200708037 18519738 PMC2396816

[B264] Park J. H. Kim Y. C. (2017). P2X7 receptor antagonists: a patent review (2010-2015). Expert Opin. Ther. Pat. 27 (3), 257–267. 10.1080/13543776.2017.1246538 27724045

[B265] Park J. H. Williams D. R. Lee J. H. Lee S. D. Lee J. H. Ko H. (2016). Potent Suppressive Effects of 1-Piperidinylimidazole Based Novel P2X7 Receptor Antagonists on Cancer Cell Migration and Invasion. J. Med. Chem. 59 (16), 7410–7430. 10.1021/acs.jmedchem.5b01690 27427902

[B266] Park M. Kim J. Phuong N. T. T. Park J. G. Park J. H. Kim Y. C. (2019). Involvement of the P2X7 receptor in the migration and metastasis of tamoxifen-resistant breast cancer: effects on small extracellular vesicles production. Sci. Rep. 9 (1), 11587. 10.1038/s41598-019-47734-z 31406126 PMC6690963

[B267] Parvathenani L. K. Tertyshnikova S. Greco C. R. Roberts S. B. Robertson B. Posmantur R. (2003). P2X7 mediates superoxide production in primary microglia and is up-regulated in a transgenic mouse model of Alzheimer’s disease. J. Biol. Chem. 278 (15), 13309–13317. 10.1074/jbc.M209478200 12551918

[B268] Patel C. H. Powell J. D. (2017). Targeting T cell metabolism to regulate T cell activation, differentiation and function in disease. Curr. Opin. Immunol. 46, 82–88. 10.1016/j.coi.2017.04.006 28521236 PMC5554728

[B269] Pegoraro A. Bortolotti D. Marci R. Caselli E. Falzoni S. De Marchi E. (2020). The P2X7 Receptor 489C>T Gain of Function Polymorphism Favors HHV-6A Infection and Associates With Female Idiopathic Infertility. Front. Pharmacol. 11 (96). 10.3389/fphar.2020.00096. PMC7046806 32153407

[B270] Pelegrin P. Barroso-Gutierrez C. Surprenant A. (2008). P2X7 receptor differentially couples to distinct release pathways for IL-1beta in mouse macrophage. J. Immunol. 180 (11), 7147–7157. 10.4049/jimmunol.180.11.7147 18490713

[B271] Pellegatti P. Falzoni S. Pinton P. Rizzuto R. Di Virgilio F. (2005). A novel recombinant plasma membrane-targeted luciferase reveals a new pathway for ATP secretion. Mol. Biol. Cell 16 (8), 3659–3665. 10.1091/mbc.e05-03-0222 15944221 PMC1182305

[B272] Pellegatti P. Raffaghello L. Bianchi G. Piccardi F. Pistoia V. Di Virgilio F. (2008). Increased level of extracellular ATP at tumor sites: in vivo imaging with plasma membrane luciferase. PloS One 3 (7), e2599. 10.1371/journal.pone.0002599 18612415 PMC2440522

[B273] Peng L. Bradley C. J. Wiley J. S. (1999). P2Z purinoceptor, a special receptor for apoptosis induced by ATP in human leukemic lymphocytes. Chin. Med. J. (Engl.) 112 (4), 356–362. 11593539

[B274] Perregaux D. G. Gabel C. A. (1998). Human monocyte stimulus-coupled IL-1beta posttranslational processing: modulation via monovalent cations. Am. J. Physiol. 275 (6), C1538–C1547. 10.1152/ajpcell.1998.275.6.C1538 9843715

[B275] Perregaux D. G. McNiff P. Laliberte R. Conklyn M. Gabel C. A. (2000). ATP acts as an agonist to promote stimulus-induced secretion of IL-1 beta and IL-18 in human blood. J. Immunol. 165 (8), 4615–4623. 10.4049/jimmunol.165.8.4615 11035104

[B276] Perruzza L. Gargari G. Proietti M. Fosso B. D’Erchia A. M. Faliti C. E. (2017). T Follicular Helper Cells Promote a Beneficial Gut Ecosystem for Host Metabolic Homeostasis by Sensing Microbiota-Derived Extracellular ATP. Cell Rep. 18 (11), 2566–2575. 10.1016/j.celrep.2017.02.061 28297661 PMC5368345

[B277] Pevarello P. Bovolenta S. Tarroni P. Za L. Severi E. Torino D. (2017). P2X7 antagonists for CNS indications: recent patent disclosures. Pharm. Pat. Anal. 6 (2), 61–76. 10.4155/ppa-2016-0044 28248151

[B278] Piccioli P. Rubartelli A. (2013). The secretion of IL-1beta and options for release. Semin. Immunol. 25 (6), 425–429. 10.1016/j.smim.2013.10.007 24201029

[B279] Pietrocola F. Pol J. Vacchelli E. Rao S. Enot D. P. Baracco E. E. (2016). Caloric Restriction Mimetics Enhance Anticancer Immunosurveillance. Cancer Cell 30 (1), 147–160. 10.1016/j.ccell.2016.05.016 27411589 PMC5715805

[B280] Pippel A. Stolz M. Woltersdorf R. Kless A. Schmalzing G. Markwardt F. (2017). Localization of the gate and selectivity filter of the full-length P2X7 receptor. Proc. Natl. Acad. Sci. U. S. A 114 (11), E2156–E2E65. 10.1073/pnas.1610414114 28235784 PMC5358401

[B281] Pizzirani C. Ferrari D. Chiozzi P. Adinolfi E. Sandona D. Savaglio E. (2007). Stimulation of P2 receptors causes release of IL-1beta-loaded microvesicles from human dendritic cells. Blood 109 (9), 3856–3864. 10.1182/blood-2005-06-031377 17192399

[B282] Proietti M. Perruzza L. Scribano D. Pellegrini G. D’Antuono R. Strati F. (2019). ATP released by intestinal bacteria limits the generation of protective IgA against enteropathogens. Nat. Commun. 10 (1), 250. 10.1038/s41467-018-08156-z 30651557 PMC6335424

[B283] Qian F. Xiao J. Hu B. Sun N. Yin W. Zhu J. (2017). High expression of P2X7R is an independent postoperative indicator of poor prognosis in colorectal cancer. Hum. Pathol. 64, 61–68. 10.1016/j.humpath.2017.03.019 28412208

[B284] Qiu Y. Li W. H. Zhang H. Q. Liu Y. Tian X. X. Fang W. G. (2014). P2X7 mediates ATP-driven invasiveness in prostate cancer cells. PloS One 9 (12), e114371. 10.1371/journal.pone.0114371 25486274 PMC4259308

[B285] Qu Y. Dubyak G. R. (2009). P2X7 receptors regulate multiple types of membrane trafficking responses and non-classical secretion pathways. Purinerg. Signal 5 (2), 163–173. 10.1007/s11302-009-9132-8 PMC2686822 19189228

[B286] Qu Y. Franchi L. Nunez G. Dubyak G. R. (2007). Nonclassical IL-1 beta secretion stimulated by P2X7 receptors is dependent on inflammasome activation and correlated with exosome release in murine macrophages. J. Immunol. 179 (3), 1913–1925. 10.4049/jimmunol.179.3.1913 17641058

[B287] Raffaghello L. Chiozzi P. Falzoni S. Di Virgilio F. Pistoia V. (2006). The P2X7 receptor sustains the growth of human neuroblastoma cells through a substance P-dependent mechanism. Cancer Res. 66 (2), 907–914. 10.1158/0008-5472.CAN-05-3185 16424024

[B288] Ravenna L. Sale P. Di Vito M. Russo A. Salvatori L. Tafani M. (2009). Up-regulation of the inflammatory-reparative phenotype in human prostate carcinoma. Prostate 69 (11), 1245–1255. 10.1002/pros.20966 19444819

[B289] Reczek C. R. Chandel N. S. (2015). ROS-dependent signal transduction. Curr. Opin. Cell Biol. 33, 8–13. 10.1016/j.ceb.2014.09.010 25305438 PMC4380867

[B290] Reinhold W. C. Sunshine M. Liu H. Varma S. Kohn K. W. Morris J. (2012). CellMiner: a web-based suite of genomic and pharmacologic tools to explore transcript and drug patterns in the NCI-60 cell line set. Cancer Res. 72 (14), 3499–3511. 10.1158/0008-5472.CAN-12-1370 22802077 PMC3399763

[B291] Rizzo R. Ferrari D. Melchiorri L. Stignani M. Gulinelli S. Baricordi O. R. (2009). Extracellular ATP acting at the P2X7 receptor inhibits secretion of soluble HLA-G from human monocytes. J. Immunol. 183 (7), 4302–4311. 10.4049/jimmunol.0804265 19748989

[B292] Robert R. Carlile G. W. Pavel C. Liu N. Anjos S. M. Liao J. (2008). Structural analog of sildenafil identified as a novel corrector of the F508del-CFTR trafficking defect. Mol. Pharmacol. 73 (2), 478–489. 10.1124/mol.107.040725 17975008

[B293] Robinson L. E. Murrell-Lagnado R. D. (2013). The trafficking and targeting of P2X receptors. Front. Cell Neurosci. 7, 233. 10.3389/fncel.2013.00233 24319412 PMC3837535

[B294] Robinson L. E. Shridar M. Smith P. Murrell-Lagnado R. D. (2014). Plasma membrane cholesterol as a regulator of human and rodent P2X7 receptor activation and sensitization. J. Biol. Chem. 289 (46), 31983–31994. 10.1074/jbc.M114.574699 25281740 PMC4231676

[B295] Roger S. Mei Z. Z. Baldwin J. M. Dong L. Bradley H. Baldwin S. A. (2010). Single nucleotide polymorphisms that were identified in affective mood disorders affect ATP-activated P2X7 receptor functions. J. Psychiatr. Res. 44 (6), 347–355. 10.1016/j.jpsychires.2009.10.005 19931869

[B296] Roger S. Jelassi B. Couillin I. Pelegrin P. Besson P. Jiang L. H. (2015). Understanding the roles of the P2X7 receptor in solid tumour progression and therapeutic perspectives. Biochim. Biophys. Acta 1848 (10 Pt B), 2584–2602. 10.1016/j.bbamem.2014.10.029 25450340

[B297] Rozengurt E. Heppel L. A. (1975). A Specific effect of external ATP on the permeability of transformed 3T3 cells. Biochem. Biophys. Res. Commun. 67 (4), 1581–1588. 10.1016/0006-291x(75)90207-7 1039

[B298] Ruggeri R. M. Villari D. Simone A. Scarfi R. Attard M. Orlandi F. (2002). Co-expression of interleukin-6 (IL-6) and interleukin-6 receptor (IL-6R) in thyroid nodules is associated with co-expression of CD30 ligand/CD30 receptor. J. Endocrinol. Invest. 25 (11), 959–966. 10.1007/BF03344068 12553555

[B299] Rump A. Smolander O. P. Ruutel Boudinot S. Kanellopoulos J. M. Boudinot P. (2020). Evolutionary Origin of the P2X7 C-ter Region: Capture of an Ancient Ballast Domain by a P2X4-Like Gene in Ancient Jawed Vertebrates. Front. Immunol. 11, 113. 10.3389/fimmu.2020.00113 32117264 PMC7016195

[B300] Ryu J. K. Jantaratnotai N. Serrano-Perez M. C. McGeer P. L. McLarnon J. G. (2011). Block of purinergic P2X7R inhibits tumor growth in a C6 glioma brain tumor animal model. J. Neuropathol. Exp. Neurol. 70 (1), 13–22. 10.1097/NEN.0b013e318201d4d4 21157381

[B301] Salaro E. Rambaldi A. Falzoni S. Amoroso F. S. Franceschini A. Sarti A. C. (2016). Involvement of the P2X7-NLRP3 axis in leukemic cell proliferation and death. Sci. Rep. 6, 26280. 10.1038/srep26280 27221966 PMC4879576

[B302] Salvestrini V. Orecchioni S. Talarico G. Reggiani F. Mazzetti C. Bertolini F. (2017). Extracellular ATP induces apoptosis through P2X7R activation in acute myeloid leukemia cells but not in normal hematopoietic stem cells. Oncotarget 8 (4), 5895–5908. 10.18632/oncotarget.13927 27980223 PMC5351599

[B303] Santos A. A. Jr. Cappellari A. R. de Marchi F. O. Gehring M. P. Zaparte A. Brandao C. A. (2017). Potential role of P2X7R in esophageal squamous cell carcinoma proliferation. Purinerg. Signal 13 (3), 279–292. 10.1007/s11302-017-9559-2 PMC5563289 28397110

[B304] Sanz J. M. Di Virgilio F. (2000). Kinetics and mechanism of ATP-dependent IL-1 beta release from microglial cells. J. Immunol. 164 (9), 4893–488. 10.4049/jimmunol.164.9.4893 10779799

[B305] Savio L. E. B. de Andrade Mello P. Figliuolo V. R. de Avelar Almeida T. F. Santana P. T. Oliveira S. D. S. (2017). CD39 limits P2X7 receptor inflammatory signaling and attenuates sepsis-induced liver injury. J. Hepatol. 67 (4), 716–726. 10.1016/j.jhep.2017.05.021 28554875 PMC5875702

[B306] Schenk U. Westendorf A. M. Radaelli E. Casati A. Ferro M. Fumagalli M. (2008). Purinergic control of T cell activation by ATP released through pannexin-1 hemichannels. Sci. Signal 1 (39), ra6. 10.1126/scisignal.1160583 18827222

[B307] Schenk U. Frascoli M. Proietti M. Geffers R. Traggiai E. Buer J. (2011). ATP inhibits the generation and function of regulatory T cells through the activation of purinergic P2X receptors. Sci. Signal 4 (162), ra12. 10.1126/scisignal.2001270 21364186

[B308] Schmid S. Kubler M. Korcan Ayata C. Lazar Z. Haager B. Hossfeld M. (2015). Altered purinergic signaling in the tumor associated immunologic microenvironment in metastasized non-small-cell lung cancer. Lung Cancer 90 (3), 516–521. 10.1016/j.lungcan.2015.10.005 26505137

[B309] Schrier S. M. Florea B. I. Mulder G. J. Nagelkerke J. F. AP I. J. (2002). Apoptosis induced by extracellular ATP in the mouse neuroblastoma cell line N1E-115: studies on involvement of P2 receptors and adenosine. Biochem. Pharmacol. 63 (6), 1119–1126. 10.1016/s0006-2952(01)00939-x 11931844

[B310] Schwarz N. Drouot L. Nicke A. Fliegert R. Boyer O. Guse A. H. (2012). Alternative splicing of the N-terminal cytosolic and transmembrane domains of P2X7 controls gating of the ion channel by ADP-ribosylation. PloS One 7 (7), e41269. 10.1371/journal.pone.0041269 22848454 PMC3407210

[B311] Shankavaram U. T. Varma S. Kane D. Sunshine M. Chary K. K. Reinhold W. C. (2009). CellMiner: a relational database and query tool for the NCI-60 cancer cell lines. BMC Genomics 10, 277. 10.1186/1471-2164-10-277 19549304 PMC2709662

[B312] Shemon A. N. Sluyter R. Fernando S. L. Clarke A. L. Dao-Ung L. P. Skarratt K. K. (2006). A Thr357 to Ser polymorphism in homozygous and compound heterozygous subjects causes absent or reduced P2X7 function and impairs ATP-induced mycobacterial killing by macrophages. J. Biol. Chem. 281 (4), 2079–2086. 10.1074/jbc.M507816200 16263709

[B313] Shieh C. H. Heinrich A. Serchov T. van Calker D. Biber K. (2014). P2X7-dependent, but differentially regulated release of IL-6, CCL2, and TNF-alpha in cultured mouse microglia. Glia 62 (4), 592–607. 10.1002/glia.22628 24470356

[B314] Shiratori M. Tozaki-Saitoh H. Yoshitake M. Tsuda M. Inoue K. (2010). P2X7 receptor activation induces CXCL2 production in microglia through NFAT and PKC/MAPK pathways. J. Neurochem. 114 (3), 810–819. 10.1111/j.1471-4159.2010.06809.x 20477948

[B315] Silverman W. R. de Rivero Vaccari J. P. Locovei S. Qiu F. Carlsson S. K. Scemes E. (2009). The pannexin 1 channel activates the inflammasome in neurons and astrocytes. J. Biol. Chem. 284 (27), 18143–18151. 10.1074/jbc.M109.004804 19416975 PMC2709345

[B316] Sinadinos A. Young C. N. Al-Khalidi R. Teti A. Kalinski P. Mohamad S. (2015). P2RX7 purinoceptor: a therapeutic target for ameliorating the symptoms of duchenne muscular dystrophy. PloS Med. 12 (10), e1001888. 10.1371/journal.pmed.1001888 26461208 PMC4604078

[B317] Slater M. Barden J. A. (2005). Differentiating keratoacanthoma from squamous cell carcinoma by the use of apoptotic and cell adhesion markers. Histopathology 47 (2), 170–178. 10.1111/j.1365-2559.2005.02155.x 16045778

[B318] Slater M. Scolyer R. A. Gidley-Baird A. Thompson J. F. Barden J. A. (2003). Increased expression of apoptotic markers in melanoma. Melanoma Res. 13 (2), 137–145. 10.1097/01.cmr.0000056225.78713.42 12690296

[B319] Slater M. Danieletto S. Gidley-Baird A. Teh L. C. Barden J. A. (2004a). Early prostate cancer detected using expression of non-functional cytolytic P2X7 receptors. Histopathology 44 (3), 206–15. 10.1111/j.0309-0167.2004.01798.x 14987223

[B320] Slater M. Danieletto S. Pooley M. Cheng Teh L. Gidley-Baird A. Barden J. A. (2004b). Differentiation between cancerous and normal hyperplastic lobules in breast lesions. Breast Cancer Res. Treat 83 (1), 1–10. 10.1023/B:BREA.0000010670.85915.0f 14997049

[B321] Slater M. Danieletto S. Barden J. A. (2005). Expression of the apoptotic calcium channel P2X7 in the glandular epithelium. J. Mol. Histol. 36 (3), 159–165. 10.1007/s10735-004-6166-7 15900405

[B322] Sluyter R. Stokes L. (2011). Significance of P2X7 receptor variants to human health and disease. Recent Pat. DNA Gene Seq. 5 (1), 41–54. 10.2174/187221511794839219 21303345

[B323] Smart M. L. Gu B. Panchal R. G. Wiley J. Cromer B. Williams D. A. (2003). P2X7 receptor cell surface expression and cytolytic pore formation are regulated by a distal C-terminal region. J. Biol. Chem. 278 (10), 8853–8860. 10.1074/jbc.M211094200 12496266

[B324] Solini A. Chiozzi P. Morelli A. Fellin R. Di Virgilio F. (1999). Human primary fibroblasts in vitro express a purinergic P2X7 receptor coupled to ion fluxes, microvesicle formation and IL-6 release. J. Cell Sci. 112 (Pt 3), 297–305. 9885283 10.1242/jcs.112.3.297

[B325] Solini A. Cuccato S. Ferrari D. Santini E. Gulinelli S. Callegari M. G. (2008). Increased P2X7 receptor expression and function in thyroid papillary cancer: a new potential marker of the disease? Endocrinology 149 (1), 389–396. 10.1210/en.2007-1223 17947359

[B326] Solini A. Simeon V. Derosa L. Orlandi P. Rossi C. Fontana A. (2015). Genetic interaction of P2X7 receptor and VEGFR-2 polymorphisms identifies a favorable prognostic profile in prostate cancer patients. Oncotarget 6 (30), 28743–28754. 10.18632/oncotarget.4926 26337470 PMC4745689

[B327] Solle M. Labasi J. Perregaux D. G. Stam E. Petrushova N. Koller B. H. (2001). Altered cytokine production in mice lacking P2X(7) receptors. J. Biol. Chem. 276 (1), 125–132. 10.1074/jbc.M006781200 11016935

[B328] Sorge R. E. Trang T. Dorfman R. Smith S. B. Beggs S. Ritchie J. (2012). Genetically determined P2X7 receptor pore formation regulates variability in chronic pain sensitivity. Nat. Med. 18 (4), 595–599. 10.1038/nm.2710 22447075 PMC3350463

[B329] Steinberg T. H. Newman A. S. Swanson J. A. Silverstein S. C. (1987). ATP4- permeabilizes the plasma membrane of mouse macrophages to fluorescent dyes. J. Biol. Chem. 262 (18), 8884–8888. 3597398

[B330] Stock T. C. Bloom B. J. Wei N. Ishaq S. Park W. Wang X. (2012). Efficacy and safety of CE-224,535, an antagonist of P2X7 receptor, in treatment of patients with rheumatoid arthritis inadequately controlled by methotrexate. J. Rheumatol. 39 (4), 720–727. 10.3899/jrheum.110874 22382341

[B331] Stokes L. Jiang L. H. Alcaraz L. Bent J. Bowers K. Fagura M. (2006). Characterization of a selective and potent antagonist of human P2X(7) receptors, AZ11645373. Br. J. Pharmacol. 149 (7), 880–887. 10.1038/sj.bjp.0706933 17031385 PMC2014691

[B332] Stokes L. Fuller S. J. Sluyter R. Skarratt K. K. Gu B. J. Wiley J. S. (2010). Two haplotypes of the P2X(7) receptor containing the Ala-348 to Thr polymorphism exhibit a gain-of-function effect and enhanced interleukin-1beta secretion. FASEB J. 24 (8), 2916–2927. 10.1096/fj.09-150862 20360457

[B333] Sun C. Chu J. Singh S. Salter R. D. (2010). Identification and characterization of a novel variant of the human P2X(7) receptor resulting in gain of function. Purinerg. Signal 6 (1), 31–45. 10.1007/s11302-009-9168-9 PMC2837825 19838818

[B334] Sun L. Gao J. Zhao M. Cui J. Li Y. Yang X. (2015). A novel cognitive impairment mechanism that astrocytic p-connexin 43 promotes neuronic autophagy via activation of P2X7R and down-regulation of GLT-1 expression in the hippocampus following traumatic brain injury in rats. Behav. Brain Res. 291, 315–324. 10.1016/j.bbr.2015.05.049 26031379

[B335] Sun S. H. (2010). Roles of P2X7 receptor in glial and neuroblastoma cells: the therapeutic potential of P2X7 receptor antagonists. Mol. Neurobiol. 41 (2-3), 351–355. 10.1007/s12035-010-8120-x 20405342

[B336] Surprenant A. Rassendren F. Kawashima E. North R. A. Buell G. (1996). The cytolytic P2Z receptor for extracellular ATP identified as a P2X receptor (P2X7). Science 272 (5262), 735–738. 10.1126/science.272.5262.735 8614837

[B337] Swanson D. M. Savall B. M. Coe K. J. Schoetens F. Koudriakova T. Skaptason J. (2016). Identification of (R)-(2-Chloro-3-(trifluoromethyl)phenyl)(1-(5-fluoropyridin-2-yl)-4-methyl-6,7-di hydro-1H-imidazo[4,5-c]pyridin-5(4H)-yl)methanone (JNJ 54166060), a Small Molecule Antagonist of the P2X7 receptor. J. Med. Chem. 59 (18), 8535–8548. 10.1021/acs.jmedchem.6b00989 27548392

[B338] Tafani M. Russo A. Di Vito M. Sale P. Pellegrini L. Schito L. (2010). Up-regulation of pro-inflammatory genes as adaptation to hypoxia in MCF-7 cells and in human mammary invasive carcinoma microenvironment. Cancer Sci. 101 (4), 1014–1023. 10.1111/j.1349-7006.2010.01493.x 20151982 PMC11159242

[B339] Tafani M. Di Vito M. Frati A. Pellegrini L. De Santis E. Sette G. (2011). Pro-inflammatory gene expression in solid glioblastoma microenvironment and in hypoxic stem cells from human glioblastoma. J. Neuroinflammation 8, 32. 10.1186/1742-2094-8-32 21489226 PMC3098164

[B340] Takai E. Tsukimoto M. Harada H. Sawada K. Moriyama Y. Kojima S. (2012). Autocrine regulation of TGF-beta1-induced cell migration by exocytosis of ATP and activation of P2 receptors in human lung cancer cells. J. Cell Sci. 125 (Pt 21), 5051–5060. 10.1242/jcs.104976 22946048

[B341] Takai E. Tsukimoto M. Harada H. Kojima S. (2014). Autocrine signaling via release of ATP and activation of P2X7 receptor influences motile activity of human lung cancer cells. Purinerg. Signal 10 (3), 487–497. 10.1007/s11302-014-9411-x PMC4152450 24627191

[B342] Tamajusuku A. S. Villodre E. S. Paulus R. Coutinho-Silva R. Battasstini A. M. Wink M. R. (2010). Characterization of ATP-induced cell death in the GL261 mouse glioma. J. Cell Biochem. 109 (5), 983–991. 10.1002/jcb.22478 20069573

[B343] Tan C. Han L. I. Zou L. Luo C. Liu A. Sheng X. (2015). Expression of P2X7R in breast cancer tissue and the induction of apoptosis by the gene-specific shRNA in MCF-7 cells. Exp. Ther. Med. 10 (4), 1472–1478. 10.3892/etm.2015.2705 26622509 PMC4578102

[B344] Taylor S. R. Gonzalez-Begne M. Dewhurst S. Chimini G. Higgins C. F. Melvin J. E. (2008). Sequential shrinkage and swelling underlie P2X7-stimulated lymphocyte phosphatidylserine exposure and death. J. Immunol. 180 (1), 300–308. 10.4049/jimmunol.180.1.300 18097031

[B345] Tsukimoto M. Tokunaga A. Harada H. Kojima S. (2009). Blockade of murine T cell activation by antagonists of P2Y6 and P2X7 receptors. Biochem. Biophys. Res. Commun. 384 (4), 512–518. 10.1016/j.bbrc.2009.05.011 19426712

[B346] Ulrich H. Ratajczak M. Z. Schneider G. Adinolfi E. Orioli E. Ferrazoli E. G. (2018). Kinin and Purine Signaling Contributes to Neuroblastoma Metastasis. Front. Pharmacol. 9, 500. 10.3389/fphar.2018.00500 29867502 PMC5968427

[B347] Valera S. Hussy N. Evans R. J. Adami N. North R. A. Surprenant A. (1994). A new class of ligand-gated ion channel defined by P2x receptor for extracellular ATP. Nature 371 (6497), 516–519. 10.1038/371516a0 7523951

[B348] Vazquez-Cuevas F. G. Martinez-Ramirez A. S. Robles-Martinez L. Garay E. Garcia-Carranca A. Perez-Montiel D. (2014). Paracrine stimulation of P2X7 receptor by ATP activates a proliferative pathway in ovarian carcinoma cells. J. Cell Biochem. 115 (11), 1955–1966. 10.1002/jcb.24867 24913779

[B349] Vijayan D. Young A. Teng M. W. L. Smyth M. J. (2017). Targeting immunosuppressive adenosine in cancer. Nat. Rev. Cancer 17 (12), 709–724. 10.1038/nrc.2017.86 29059149

[B350] Virginio C. MacKenzie A. North R. A. Surprenant A. (1999). Kinetics of cell lysis, dye uptake and permeability changes in cells expressing the rat P2X7 receptor. J. Physiol. 519 Pt 2, 335–346. 10.1111/j.1469-7793.1999.0335m.x 10457053 PMC2269518

[B351] Vivarelli S. Salemi R. Candido S. Falzone L. Santagati M. Stefani S. (2019). Gut Microbiota and Cancer: From Pathogenesis to Therapy. Cancers (Basel) 11 (1), 38. 10.3390/cancers11010038 PMC6356461 30609850

[B352] Walev I. Reske K. Palmer M. Valeva A. Bhakdi S. (1995). Potassium-inhibited processing of IL-1 beta in human monocytes. EMBO J. 14 (8), 1607–1614. 10.1002/j.1460-2075.1995.tb07149.x 7737113 PMC398253

[B353] Wang B. Sluyter R. (2013). P2X7 receptor activation induces reactive oxygen species formation in erythroid cells. Purinerg. Signal 9 (1), 101–112. 10.1007/s11302-012-9335-2 PMC3568428 23014887

[B354] Wang L. Jacobsen S. E. Bengtsson A. Erlinge D. (2004). P2 receptor mRNA expression profiles in human lymphocytes, monocytes and CD34+ stem and progenitor cells. BMC Immunol. 5, 16. 10.1186/1471-2172-5-16 15291969 PMC509419

[B355] Wang W. Xiao J. Adachi M. Liu Z. Zhou J. (2011). 4-aminopyridine induces apoptosis of human acute myeloid leukemia cells via increasing [Ca2+]i through P2X7 receptor pathway. Cell Physiol. Biochem. 28 (2), 199–208. 10.1159/000331731 21865727

[B356] Wei W. Ryu J. K. Choi H. B. McLarnon J. G. (2008). Expression and function of the P2X(7) receptor in rat C6 glioma cells. Cancer Lett. 260 (1-2), 79–87. 10.1016/j.canlet.2007.10.025 18039556

[B357] Wesselius A. Bours M. J. Arts I. C. Theunisz E. H. Geusens P. Dagnelie P. C. (2012). The P2X(7) loss-of-function Glu496Ala polymorphism affects ex vivo cytokine release and protects against the cytotoxic effects of high ATP-levels. BMC Immunol. 13, 64. 10.1186/1471-2172-13-64 23210974 PMC3526505

[B358] White N. Butler P. E. Burnstock G. (2005). Human melanomas express functional P2 X(7) receptors. Cell Tissue Res. 321 (3), 411–418. 10.1007/s00441-005-1149-x 15991050

[B359] White E. Mehnert J. M. Chan C. S. (2015). Autophagy, Metabolism, and Cancer. Clin. Cancer Res. 21 (22), 5037–5046. 10.1158/1078-0432.CCR-15-0490 26567363 PMC4646728

[B360] Wickert L. E. Blanchette J. B. Waldschmidt N. V. Bertics P. J. Denu J. M. Denlinger L. C. (2013). The C-terminus of human nucleotide receptor P2X7 is critical for receptor oligomerization and N-linked glycosylation. PloS One 8 (5), e63789. 10.1371/journal.pone.0063789 23691096 PMC3653848

[B361] Wiley J. S. Dao-Ung L. P. Gu B. J. Sluyter R. Shemon A. N. Li C. (2002). A loss-of-function polymorphic mutation in the cytolytic P2X7 receptor gene and chronic lymphocytic leukaemia: a molecular study. Lancet 359 (9312), 1114–1119. 10.1016/S0140-6736(02)08156-4 11943260

[B362] Wiley J. S. Dao-Ung L. P. Li C. Shemon A. N. Gu B. J. Smart M. L. (2003). An Ile-568 to Asn polymorphism prevents normal trafficking and function of the human P2X7 receptor. J. Biol. Chem. 278 (19), 17108–17113. 10.1074/jbc.M212759200 12586825

[B363] Wiley J. S. Sluyter R. Gu B. J. Stokes L. Fuller S. J. (2011). The human P2X7 receptor and its role in innate immunity. Tissue Antigens 78 (5), 321–332. 10.1111/j.1399-0039.2011.01780.x 21988719

[B364] Wilkaniec A. Gassowska M. Czapski G. A. Cieslik M. Sulkowski G. Adamczyk A. (2017). P2X7 receptor-pannexin 1 interaction mediates extracellular alpha-synuclein-induced ATP release in neuroblastoma SH-SY5Y cells. Purinerg. Signal. 13 (3), 347–361. 10.1007/s11302-017-9567-2 PMC5563296 28516276

[B365] Wilson H. L. Wilson S. A. Surprenant A. North R. A. (2002). Epithelial membrane proteins induce membrane blebbing and interact with the P2X7 receptor C terminus. J. Biol. Chem. 277 (37), 34017–34023. 10.1074/jbc.M205120200 12107182

[B366] Woehrle T. Yip L. Elkhal A. Sumi Y. Chen Y. Yao Y. (2010). Pannexin-1 hemichannel-mediated ATP release together with P2X1 and P2X4 receptors regulate T-cell activation at the immune synapse. Blood 116 (18), 3475–3484. 10.1182/blood-2010-04-277707 20660288 PMC2981474

[B367] Xia J. Yu X. Tang L. Li G. He T. (2015). P2X7 receptor stimulates breast cancer cell invasion and migration via the AKT pathway. Oncol. Rep. 34 (1), 103–110. 10.3892/or.2015.3979 25976617

[B368] Yan Z. Khadra A. Li S. Tomic M. Sherman A. Stojilkovic S. S. (2010). Experimental characterization and mathematical modeling of P2X7 receptor channel gating. J. Neurosci. 30 (42), 14213–14224. 10.1523/JNEUROSCI.2390-10.2010 20962242 PMC2980950

[B369] Yan J. Li X. Y. Roman Aguilera A. Xiao C. Jacoberger-Foissac C. Nowlan B. (2020). Control of Metastases via Myeloid CD39 and NK Cell Effector Function. Cancer Immunol. Res. 8 (3), 356–367. 10.1158/2326-6066.CIR-19-0749 31992567

[B370] Yang Y. C. Chang T. Y. Chen T. C. Lin W. S. Chang S. C. Lee Y. J. (2016). Functional variant of the P2X7 receptor gene is associated with human papillomavirus-16 positive cervical squamous cell carcinoma. Oncotarget 7 (50), 82798–82803. 10.18632/oncotarget.12636 27779103 PMC5347733

[B371] Yang J. Ma C. Zhang M. (2019). High glucose inhibits osteogenic differentiation and proliferation of MC3T3E1 cells by regulating P2X7. Mol. Med. Rep. 20 (6), 5084–5090. 10.3892/mmr.2019.10790 31702818 PMC6854521

[B372] Yip L. Woehrle T. Corriden R. Hirsh M. Chen Y. Inoue Y. (2009). Autocrine regulation of T-cell activation by ATP release and P2X7 receptors. FASEB J. 23 (6), 1685–1693. 10.1096/fj.08-126458 19211924 PMC2718802

[B373] Yoon M. J. Lee H. J. Kim J. H. Kim D. K. (2006). Extracellular ATP induces apoptotic signaling in human monocyte leukemic cells, HL-60 and F-36P. Arch. Pharm. Res. 29 (11), 1032–1041. 10.1007/BF02969288 17146973

[B374] Young C. N. Sinadinos A. Lefebvre A. Chan P. Arkle S. Vaudry D. (2015). A novel mechanism of autophagic cell death in dystrophic muscle regulated by P2RX7 receptor large-pore formation and HSP90. Autophagy 11 (1), 113–130. 10.4161/15548627.2014.994402 25700737 PMC4502824

[B375] Young C. N. J. Chira N. Rog J. Al-Khalidi R. Benard M. Galas L. (2018). Sustained activation of P2X7 induces MMP-2-evoked cleavage and functional purinoceptor inhibition. J. Mol. Cell Biol. 10 (3), 229–242. 10.1093/jmcb/mjx030 28992079

[B376] Yu T. Junger W. G. Yuan C. Jin A. Zhao Y. Zheng X. (2010). Shockwaves increase T-cell proliferation and IL-2 expression through ATP release, P2X7 receptors, and FAK activation. Am. J. Physiol. Cell Physiol. 298 (3), C457–C464. 10.1152/ajpcell.00342.2009 19889958 PMC4631534

[B377] Zanovello P. Bronte V. Rosato A. Pizzo P. Di Virgilio F. (1990). Responses of mouse lymphocytes to extracellular ATP. II. Extracellular ATP causes cell type-dependent lysis and DNA fragmentation. J. Immunol. 145 (5), 1545–1550. 2384670

[B378] Zhang L. Y. Ibbotson R. E. Orchard J. A. Gardiner A. C. Seear R. V. Chase A. J. (2003). P2X7 polymorphism and chronic lymphocytic leukaemia: lack of correlation with incidence, survival and abnormalities of chromosome 12. Leukemia 17 (11), 2097–2100. 10.1038/sj.leu.2403125 12931211

[B379] Zhang X. J. Zheng G. G. Ma X. T. Yang Y. H. Li G. Rao Q. (2004). Expression of P2X7 in human hematopoietic cell lines and leukemia patients. Leuk. Res. 28 (12), 1313–1322. 10.1016/j.leukres.2004.04.001 15475073

[B380] Zhang Y. Cheng H. Li W. Wu H. Yang Y. (2019a). Highly-expressed P2X7 receptor promotes growth and metastasis of human HOS/MNNG osteosarcoma cells via PI3K/Akt/GSK3beta/beta-catenin and mTOR/HIF1alpha/VEGF signaling. Int. J. Cancer 145 (4), 1068–1082. 10.1002/ijc.32207 30761524 PMC6618011

[B381] Zhang Y. Ding J. Wang L. (2019b). The role of P2X7 receptor in prognosis and metastasis of colorectal cancer. Adv. Med. Sci. 64 (2), 388–394. 10.1016/j.advms.2019.05.002 31276917

[B382] Zhao Q. Yang M. Ting A. T. Logothetis D. E. (2007). PIP(2) regulates the ionic current of P2X receptors and P2X(7) receptor-mediated cell death. Channels (Austin) 1 (1), 46–55. 10.4161/chan.3914 19151591

[B383] Zheng L. Zhang X. Yang F. Zhu J. Zhou P. Yu F. (2014). Regulation of the P2X7R by microRNA-216b in human breast cancer. Biochem. Biophys. Res. Commun. 452 (1), 197–204. 10.1016/j.bbrc.2014.07.101 25078617

[B384] Zitvogel L. Ma Y. Raoult D. Kroemer G. Gajewski T. F. (2018). The microbiome in cancer immunotherapy: Diagnostic tools and therapeutic strategies. Science 359 (6382), 1366–1370. 10.1126/science.aar6918 29567708

[B385] Zumerle S. Cali B. Munari F. Angioni R. Di Virgilio F. Molon B. (2019). Intercellular Calcium Signaling Induced by ATP Potentiates Macrophage Phagocytosis. Cell Rep. 27 (1), 1–10 e4. 10.1016/j.celrep.2019.03.011 30943393 PMC6449513

